# ATP-Triggered Fe(CN)_2_CO Synthon Transfer from the Maturase HypCD to the Active Site of Apo-[NiFe]-Hydrogenase

**DOI:** 10.1021/jacs.4c09791

**Published:** 2024-11-04

**Authors:** Anna Kwiatkowski, Giorgio Caserta, Anne-Christine Schulz, Stefan Frielingsdorf, Vladimir Pelmenschikov, Kilian Weisser, Adam Belsom, Juri Rappsilber, Ilya Sergueev, Christian Limberg, Maria-Andrea Mroginski, Ingo Zebger, Oliver Lenz

**Affiliations:** † Institut für Chemie, Technische Universität Berlin, Straße des 17. Juni 135, 10623 Berlin, Germany; ‡ Institute of Chemistry, Humboldt-Universität zu Berlin, Brook-Taylor-Straße 2, 12489 Berlin, Germany; § Institute of Biotechnology, Chair of Bioanalytics, Technische Universität Berlin, Gustav-Meyer-Allee 25, 13355 Berlin, Germany; ∥ Si-M/‘Der Simulierte Mensch’, a Science Framework of Technische Universität Berlin and Charité − Universitätsmedizin Berlin, 10623 Berlin, Germany; ⊥ Wellcome Centre of Cell Biology, University of Edinburgh, Edinburgh EH9 3BF, U.K.; # Deutsches Elektronen-Synchrotron, Notkestraße 85, 22607 Hamburg, Germany

## Abstract

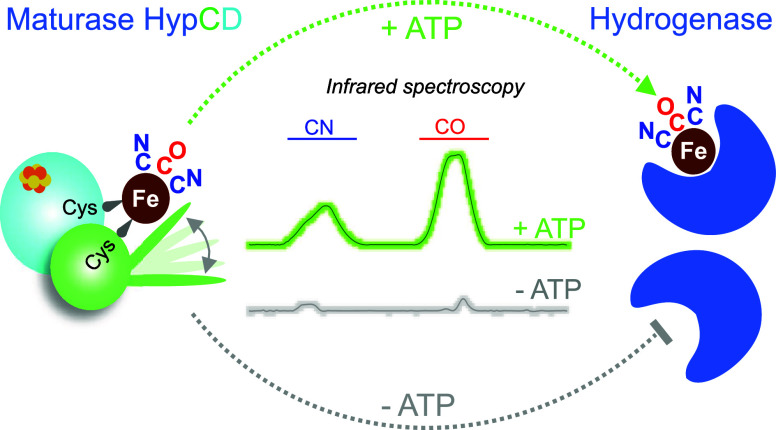

[NiFe]-hydrogenases catalyze the reversible activation of H_2_ using a unique NiFe(CN)_2_CO metal site, which is assembled by a sophisticated multiprotein machinery. The [4Fe–4S] cluster-containing HypCD complex, which possesses an ATPase activity with a hitherto unknown function, serves as the hub for the assembly of the Fe(CN)_2_CO subfragment. HypCD is also thought to be responsible for the subsequent transfer of the iron fragment to the apo-form of the catalytic hydrogenase subunit, but the underlying mechanism has remained unexplored. Here, we performed a thorough spectroscopic characterization of different HypCD preparations using infrared, Mössbauer, and NRVS spectroscopy, revealing molecular details of the coordination of the Fe(CN)_2_CO fragment. Moreover, biochemical assays in combination with spectroscopy, AlphaFold structure predictions, protein–ligand docking calculations, and crosslinking MS deciphered unexpected mechanistic aspects of the ATP requirement of HypCD, which we found to actually trigger the transfer of the Fe(CN)_2_CO fragment to the apo-hydrogenase.

## Introduction

By making use of earth-abundant nickel and iron ions, [NiFe]-hydrogenases catalyze the reversible activation of H_2_ close to the thermodynamic potential under ambient conditions.^1^ The functional unit of [NiFe]-hydrogenases features a bipartite architecture consisting of a catalytically competent large subunit that carries the heterobimetallic NiFe(CN)_2_CO active site and a small subunit, which contains one to three Fe–S clusters acting as an electric wire. The synthesis and assembly of the NiFe(CN)_2_CO cofactor requires an intricate protein machinery comprising at least six Hyp proteins named HypA–F.^2−5^ The current biosynthesis model envisages the [4Fe–4S] cluster-containing HypCD protein complex as a hub for the assembly of the Fe(CN)_2_CO synthon of the catalytic site (Figure S1). The CN^–^ strong field ligands are synthesized from carbamoylphosphate by the maturases HypF and HypE^6^ and are supposed to be transferred by the latter to a single iron ion transiently bound by HypCD. The aerobic biosynthesis of the CO ligand from *N*^10^-formyl-tetrahydrofolate has recently been uncovered,^7,8^ while the corresponding anoxic pathway remains elusive.^9,10^ The assembled Fe(CN)_2_CO synthon is then transferred to the apo-form of the large hydrogenase subunit via an unknown mechanism. Only then do the maturases HypA and HypB complete the active site metalation by mediating the insertion of a Ni ion.^11^ Finally, the fully metalated hydrogenase large subunit is usually processed by endoproteolytic cleavage of its C-terminal extension.^12−14^ This step has been shown to trigger structural changes at the NiFe site (e.g., incorporation of an OH^–^ ligand bridging Ni and Fe),^15^ which also allows the association of the large and small subunits to form catalytically competent [NiFe]-hydrogenase.^13^

Although the biosynthesis of the NiFe site has been examined in great detail,^15,16^ several aspects of the maturation process are still unclear. Among them, the mechanism by which the Fe(CN)_2_CO fragment is transferred from the HypCD complex to the apo-large subunit is essentially unknown. Recent work from the Sawers group has revealed an unexpected ATPase activity of the HypCD complex.^17,18^ Although the role of ATP hydrolysis in either the biosynthesis of the CO ligand or the transfer of the Fe(CN)_2_CO unit into the large subunit of the hydrogenase was hypothesized, there was no experimental evidence for this and for a possible ATP binding site.

In the present study, we extend our knowledge of [NiFe]-hydrogenase maturation to new aspects. We developed an *in vitro* assay with purified HypCD from *Escherichia coli* and the apo-large subunit HoxC of the regulatory [NiFe]-hydrogenase (RH) from *Cupriavidus necator* to investigate the role of the ATPase activity of HypCD in hydrogenase maturation. To this end, we first comprehensively characterized the purified HypCD complex by infrared (IR) and Mössbauer spectroscopy, which verified the integrity and stoichiometric loading with the Fe(CN)_2_CO synthon. Nuclear resonance vibrational spectroscopy (NRVS) in combination with density functional theory (DFT) provided details of the synthon bonding and a realistic structural model of holo-HypCD, which is consistent with the available biochemical and spectroscopic data. Protein–ligand docking calculations revealed that the most probable binding site of ATP overlaps with the proposed binding site of the Fe(CN)_2_CO synthon in HypCD. Using the *in vitro* transfer assay, we were able to probe and elucidate the role of ATP, showing that nucleotide binding triggers the transfer of the Fe(CN)_2_CO synthon from purified holo-HypCD to an apo-large subunit of the hydrogenase. AlphaFold-based structure predictions in combination with crosslinking mass spectrometry (crosslinking MS) indicate ATP-dependent structural changes within the HypCD complex. From these results, we developed a molecular model for the ATP-mediated transfer of the Fe(CN)_2_CO synthon to the apo-form of the hydrogenase large subunit.

## Results and Discussion

### Isolation and Spectroscopic Characterization of the HypCD and HoxC Proteins

Holo- and apo-HypC_Strep_-HypD protein complexes (Strep-tag II C-terminally fused to HypC hereafter referred to as holo- and apo-HypC_S_-D) were isolated by Strep-Tactin-based affinity chromatography from soluble extracts (Table S1) of aerobically cultivated *E. coli* (*Ec*) cells carrying pTHypDC_Strep_ and pTHypDEFC_Strep_ expression plasmids (Table S2).^19,20^ SDS-PAGE analysis of the isolated protein complexes showed the typical bands for HypC and HypD (Figure S2), which is consistent with previous studies.^19^ Apo-_Strep_HoxC (apo-_S_HoxC; Strep-tag II N-terminally fused to HoxC) from *Cupriavidus necator* (*Cn*) was heterologously overproduced in *E. coli* cells bearing the expression plasmid pTS17 (Table S2) and also isolated via Strep-Tactin-based affinity chromatography. The isolated proteins (complexes) were characterized by IR spectroscopy in the 2150–1800 cm^–1^ range to verify the presence of CO and CN^–^ ligands usually observed in hydrogenase maturation intermediates and fully assembled [NiFe]-hydrogenases.^1,15^ Clear IR absorption signals were exclusively detected for holo-HypC_S_-D (Figure S3). The observed ν_CN_ bands at 2098 and 2072 cm^–1^ and ν_CO_ bands at 1951 and 1963 cm^–1^ resemble those previously reported,^20,21^ suggesting the isolation of a native-like HypCD complex. The metal content of HypC_S_-D was then determined by inductively coupled plasma-optical emission spectroscopy (ICP-OES). The iron content of holo-HypC_S_-D was determined with 6.64 ± 0.09 and that of apo-HypC_S_-D with 5.37 ± 0.06. For unknown reasons, the total iron content of both proteins exceeds the theoretical iron content by 1–1.5 iron. Importantly, both proteins differed as expected by about one iron ion, which can presumably be assigned to the species equipped with the CO/CN^–^ ligands (see below). To test whether both HypD and HypC are required for assembly and binding of the Fe(CN)_2_CO fragment, we purified the two proteins independently, and the corresponding IR measurements did not reveal any detectable CO/CN^–^ absorptions (Figure S4). These data, therefore, strongly suggest that the assembled holo-HypCD complex is required for Fe(CN)_2_CO fragment synthesis.

### Characterization of the Fe(CN)_2_CO Fragment in HypCD

**Figure 1:**
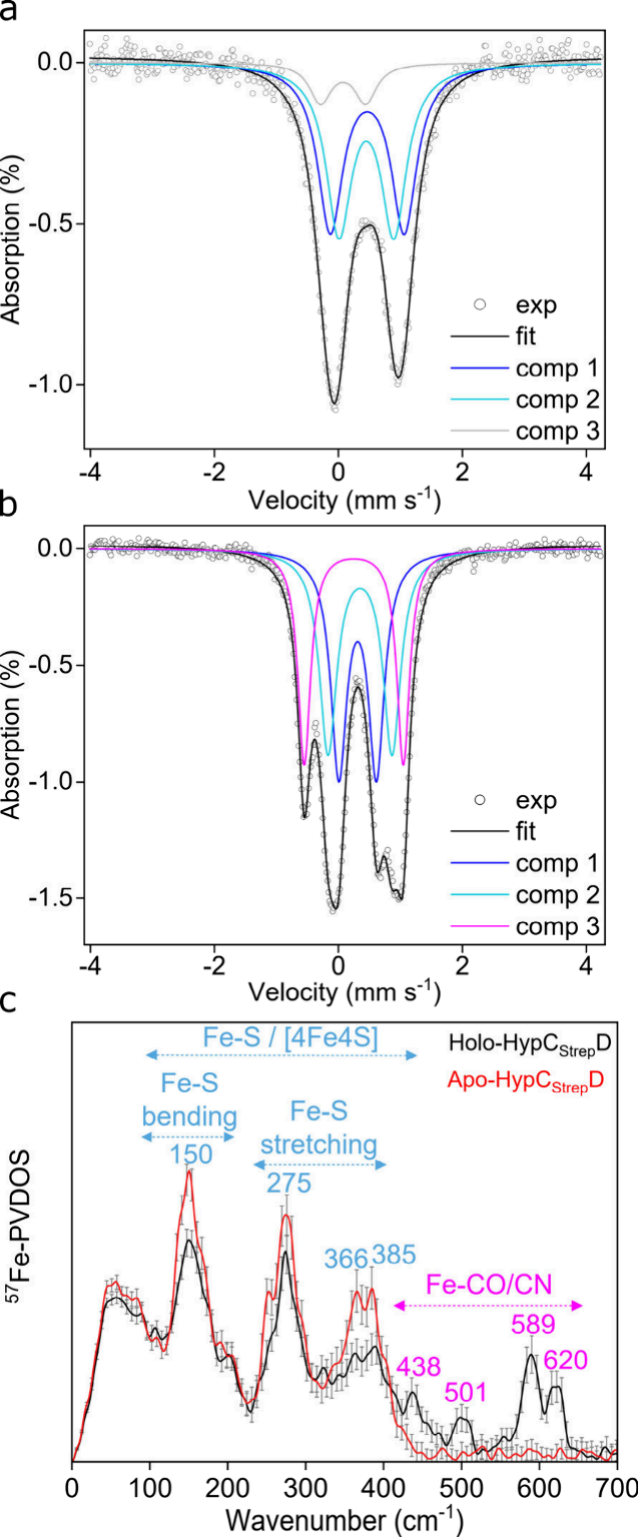
Mössbauer and NRVS characterization of apo- and holo-HypCD. Experimental Mössbauer data for (a) apo- and (b) holo-HypCD are depicted as single data points (circles). Black lines correspond to the curve fits containing all spectral components; the dark blue (component 1) and light blue (component 2) lines correspond to the two Fe^2.5+^–Fe^2.5+^ pairs of the [4Fe–4S]^2+^ cluster. The gray line (component 3) in panel a represents the spectral contribution of a [3Fe–4S] cluster (minor) in apo-HypCD. The magenta line in panel b corresponds to the Fe^II^(CN)_2_CO species in holo-HypCD. (c) NRVS of apo-HypCD (red trace) showing Fe–S bending/stretching bands originating from the [4Fe–4S] cluster (blue dashed arrows). The contribution of the Fe(CN)_2_CO fragment is detectable only in the holo-HypCD spectrum (black trace), characterized by bands in the Fe–CN/CO region (magenta dashed arrow).

The Fe(CN)_2_CO fragment of the hydrogenase active site has been shown to be EPR-silent, which has been interpreted as a Fe^II^ low-spin configuration.^1^ Previous Mössbauer data on holo-HypCD showed small resonance signals attributed to a low-spin Fe^II^ species, but their origin was not confirmed by measurements on an apo-HypCD construct without the Fe(CN)_2_CO synthon.^22^ Therefore, we recorded zero-field Mössbauer spectra for both apo- and holo-HypC_S_-D enriched with ^57^Fe (see the Methods section). Resonance signals of both samples were simulated with different spectral components, which are shown in Figure 1a,b. The main signals in both holo- and apo-HypC_S_-D are typical of a quadrupole doublet consisting of two components, each representing a Fe^2.5+^–Fe^2.5+^ pair of a [4Fe–4S]^2+^ cluster. The isomer shifts and quadrupole splitting parameters are given in Table S3. The parameters of the [4Fe–4S] cluster in both apo- and holo-HypC_S_-D are almost identical to those previously reported.^22,23^ Besides minor signals of a [3Fe–4S]^+^ species,^24^ the fit used to simulate the apo-HypC_S_-D spectrum did not require any additional spectral components. In contrast, holo-HypC_S_-D contains an additional and significant resonance characterized by an isomer shift of δ = 0.25 mm s^–1^ and a quadrupole splitting of Δ*E*_Q_ = 1.60 mm s^–1^. For [NiFe]-hydrogenases, these parameters have been associated with a 5-fold coordinated low-spin Fe^II^ species equipped with one CO and two CN^–^ ligands.^22,25^ The intensity of this resonance appears considerably higher than that previously reported,^22^ indicating a significantly higher (^57^Fe) occupancy of the low-spin Fe^II^ species in our protein preparations. Our data show that both the [4Fe–4S] cluster and the Fe(CN)_2_CO synthon are incorporated stoichiometrically into HypCD, even under aerobic conditions.

To shed light on the coordination of iron ions, we employed nuclear resonance vibrational spectroscopy (NRVS). This synchrotron technique provides vibrational dynamics for Mössbauer-active nuclei, such as the ^57^Fe isotope. Typical NRVS data of [NiFe]-hydrogenases comprise dominant Fe–S stretching and bending modes (100–420 cm^–1^) of the [Fe–S] clusters of the small subunit as well as Fe–CO/CN stretching and bending modes of the NiFe active site (400–650 cm^–1^).^26,27^

Depending on the (redox) state of the active site, modes of bridging hydride and hydroxide ligands have also been assigned.^28,29^ Apo-HypC_S_-D predominantly showed signals that can be assigned to a [4Fe–4S] cluster (Figure 1c). Major bands were detected at 150 cm^–1^ (bending and breathing modes of the cluster), as well as at 275, 366, and 385 cm^–1^, which represent normal modes with predominant Fe–S stretching character. These signals appeared at positions similar to those observed for the [4Fe–4S] cluster-containing ferredoxin (D14C variant) from *Pyrococcus furiosus*.^30^ Holo-HypC_S_-D, on the other hand, exhibited additional absorptions at 438, 501, 589, and 620 cm^–1^, which originate from modes involving the CO/CN^–^ ligands of the Fe(CN)_2_CO synthon (see next paragraph). In conclusion, our Mössbauer and NRVS data convincingly show that holo-HypCD harbors two iron cofactors, a [4Fe–4S] cluster and a low-spin Fe^II^(CN)_2_CO fragment. Strikingly, the high-frequency region of the NRVS spectrum in holo-HypC_S_-D resembles that of the nickel-free maturation intermediate of the membrane-bound hydrogenase (MBH) large subunit from *C. necator*(15) (Figure S5). This intermediate has been shown to bind a 5-fold coordinated Fe(CN)_2_CO fragment, presumably via two of the four conserved cysteine residues of the hydrogenase active site. Given the similarity of the high-frequency spectral range of the two proteins, a comparable coordination can be inferred for the Fe^II^(CN)_2_CO species in both holo-HypCD and the nickel-free large hydrogenase subunit. This scenario is in agreement with prior computational and biochemical data suggesting that the Fe(CN)_2_CO fragment in HypCD is coordinated by one cysteine residue from HypC and one from HypD.^31,32^

### Binding Site of the Fe(CN)_2_CO Fragment in HypCD

**Figure 2:**
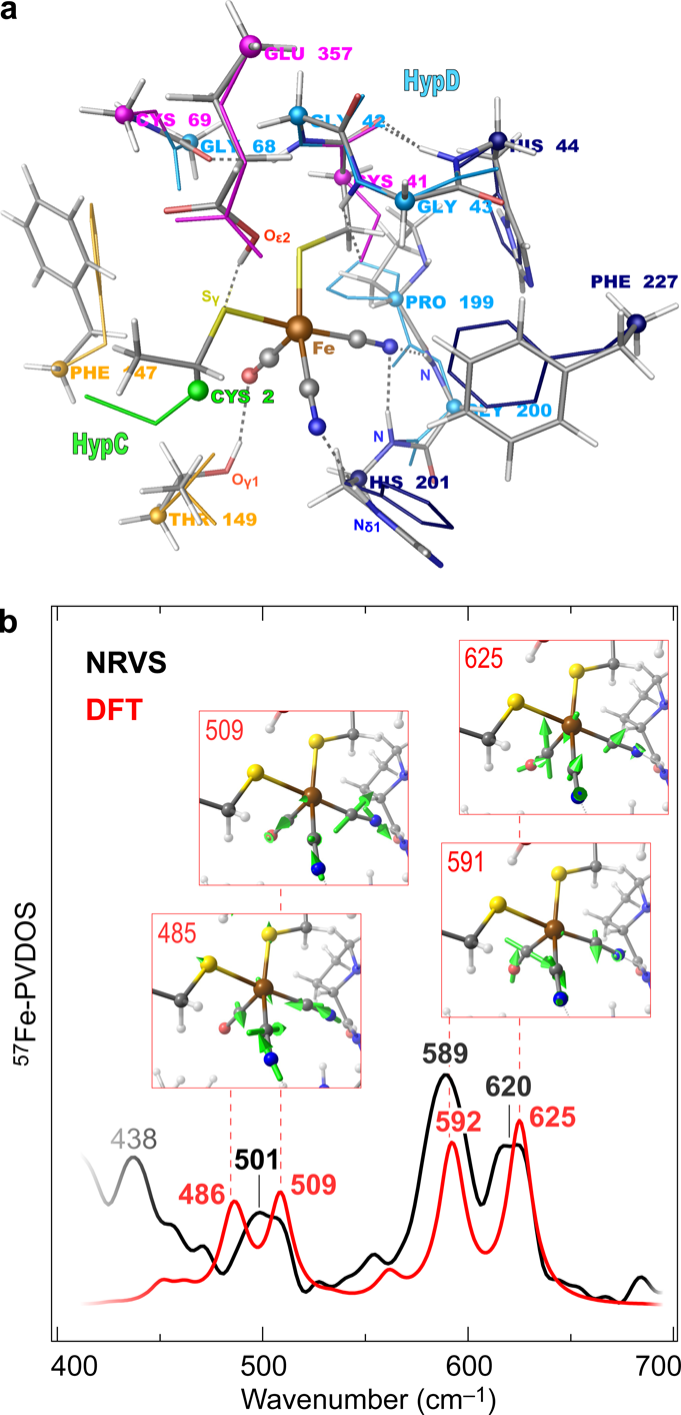
DFT modeling of the Fe(CN)_2_CO cofactor binding site in *Ec*HypCD and the corresponding NRVS spectrum. (a) The optimized DFT structure (tube representation, element colors) is overlaid with the original positions of the amino acid residues in the apo-*Ec*HypCD AlphaFold model (wire representation). The color codes for the reference residues of the AlphaFold model are as follows: HypC: Cys2, light green. HypD: Cys41/Cys69/Glu357, important for the assembly of the Fe(CN)_2_CO cofactor,^32,33^ magenta; Phe147/Thr149 of the conserved GF_147_ET_149_T motif, orange; His44/His201/Phe227 matching three residues within 3 Å from the cofactor in the *Tk*HypCD model by Albareda et al.,^31^ dark blue; Gly42/Gly43/Gly68/Pro199/Gly200, light blue. Individual atoms participating in hydrogen bonding (···) with the cofactor are named. C_α_ and Fe(CN)_2_CO fragment atoms are shown as spheres. (b) Predicted (DFT, red trace) and experimental (NRVS, black trace) ^57^Fe-PVDOS of holo-*Ec*HypCD in the 400–700 cm^–1^ spectral range. Selected vibrational modes are shown in an arrow-style representation above the spectra. Animations of these and other normal modes are available as part of the Supplementary Data III. An extended version of panel b is available in Figure S7.

The binding of the Fe(CN)_2_CO cofactor in *Ec*HypCD was further analyzed using density functional theory (DFT) calculations (see the Methods section). We adopted the previously proposed low-spin ferrous Fe^II^ center coordination by the thiolates of Cys2_HypC_ and Cys41_HypD_ (Figure 2a)^31,32^ as a constraint for our DFT modeling. The protein environment of the Fe(CN)_2_CO fragment was based on the cofactor-free AlphaFold structure of *Ec*HypCD generated as described in the Methods section. Notably, the incorporation of the Fe(CN)_2_CO fragment was achieved without apparent steric clashes. Only moderate changes in the amino acid side chain positions were produced by the subsequent DFT structure optimization. The refined model of holo-*Ec*HypCD in comparison to the initial AlphaFold structure is shown in Figure 2a and Figure S6 (with conserved motifs of HypD highlighted). The position of the Fe(CN)_2_CO synthon in *Ec*HypCD is reminiscent of that in a previous model of holo-HypCD from *Thermococcus kodakarensis* (*Tk*).^31,32^ In our model, the diatomic CN^–^ and CO ligands form hydrogen bonds with HypD residues His201, Gly200, and Thr149. Furthermore, Glu357 from HypD forms a hydrogen bond with the sulfur atom of Cys2 from HypC (Figure 2a). The specific contacts are (C_2_)S_γ_···H–O_ε2_(E_357_) = 3.08 Å, CN···H–N_δ1_(H_201_) = 2.87 Å, CN···H–N(H_201_/G_200_) = 3.18/2.91 Å, and CO···H–O_γ1_(T_149_) = 2.99 Å.

The DFT model of holo-*Ec*HypCD was also used to simulate the ^57^Fe-PVDOS (partial vibrational densities of states) contribution of the Fe(CN)_2_CO fragment to the NRVS spectrum discussed above and shown in Figure 1c. Figure 2b shows that the calculations reproduce the high-energy spectral region from 450 to 650 cm^–1^, which contains mixed modes of predominantly stretching Fe–CO/CN and bending Fe–C–O/N characters. The two modes of the strongest symmetric/antisymmetric Fe–S(Cys2_HypC_/Cys41_HypD_) stretching character were calculated at 390 and 366 cm^–1^ (Figure S7). Interestingly, these Fe–S modes of the cysteine-bound Fe(CN)_2_CO cofactor produce only very low ^57^Fe-PVDOS intensities, in contrast to the corresponding vibrational energy regions of Fe–S clusters^30^ such as the [4Fe–4S]^2+^ cubane of HypCD (Figure 1c and Figure S5). Thus, our normal-mode analysis suggests that the principal Fe–S stretches of holo-*Ec*HypCD are associated with *rotations* of the FeC_3_ core instead of *displacements* of the Fe center. The presence of “NRVS-silent” Fe–S stretches in holo-*Ec*HypCD agrees well with the intensity level near the baseline around 350–400 cm^–1^ of the nickel-free hydrogenase large subunit intermediate^15^ (Figure S5), which is expected to bind the Fe(CN)_2_CO fragment in a similar manner as HypCD (see above).

### ATP Binding Site in HypCD

**Figure 3:**
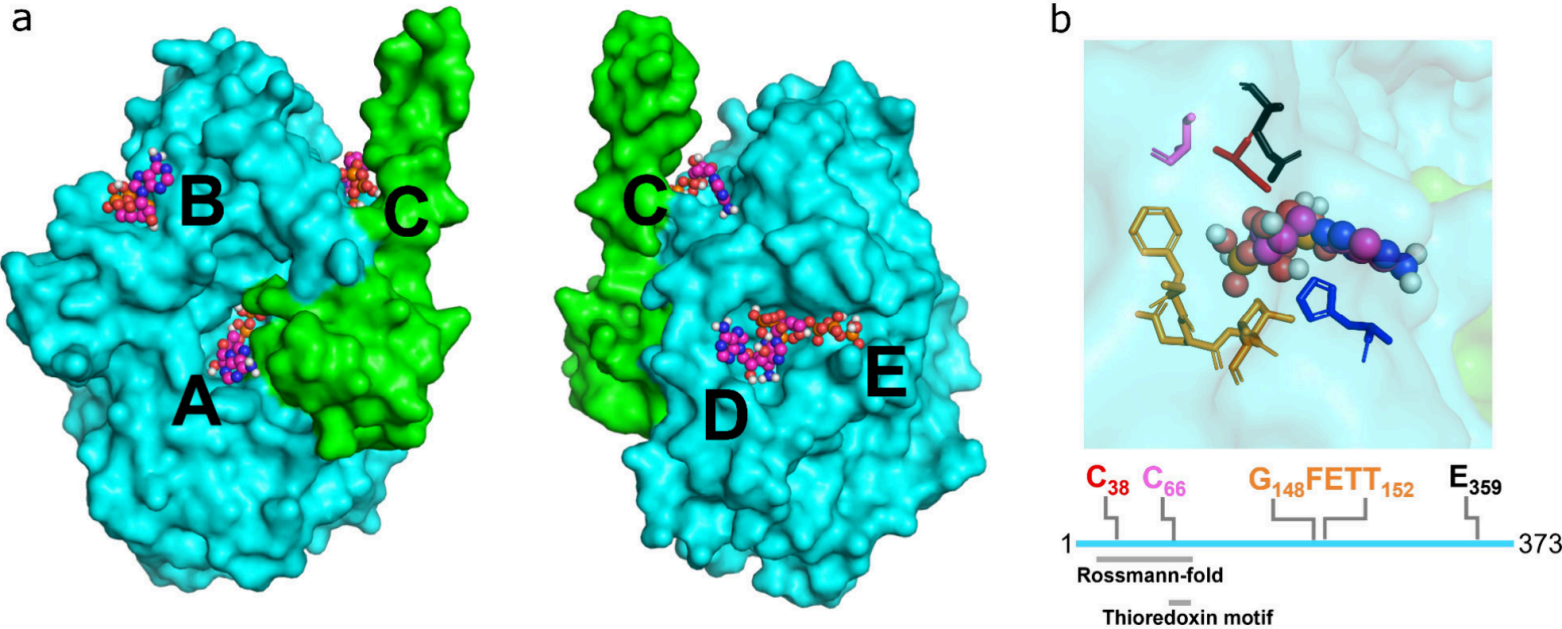
Predicted docking sites for ATP in *T. kodakarensis* HypCD. (a) ATP-binding locations as predicted by CB-Dock2 are labeled alphabetically (A–E). Vina Scores: Cavity A, −8.3; B, −5.7; C, −6.3; D, −5.5; E, −5.9. HypD is depicted in cyan, and HypC is in green. ATP is shown as spheres. (b) Close-up of the predicted connection residues to ATP in cavity A, including the conserved residues Cys38 (red), Cys66 (magenta), and Glu359 (black) from HypD and His45 (blue) from HypC.^32,33,37,38^ The highly conserved GFETT motif (orange) in HypD (Figures S10 and S11) is also part of cavity A, with connections to the ATP molecule. A representation of the amino acid sequence of HypD illustrates the affiliation of individual amino acid residues to the Rossman fold and/or the thioredoxin motif (gray bars) of HypD.

Both the apo- and holo-forms of anaerobically purified HypCD and the paralogous HybG–HypD complex from *E. coli* have been shown to hydrolyze ATP.^17,18^ However, the ATP binding site and the biological function of ATPase activity remained unknown. To shed light on the possible binding site for ATP in HypCD, protein–ligand docking simulations were performed using CB-Dock2^34,35^ and the crystallographic coordinates of apo-*Tk*HypCD (PDB: 3VYR).^32^ CB-Dock2 performs docking in each cavity of the protein of interest and ranks the cavities according to the best Vina score—an empirical parameter related to the free binding energy. The more negative the score, the more reliable the result. For *Tk*HypCD, CB-Dock2 calculated five different docking sites for ATP (cavities A–E, Figure 3a). Cavity A was found to have the lowest Vina score of −8.3, making it the most favorable binding site for ATP in HypCD. To test the validity of CB-Dock2, we calculated the ATP docking sites in the hydrogenase maturase HypE, which has been shown to bind and hydrolyze ATP for CN^–^ ligand synthesis.^6^

In fact, the ATP-HypE docking data with the lowest Vina score of −8.7 corresponds to the experimentally determined binding site of the nucleotide in HypE^36^ (Figure S8a,b and Table S4). We also calculated the electrostatic charge distribution in *Tk*HypCD at pH 7.0. The surface corresponding to cavity A is strongly positively charged, making it prone to the binding of negatively charged molecules such as ATP (Figure S9). Besides determining various docking locations, CB-Dock2 also discloses the amino acid side chains surrounding the docked ligand (Table S5). For cavity A, amino acid (aa) residues of both HypC and HypD are listed as the connecting residues. These include Cys38_HypD_ and Cys66_HypD_ (*Tk* numbering, Figure 3b), which have been shown to be crucial for the ATPase activity for HypCD and HybG-HypD as well as for the maturation of the hydrogenases I–III from *E. coli*.^33^ The nearby residues also include the GFETT motif (residues 148–152_HypD_), which is highly conserved in HypDs from different species (Figures S10 and S11). When comparing the HypCD docking data for ATP (Figure 3) and the DFT model for the Fe(CN)_2_CO fragment (Figure 2a), it appears that the same protein cleft accommodates both cofactors. In fact, Cys38 of *Tk*HypCD (analogous to Cys41 in *Ec*HypCD) is predicted to be located at hydrogen-bonding distance (ca. 3 Å) from ATP (Figure 3b), and its thiolate side group is proposed to be one of the two protein ligands of the Fe(CN)_2_CO fragment.^31,32,37^

### HypCD Uses ATP to Transfer the Fe(CN)_2_CO Synthon to the Apo-Hydrogenase

**Figure 4:**
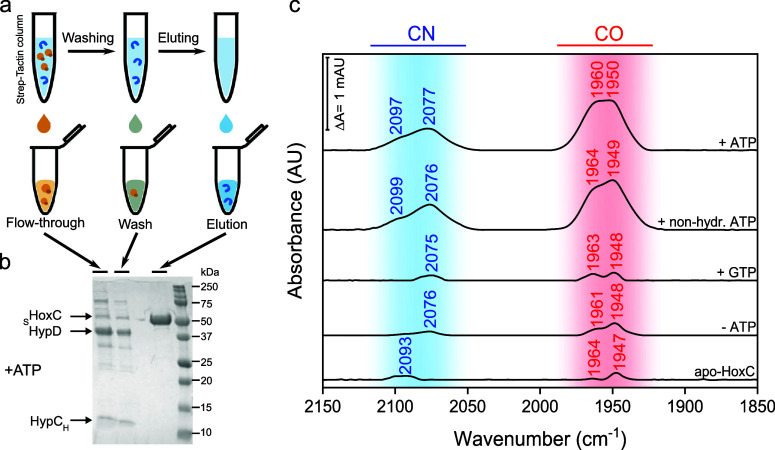
*In vitro* transfer assays using holo-HypCD and apo-HoxC proteins. (a) Schematic representation of the Fe(CN)_2_CO transfer assay using holo-HypC_H_-D (orange) and apo-_S_HoxC (blue). (b) SDS-PAGE analysis of purified _S_HoxC after the transfer reaction in the presence of ATP (see Figure S16 for SDS-PAGE analysis for the transfer reactions with other nucleotides). (c) IR spectra of purified _S_HoxC proteins after the transfer reaction with different nucleotides. CO and CN^–^ absorptions in panel c are labeled with the corresponding wavenumbers. The spectra are normalized with respect to the amide II band intensity. Three independent experiments were performed for each transfer assay, of which one representative IR spectrum (panel c) and one SDS-PAGE (panel b and Figure S16) are shown.

As outlined above, CB-Dock2 data predicted a docking site for ATP in *Tk*HypCD in proximity to residues proposed to bind the Fe(CN)_2_CO fragment. Considering that both apo- and holo-HypCD from *E. coli* have been shown to hydrolyze ATP,^17,18^ it has been hypothesized that ATP might play a role either in the assembly of the Fe(CN)_2_CO fragment (e.g., synthesis of the CO ligand)^22^ or in the transfer of the Fe(CN)_2_CO moiety to the apo-form of the large hydrogenase subunit. Our HypCD preparations harbor the Fe(CN)_2_CO fragment in stoichiometric quantities and can hydrolyze ATP as shown by a biochemical (coupled) assay using pyruvate kinase (PK) and lactate dehydrogenase (LDH) and IR spectroscopy (Supplementary Note and Figures S12 and S13a,b). To investigate whether ATP plays a role in the transfer of the Fe(CN)_2_CO fragment, we designed an *in vitro* assay using holo-HypCD and the apo-form of the large subunit HoxC of the regulatory hydrogenase from *C. necator*. First, we genetically replaced the C-terminal Strep-tag II at HypC in holo-HypCD with a hexa-His affinity tag (Table S2), resulting in holo-HypC_H_-D. UV–vis and IR measurements (Figures S14 and S15) revealed almost identical spectra for holo-HypC_H_-D and holo-HypC_S_-D indicating that the former is fully equipped with a [4Fe–4S] cluster and the Fe(CN)_2_CO synthon. We then incubated holo-HypC_H_-D, ATP (and other nucleotides, see Table S6), and apo-_S_HoxC (Strep-tag II N-terminally fused to HoxC) under anoxic conditions as described in the Methods. After incubation for 1 h, _S_HoxC was repurified by Strep-Tactin affinity chromatography (Figure 4a,b, and Figure S16) and subsequently subjected to IR spectroscopy to verify that the Fe(CN)_2_CO fragment was transferred to the apo-large subunit. The results are summarized in (Figure 4c). ATP turned out to be essential for the transfer of the Fe(CN)_2_CO synthon. Prominent IR absorption bands were observed in HoxC when ATP was present in the reaction mixture (+ATP), while negligible CO/CN^–^-related signals were observed when ATP was omitted (−ATP).

Such minor absorption bands were also observed for highly concentrated apo-HoxC protein solutions and are likely due to the endogenous Hyp machinery in the *E. coli* Rosetta (DE3) strain used to produce apo-HoxC. Although these data may suggest that hydrolysis of ATP is necessary for the transfer of the Fe(CN)_2_CO fragment to the large subunit, this does not appear to be the case, as the non-hydrolyzable ATP analogue β,γ-methyleneadenosine 5′-triphosphate (non-hydr. ATP) also enables efficient cofactor transfer (Figure 4c). An additional control experiment with GTP (instead of ATP), although slowly hydrolyzed by HypCD (Figure S13c), also resulted in negligible CO/CN^–^-related absorption bands in _S_HoxC, thus confirming the necessity of adenine nucleotides. Since holo-HypCD did not hydrolyze β,γ-methyleneadenosine 5′-triphosphate, as shown by IR spectroscopy (Figure S13d), we conclude that the binding of ATP, and not its hydrolysis, is required to transfer the Fe(CN)_2_CO fragment. It is important to note that the IR spectrum of the *in vitro* matured HoxC resembles that of preHoxG^ΔNi^ (Figure S17), a maturation intermediate of a [NiFe]-hydrogenase large subunit purified from living cells, which exclusively carries the Fe(CN)_2_CO unit.^15^ This indicates that our *in vitro* assay enables the incorporation of the Fe(CN)_2_CO synthon into apo-HoxC in a native way.

### ATP Triggers Displacements of the C-Terminal Part of HypC

**Figure 5:**
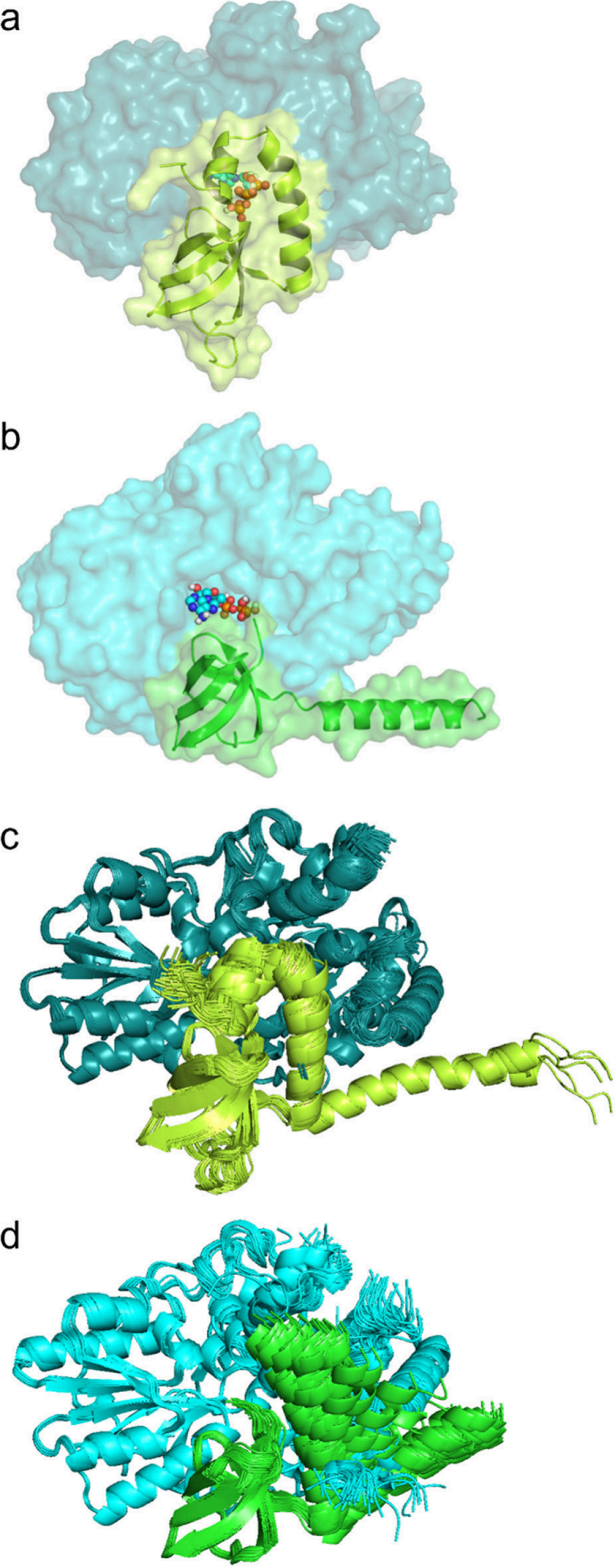
Different conformations of the C-terminal part of HypC in *Ec*HypCD and *Tk*HypCD structure predictions. (a) AlphaFold-predicted “closed conformation” of *Ec*HypCD. The C-terminal part of HypC covers the ATP-binding pocket (ATP is shown in sphere representation). (b) “Open conformation” of the *Tk*HypCD crystal structure. The C-terminal, mostly helical part of HypC bends away from the HypCD complex, exposing the ATP/Fe(CN)_2_CO-binding pocket. HypD is shown in panels a and b in the surface representation (shades of blue), while the different conformations of HypC (shades of green) are displayed in the cartoon representation for clarity. (c) Overlay representation of various structures predicted by AlphaFold 2 for *Ec*HypCD using different presettings. (d) Overlay representation of various structures predicted by AlphaFold for *Tk*HypCD using different presettings. Note that the C-terminal part of HypC can adopt different positions, including the “open” and the “closed”, while the rest of the HypCD complexes remain almost unchanged.

In this study, we employed the holo-HypCD complex from *E. coli* for the *in vitro* transfer experiments. Considering the lack of an atomic structure, we used AlphaFold 2 to predict the *Ec*HypCD complex (Figure 5a and Figure S18). A comparison of the predicted *Ec*HypCD with the X-ray crystal structure of *Tk*HypCD (PDB: 3VYR)^32^ is shown in Figure 5a,b. The corresponding coordinates are provided as part of the Supporting Information and were also used to compute a model for the binding site of the Fe(CN)_2_CO fragment (Figure 2a). Strikingly, a primary docking site for ATP was calculated for *Ec*HypCD (Figure S19 and Tables S7 and S8) that was almost identical (including the Vina score) to that of *Tk*HypCD (Figure S20 and Tables S9 and S10). It is important to note, however, that ATP only docks in cavity A of *Ec*HypCD if the first three N-terminal amino acids Met-Cys-Ile of HypC (Met1 is cleaved by methionine aminopeptidase) were omitted from the docking calculations (Figure S19 and Table S8). These results are in line with the docking calculations performed on the experimentally determined coordinates of *Tk*HypCD (PDB: 3VYR), in which the position of Cys2 and Ile3 remained unresolved (Figure 3, Table S5). Consistently, docking calculations using an AlphaFold-predicted *Tk*HypCD complex, which contained the Cys2-Ile3 dipeptide, confirmed that ATP cannot dock to cavity A (Figure S20, Table S9). Based on these observations, we hypothesized that both the C-terminal part of HypC must be in the open conformation and the outermost N terminus of HypC must change its conformation to allow the binding of ATP in cavity A. In addition, the consistent AlphaFold 2 structure predictions and CB-Dock2 docking data for different HypCD scaffold complexes may allow conclusions to be drawn about HypC dynamics and its functioning.

Since the structural changes are restricted to a small part of the HypCD protein complex, we searched for an experimental method to detect such (dynamic) conformational changes. In recent years, crosslinking mass spectrometry (crosslinking MS) has emerged as key technology in structural biology for investigating protein conformations and protein–protein interactions.^39−42^ Holo- and apo-*Ec*HypC_S_-D preparations with or without ATP were treated with the UV-activatable crosslinker sulfo-SDA, which introduces specific distance constraints into proteins with an upper limit (Cα–Cα) between 25 and 30 Å, for subsequent crosslinked residue pairs (using a 1% link-level false discovery rate). The crosslinked protein samples were subjected to trypsin digestion, and the resulting (crosslinked) peptide mixtures were then analyzed using MS as described in the Methods section.

**Figure 6:**
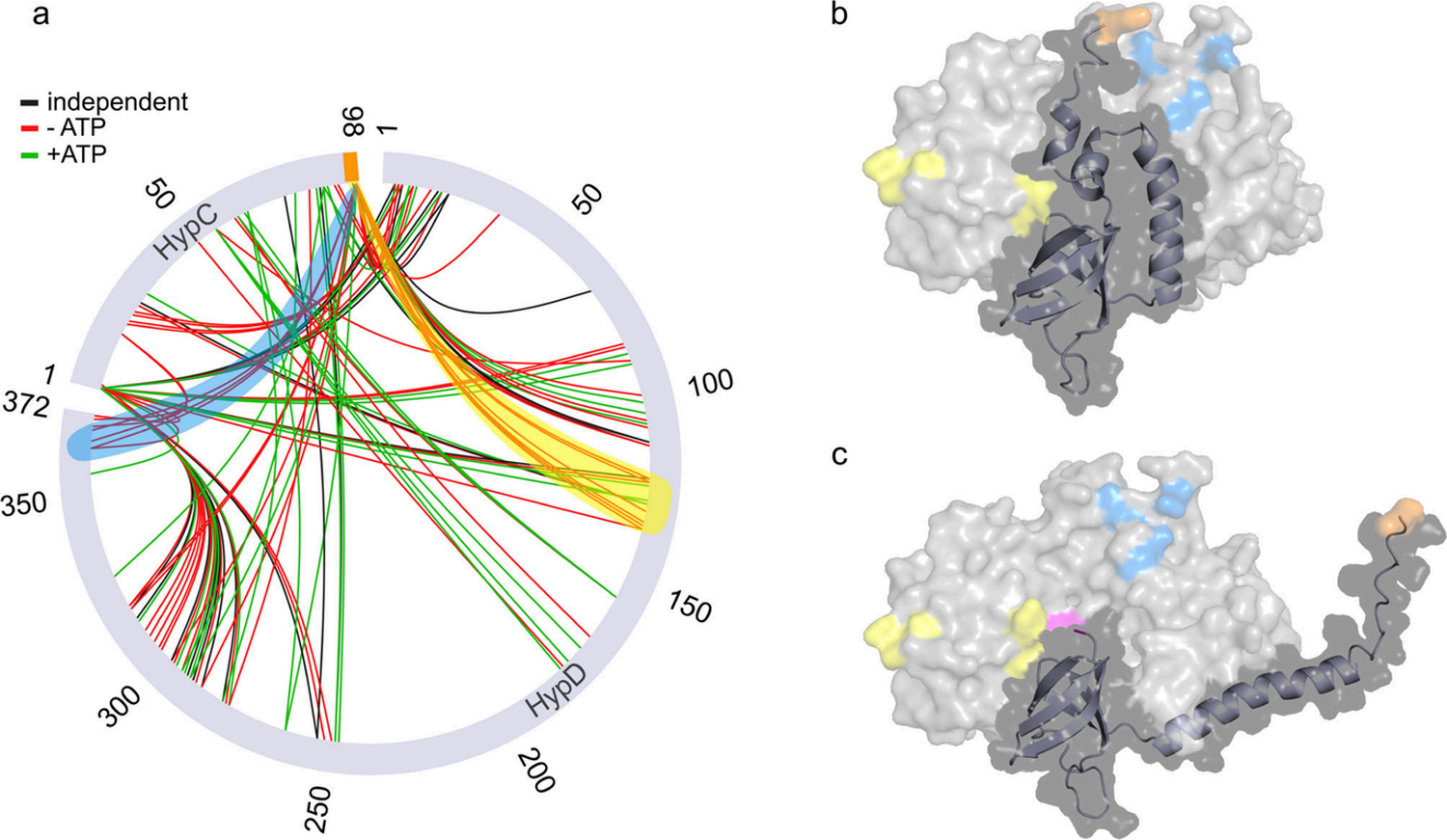
Crosslinking MS analysis of holo-HypC_S_-D with and without ATP. (a) Circular view of the mass spectrometry analysis of the crosslinking within the holo-*Ec*HypC_S_-D complex with or without ATP. HypC and HypD sequences are represented as gray, scaled bars and labeled accordingly. Amino acid numbering is given on the outside of the circle. Crosslinks between the two proteins are depicted as red (−ATP) and green (+ATP) lines. Crosslinks observed in both samples are depicted as black lines (independent). Crosslinks that support a closed conformation of the C-terminal part of HypC (panel b) are highlighted in transparent yellow and blue. The yellow area includes the residues K98 to Y122 as well as S123, A131, N134, P135, and T136, and the blue area includes the residues K98 to Y363 as well as Y365 and Q368. The distances between the amino acids of these crosslinks (aa to aa) were determined with PyMol and are listed in Table 1. Crosslinks in regions of the protein complex remote from the predicted ATP binding site are not discussed. Panels b and c show the AlphaFold 2-predicted *Ec*HypC–D structures in closed and open conformations, respectively, which contain the Strep-tag II peptide sequence at the C terminus of HypC. K98_HypC_ is colored in orange and C2_HypC_ in magenta. The colors of other highlighted residues correspond to the color code used in panel a. Note that C2_HypC_ in the closed conformation is covered by the C-terminal part of HypC and therefore not visible.

The crosslinking MS data obtained for holo-HypC_S_-D (±ATP) are shown in Figure 6. Significantly, we indeed observed crosslinks indicating the existence of both an open and a closed conformation of the C-terminal part of HypC in the HypC_S_-D complex. The crosslinks supporting that the C-terminal part of HypC is positioned in the closed conformation in holo-HypCD (−ATP) include that of residue K98, belonging to the Strep-tag II sequence located at the C terminus of HypC, with HypD residues Y363, Q368, and Y365 (Figure 6a, blue area) with an estimated distance of 22.5, 21.6, and 19.2 Å, respectively, according to the corresponding AlphaFold 2 model (Figure 6b, Table 1). In contrast, in the HypC_S_-D model, where HypC is in the open conformation (Figure 6c), the estimated distances between the above-mentioned amino acid residues exceed the maximum expected length of 25–30 Å (Table 1) for chemical crosslinking.^43^ Other diagnostic crosslinks are those between K98 of HypC_S_ and the HypD residues Y122, S123, A131, N134, P135, and T136 (Figure 6a, yellow area). Here the distances span between 26 and 39 Å for the closed conformation and between 62 and 86 Å for the open conformation (Table 1).

**Table 1:**
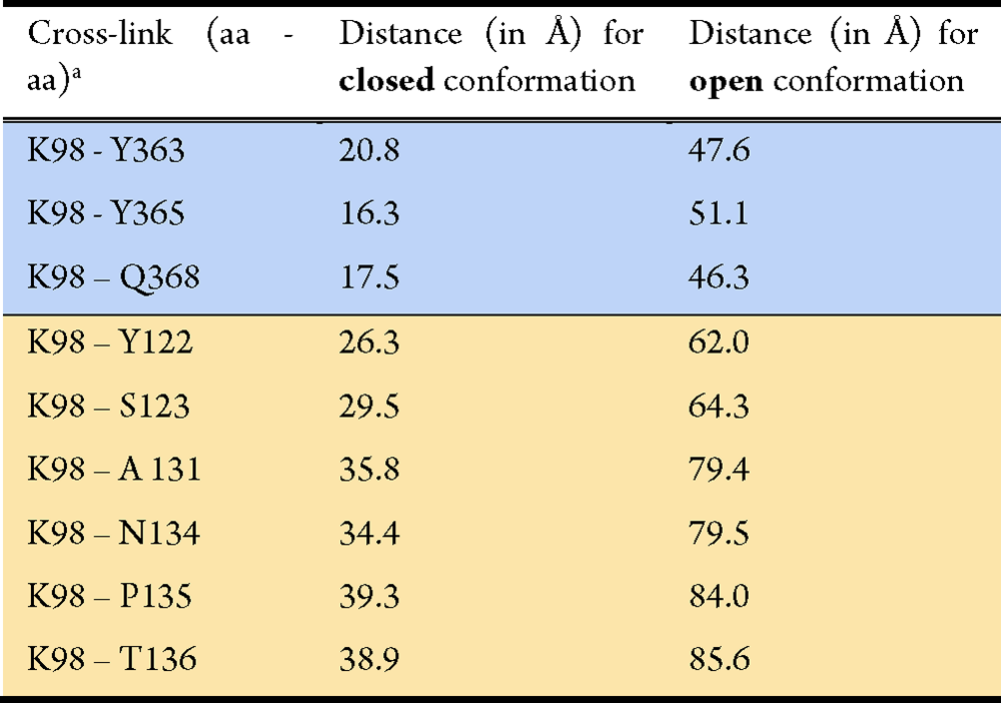
Amino Acid Distances of Selected Crosslinks in Holo-HypCD That Indicate an Open or a Closed Conformation of the C-Terminal Part of HypC

a Blue and yellow colors correspond to those in Figure 6.

Taking into account the flexibility of the Strep-tag II, which is not confidently predicted by the AlphaFold 2 model (Figure 6a and Figure S18) as well as the larger distances for K98 of HypC_S_ and the residues of HypD in the open conformation that exceed the maximum distance for crosslinks, the MS data support the closed conformation of the C-terminal part of HypC_S_ in holo-HypC_S_-D (−ATP). Consistently, the above-mentioned crosslinks were not detected in the holo-HypC_S_-D incubated with ATP, suggesting that the binding of the nucleotide causes the observed (and predicted) conformational changes in HypCD (Figure 6a). Moreover, the ATP-induced changes described above were not observed for apo-HypC_S_-D (Figure S22), implying that the closed conformation of the C-terminal part is induced only in the absence of ATP when the protein is loaded with the Fe(CN)_2_CO cofactor.

Our *in vitro* assays demonstrated that *Ec*HypCD utilizes ATP to deliver the Fe(CN)_2_CO fragment to the apo-form of the hydrogenase large subunit. The combination of the AlphaFold 2 and crosslinking MS data indicates that this may occur through ATP-dependent conformational changes in HypCD, mainly at the C-terminal part of HypC, which suggests a competition-controlled mechanism of transfer for the Fe(CN)_2_CO fragment. As ATP and the Fe(CN)_2_CO fragment cannot bind simultaneously to the same protein pocket due to steric hindrance (both cofactors are predicted to be located at close distance from Cys41 in HypD), we propose that the binding of ATP triggers the dissociation of the Fe(CN)_2_CO fragment from Cys41 in HypD. Consequently, the Fe(CN)_2_CO synthon would remain bound to Cys2 of HypC. This scenario is consistent with a recent report by Gary Sawers’ group showing that the HybG maturase (a HypC paralog) dissociated from HypD carries the Fe(CN)_2_CO group, as evidenced by tandem MS/MS experiments.^16^ HypC/HybG loaded with Fe(CN)_2_CO would then deliver the iron fragment to the apo-form of the hydrogenase large subunit.

**Figure 7:**
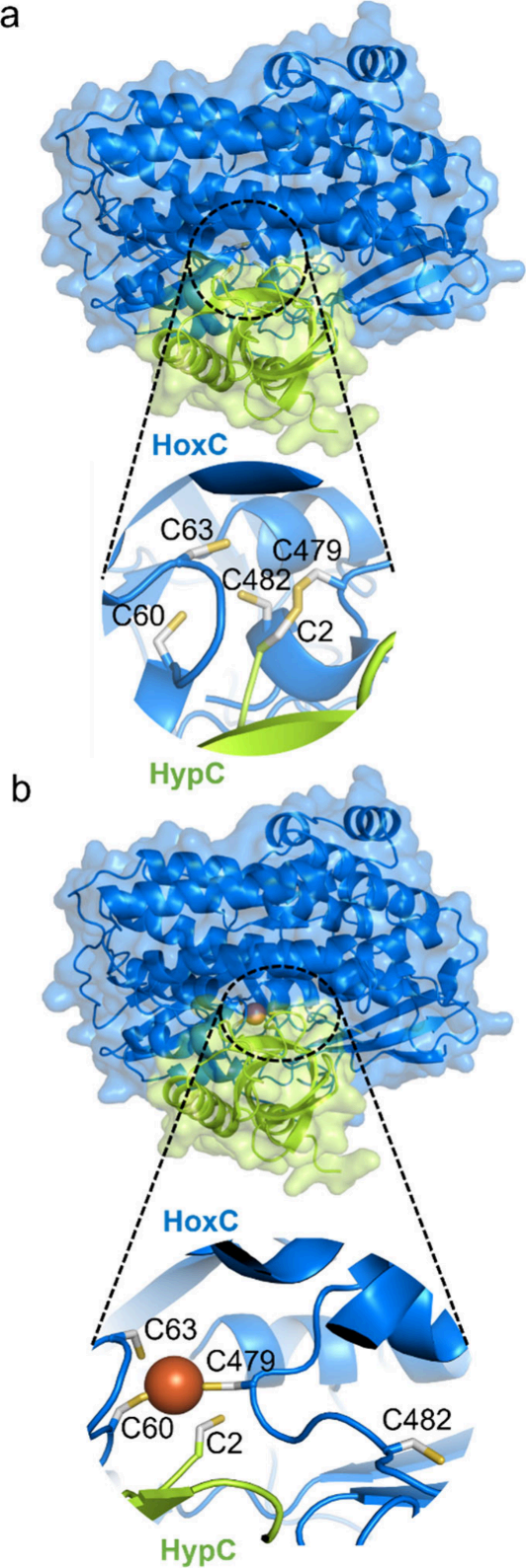
AlphaFold 3-predicted complexes between *Ec*HypC and *Cn*HoxC. (a) Cys2 of HypC protrudes into the [NiFe]-binding motif of HoxC, comprising the residues Cys60, 63, 479, and 482. (b) The predicted HypC–HoxC complex calculated in the presence of an Fe ion exhibits structural changes involving Cys482 of HoxC, which undergoes large displacements as compared to the predicted model in panel a. Note that Cys2 of HypC is still close to the [NiFe]-binding site. HoxC (blue) and HypC (green) are shown in surface and cartoon representations, and the [NiFe]-binding site of HoxC is shown in ball and stick representation with S in yellow, C in gray, and Fe in brown.

To structurally elucidate this interaction, we used AlphaFold 2 to compute a HypC–HoxC protein complex. The model predicts possible interactions between the two proteins, whereby Cys2 of HypC points toward the open cavity of the [NiFe]-binding site of HoxC (Figure S22). However, the low predicted aligned error (PAE) values for the HypC–HoxC complex (Figure S18) indicate that the model is not highly reliable for the relative position of the two proteins to each other and, consequently, also for the position of the N terminus of HypC. By using the recently released AlphaFold 3 server, which allows structural predictions of protein complexes with nucleic acids, small molecules, and even metal ions,^44^ a stable and highly reliable HypC–HoxC complex was predicted (Figure 7a and Figure S23 for PAE and pTM scores), in which the Cys2 of HypC protrudes into the [NiFe]-binding site of HoxC. Interestingly, the presence of an Fe ion in the active center (Figure 7b), which mimics the assembled Fe(CN)_2_CO fragment, induces a displacement of Cys482 of HoxC, which now strikingly adopts an arrangement reminiscent of the structure of the apo-large subunit HyhL from *T. kodakarensis* in complex with the Ni-chaperone HypA.^45^ Importantly, both the HypCD (Figure S24) and HypCDE (Figure S25) complexes show no significant interactions with the large hydrogenase subunit according to the corresponding AlphaFold 2 models, which strengthens the hypothesis that the HypC protein alone delivers the Fe(CN)_2_CO unit to the apo-form of the hydrogenase large subunit.

## Conclusions

In this study, we investigated a key step in the assembly of the [NiFe]-hydrogenase active site, which is the HypCD-mediated incorporation of the low spin Fe^II^(CN)_2_CO synthon into the apo-hydrogenase large subunit. Based on our biochemical, spectroscopic, and computational data, we draw the following conclusions about the role of the HypCD scaffold complex.

**Figure 8:**
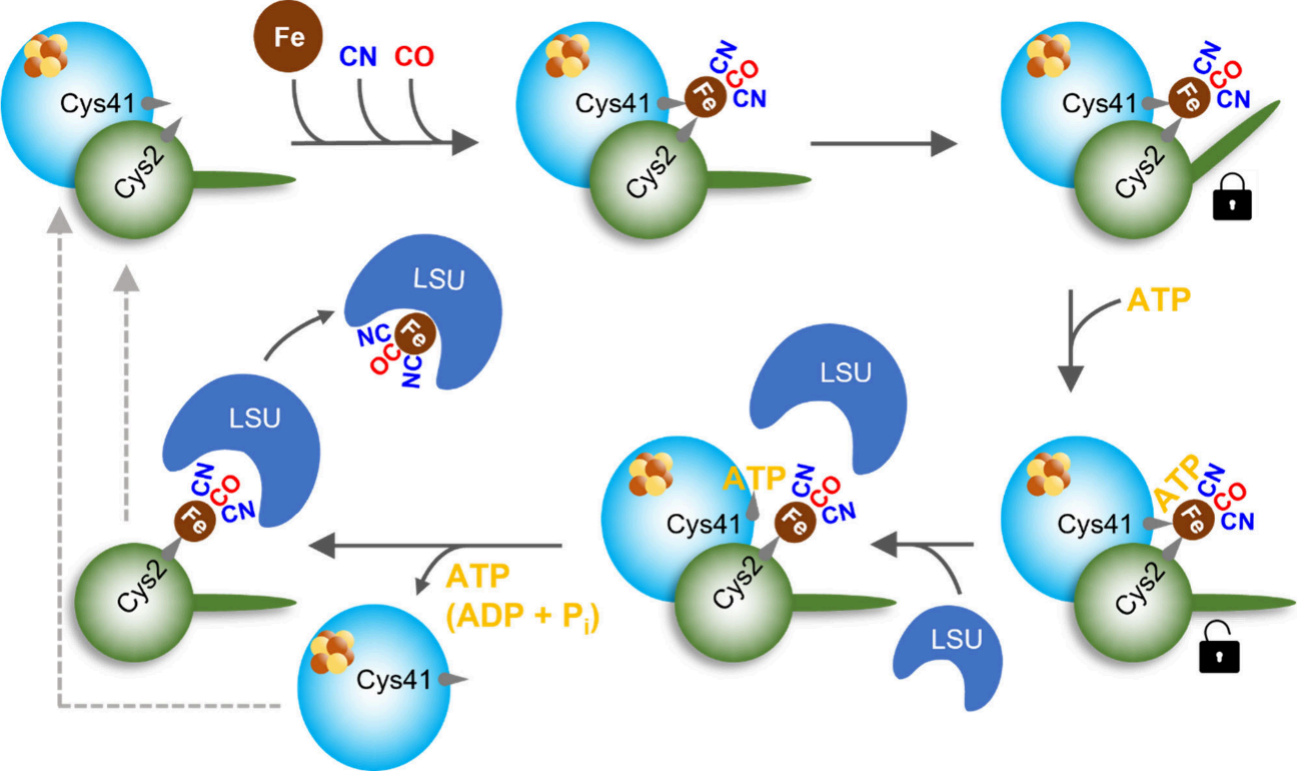
Scheme for the transfer of the Fe(CN)_2_CO fragment from HypCD to the apo-form of the hydrogenase large subunit. The [4Fe–4S] cluster-containing HypD protein is depicted in cyan, and HypC is in green. The C-terminal part of HypC remains in the open conformation until the Fe(CN)_2_CO synthon coordinated by Cys41_HypD_ and Cys2_HypC_ (numbering according to HypCD from *E. coli*) is fully assembled. Our data indicate that the C-terminal part of HypC in holo-HypCD adopts the closed conformation that protects the Fe(CN)_2_CO fragment until further use. Subsequent ATP binding first causes the HypC protein to adopt the open conformation and second, presumably in the presence of the large subunit (LSU), leads to the disruption of the S–Fe(CN)_2_CO bond with Cys41 of HypD and the concomitant rearrangement of the N-terminal Cys2 of HypC with the bound Fe(CN)_2_CO fragment. These structural changes are thought to destabilize the HypCD complex, thereby releasing ATP (or its hydrolysis products) and Fe(CN)_2_CO-loaded HypC, which is then proposed to form a transient complex with LSU to deliver the Fe(CN)_2_CO fragment (Figure 7). The released HypC and HypD proteins can then enter another cycle (dashed lines).

(1) Holo-HypCD houses two Fe-containing cofactors, a [4Fe–4S] cluster, and a low-spin Fe^II^ species with a 5-fold coordination comprising one CO, two CN^–^, and two cysteine thiolates (Cys2_HypC_ and Cys41_HypD_, *E. coli* numbering) as ligands. (2) Binding of ATP, not its hydrolysis, is required for transfer of the Fe(CN)_2_CO moiety to the apo-form of the hydrogenase large subunit. Protein–ligand docking data identified an ATP binding site in a HypD cavity at the HypC–HypD interface. (3) The binding of ATP presumably takes place in the cavity that also accommodates the Fe(CN)_2_CO synthon, and among others, the synthon-binding Cys41 of HypD is involved in the ATP binding. Considering that Cys2 of HypC and Cys41 of HypD must be close to each other to stably bind the Fe(CN)_2_CO fragment,^32^ we propose that the binding of ATP triggers significant rearrangements in HypCD, resulting in (i) the disruption of the Fe(CN)_2_CO thiolate complex with Cys41 of HypD and (ii) the displacement of the N-terminal Cys2 of HypC, which alone carries the Fe(CN)_2_CO fragment. As this step requires reducing power, the [4Fe–4S] cluster and the nearby thioredoxin (dithiol–disulfide) moiety represent possible electron donors.^22^ (4) AlphaFold 2 structure predictions and crosslinking MS revealed significant ATP-dependent conformational changes of the C-terminal, mostly α-helical part of HypC exclusively in the holo-HypCD complex. An open and a closed conformation were identified that could reliably regulate the binding/dissociation of the ATP/Fe(CN)_2_CO cofactors, as depicted in Figure 8. (5) It has been hypothesized that the HypCD complex dissociates to allow the delivery of the Fe(CN)_2_CO fragment to the apo-form of the hydrogenase large subunit.^16^ Our AlphaFold 3 data allow us to visualize a possible Fe-dependent interaction between the N terminus of HypC and the NiFe-binding site of the large subunit HoxC.

In summary, our results provide new insights into the sophisticated protein machinery responsible for [NiFe]-hydrogenase maturation. The fact that ATP and a non-hydrolyzable ATP analogue both trigger Fe(CN)_2_CO synthon transfer suggests that nucleotide binding alone induces the required structural rearrangements in the HypCD complex. This mechanism is reminiscent of previous observations on multidrug resistance protein MRP1,^46^ RNA strand separation by DEAD-box proteins,^47^ the proteasome-regulatory ATPase PAN,^48^ clamp loading of DNA, which enhances the processivity of DNA polymerase,^49^ and the molecular chaperone HSP90.^50^ In all of these cases, ATP binding alone leads to structural changes important for initial functions, while ATP hydrolysis is required for later processes. In this sense, the ATP-hydrolyzing activity of HypCD might be important for the release of the nucleotide after synthon transfer, thereby priming the HypCD complex for another cycle (Figure 8). This could not be tested in our *in vitro* assay because it is inherently suitable for only a single transfer cycle. Therefore, we cannot exclude other roles for ATPase activity, e.g., in the synthesis or binding of the CN^–^ and CO ligands to the iron ion in HypCD. However, our results emphasize the importance of NTPases for the assembly of metallocofactors^51^ and point to the essential role of ATP in the displacement of the Fe(CN)_2_CO synthon in the HypCD complex. This, in addition to the catalytic reactions of the maturases HypE and HypF,^6^ is the third ATP-dependent function of [NiFe]-hydrogenase maturation.

## Methods

### Bacterial Strains, Cultivation Conditions, and Protein Purification

For heterologous protein production, the *E. coli* strain Rosetta (DE3) was used. The corresponding expression plasmids are listed in Table S2, and their construction is described in Supplementary Methods (Table S11). Strains were grown aerobically in LB medium containing 100 μg/mL of carbenicillin and 34 μg/mL of chloramphenicol. Precultures were grown overnight at 37 °C and used to inoculate (1:100) 1 L main cultures, which were subsequently cultivated at 37 °C and 120 rpm until an OD_600_ between 0.4 and 0.8 was achieved. Gene expression was induced by adding 0.5 mM isopropyl beta-d-1-thiogalactopyranoside (IPTG). The cells were incubated overnight at 16 °C and 120 rpm and finally harvested by centrifugation at 11,500 × *g* for 15 min at 4 °C. Cell pellets were either processed immediately or frozen in liquid nitrogen and stored at −80 °C. All purification steps were performed aerobically at 4 °C. A 1 g sample of cell pellet was resuspended in 3 mL of the corresponding buffer (Table S1). Cell lysis was achieved in a French pressure cell with a pressure of 125 MPa. After ultracentrifugation at 100,000 × *g* for 45 min at 4 °C, the soluble extract was loaded onto either a Strep-Tactin Superflow resin (high-capacity, IBA Lifesciences) column or a HisPur Ni-NTA resin (Thermo Scientific) column, depending on the affinity tag. The washing and elution procedures as well as the buffer compositions are listed in Table S1. Eluted proteins were first concentrated (Ultracel Amicon Ultra 15 mL centrifugal filter units), followed by a 10-fold dilution with washing buffer, and then concentrated again by ultrafiltration. This process was repeated four times to wash away residual imidazole or d-desthiobiotin. Finally, protein aliquots were frozen and stored in liquid nitrogen. Concentration was determined via the Pierce BCA protein assay kit (Thermo Scientific).

Production of ^57^Fe-labeled HypCD proteins was conducted as follows. When HypCD-overproducing cell cultures reached an OD_600_ between 0.4 and 0.8, 2.5 mL of ^57^FeCl_2_ solution (20.5 mM in 1.5 M HCl) was added to achieve a final concentration of ca. 50 μM. Cells were first incubated for ∼20 min at room temperature (RT), and gene expression was then induced as described above. All purification steps were identical with those used to isolate HypCD from media containing iron with natural isotope distribution.

### Polyacrylamide Gel Electrophoresis and Metal Determination

Protein purity was verified by SDS-PAGE. 3.5 μg of purified proteins was separated on a 15% acrylamide separating gel at 0.04 A for ca. 45 min. Protein bands were visualized by Coomassie staining. The metal content of the purified HypCD samples was determined via inductively coupled plasma optical emission spectrometry (ICP-OES). Three technical replicates of a 10 μM protein solution (each 500 μL) were mixed with 500 μL of nitric acid (65% v/v). After being incinerated at 100 °C overnight, samples were filled to 5 mL with ultrapure water and measured in an Optima 2100DV ICP-OES (PerkinElmer). The same procedure was repeated for three technical replicates of the buffer solution.

### ATPase Activity of HypCD

ATPase activity was determined biochemically using a coupled enzymatic assay comprising a pyruvate kinase (PK) and a lactate dehydrogenase (LDH) from rabbit muscle (Sigma).^52^ In a first reaction, ATP is hydrolyzed by HypCD to ADP and phosphate. The produced ADP and phosphoenolpyruvate (PEP) are then used by PK to form pyruvate and ATP. Last, pyruvate is consumed by LDH to form lactate. During this last assay reaction, NADH is oxidized to NAD^+^, which is detected by UV–vis spectroscopy due to the decrease in absorbance at 340 nm (ε = 6.3 mM^–1^ cm^–1^).^53^ Each reaction was conducted at 25 °C in buffer containing 100 mM Tris/HCl, pH 7.5, 2.5 mM MgCl_2_, 1 mM PEP, 1 mM ATP, and 0.3 mM NADH. The final amount of PK and LDH was 4 and 3 U, respectively. To avoid an unspecific increase of absorbance due to residual ADP in the reaction mixture, HypCD was added when the absorbance of the solution reached a plateau. To verify that HypCD was exclusively responsible for ATP hydrolysis, we also recorded IR spectra of ATP with and without HypCD by monitoring the absorbances of the reactant and the hydrolysis products (Figure S13). In this case, HypCD was incubated in an anaerobic workstation with ATP, NaDT, and MgCl_2_ in a molar excess of 40-fold, 30-fold, and 20-fold, respectively, and the mixture was then transferred to the IR cell.

### Spectroscopy

#### Infrared

HypCD and HoxC protein solutions were transferred into a homemade, gastight, and temperature-controlled transmission cell equipped with two sandwiched CaF_2_ windows separated by a Teflon spacer (optical path length of 50 μm). Spectra with a resolution of 2 cm^–1^ were recorded at 10 °C using a Bruker Tensor 27 Fourier-Transform spectrometer equipped with a liquid-nitrogen-cooled mercury cadmium telluride detector. The sample compartment was purged with dried air. For a single spectrum 200 individual scans were averaged. A buffer spectrum was used as a reference to calculate the corresponding absorbance spectra. OPUS software version 7.5 (Bruker) was used for data analysis.

#### Mössbauer

Zero-field Mössbauer spectra of ^57^Fe-labeled HypCD apo- and holo-samples at a concentration of ca. 1.5 mM were acquired on a SEECO MS6 spectrometer comprising the following instruments: a JANIS CCS-850 cryostat, including a CTI-CRYOGENICS closed cycle 10 K refrigerator, and a CTI-CRYOGENICS 8200 helium compressor. The cold head and sample mount were equipped with calibrated DT-670-Cu-1.4L silicon diode temperature probes and heaters. The temperature was controlled by a LAKESHORE 335 temperature controller. Spectra were recorded using an LND-45431 Kr gas proportional counter with a beryllium window connected to the SEECO W204 γ-ray spectrometer that includes a high voltage supply, a 10 bit and 5 μs ADC, and two single channel analyzers. Motor control and recording of spectra were taken care of by the W304 resonant γ-ray spectrometer. For the reported spectra, a RIVERTEC MCO7.114 source (^57^Co in Rh matrix) with an activity of about 1 GBq was used. All spectra were recorded on frozen solutions at 13 K, and data were accumulated for about 24–72 h. Mössbauer data were processed and simulated using the WMOSS4 program (www.wmoss.org). Isomeric shifts are referenced to α-iron at room temperature.

#### NRVS

NRVS measurements were conducted at a Petra III P01 (Germany) using 14.41 keV radiation (^57^Fe). Raw NRVS data were converted to single-phonon ^57^Fe partial vibrational densities of states (PVDOS) using the PHOENIX software package via the “NRVS Spectra Processing Tool” web interface (https://www.spectra.tools/). The energy scales were calibrated with a [NEt_4_][^57^FeCl_4_] sample characterized by two prominent peaks at 378 cm^–1^ (asymmetric Fe–Cl stretching mode) and 139 cm^–1^ (Fe–Cl bending mode).^54^ The temperature of the samples was maintained at ca. 12 K by using a liquid He cryostat. The real sample temperature, as obtained from the spectral analysis, was 40–50 K. To enhance the signal-to-noise ratio in the Fe–CO/CN spectral range, sectional measurements were performed. Each scan was divided into two segments with different data collection times (second per point, s/pt). We used 3–4 s/pt for the region from −80 to 360 cm^–1^ and 9–10 s/pt for the region from 360 to 700 cm^–1^.

#### UV–Visible

UV–vis spectra were recorded using a Cary50 UV–vis spectrophotometer (Varian, Agilent, USA) at room temperature. Each protein was used at a concentration of 40 μM. A buffer spectrum was used as a reference.

#### Crosslinking Mass Spectrometry

Apo-HypC_S_-D was purified by affinity chromatography, while holo-HypC_S_-D was additionally subjected to size-exclusion chromatography on a Superdex 200 Increase 10/300 GL (Cytiva) to remove unwanted oligomers (ternary HypCDE complex and HypC dimers). Purified HypCD complex (20 μg, 0.5 mg/mL) was crosslinked using sulfo-SDA (sulfosuccinimidyl 4,4′-azipentanoate (Thermo Scientific Pierce) at 0.76 mM in a crosslinking buffer (MOPS 50 mM, 150 mM NaCl, 0.1 mM DTT, pH 7.5). For the HypCD plus ATP sample, ATP was added to purified HypCD (9 mM final crosslinking reaction concentration) 10 min prior to addition of crosslinker. The crosslinking reaction involved two steps: 1. Crosslinker and protein were incubated for 30 min at room temperature in the dark; 2. The sample was irradiated with high-power UV light (LuxiGen 365 nm UV LED Gen 4 Emitter, LED Engine, operated at 1000 mA) for 20 s. After crosslinking, protein was acetone-precipitated and digested using an adapted SPEED protocol.^55^ Briefly, pelleted proteins were dissolved in 10 μL of trifluoracetic acid (TFA), neutralized by addition of 100 μL of 2 M Tris base, and reduced and alkylated with the addition of 11 μL of TCEP/2-chloroacetamide (at final concentrations of 10 and 40 mM, respectively) and heating for 5 min at 95 °C. Protein samples were diluted 1:5 with water, and digestion was carried out for 20 h at 37 °C using trypsin at an enzyme:protein ratio of 1:50. Digests were acidified with TFA and desalted using C18 StageTips.^56^

LC-MS/MS analysis was performed on a Q Exactive High-Field (HF) Hybrid mass spectrometer (Thermo Fisher Scientific) coupled to an Ultimate 3000 UHPLC system (Dionex, Thermo Fisher Scientific). Peptides were separated on a 50 cm EASY-Spray column (Thermo Fisher). Mobile phase A consisted of 0.1% (v/v) formic acid and mobile phase B of 80% (v/v) acetonitrile with 0.1% (v/v) formic acid. The following LC gradient was applied with a flow rate of 300 nL/min: 2% B to 11% B in 10 min, to 35% B in 77 min, to 50% B in 5.5 min, ramping to 95% B in 2.5 min, and holding for 5 min (total run time, 120 min). Mass spec data were acquired in data-dependent mode, with both MS1 and MS2 scans carried out in the Orbitrap. Full scan mass spectra were recorded in the range of 400–1450 *m*/*z* at a resolution of 120,000 (AGC target, 1e6; injection time of 50 ms). The ten most intense ions in the full scan, with precursor charge states between 3+ and 6+, were isolated with a *m*/*z* window of 1.4 Th and fragmented using higher-energy collision-induced dissociation (HCD) at stepped normalized collision energies of 20, 28, and 30%. Subsequent fragmentation spectra were recorded at a resolution of 60,000, using an AGC target of 2e5 and maximum injection time set to 250 ms. Dynamic exclusion (single repeat count) was set to 30 s. Precursors *m*/*z* were recalibrated based on linear peptide identifications at <1% False Discovery Rate (FDR). Recalibrated peak lists were searched for crosslink identification against the sequences of HypC and HypD, using the Xi software suite (version 1.7.6.4; https://github.com/Rappsilber-Laboratory/xiSEARCH)^57^ with the following settings: MS1 accuracy, 3 ppm; MS2 accuracy, 5 ppm; enzyme, trypsin, allowing up to 2 missed cleavages and 2 missing monoisotopic peaks; crosslinker, SDA (NHS-ester reaction specificity for lysine, serine, threonine, tyrosine, and protein N-termini); fixed modifications, carbamidomethylation on cysteine (cm, +57.02 Da); variable modifications, methylation on glutamic acid (me, +14.01), oxidation on methionine (ox, +15.99 Da), SDA-loop (+82.04 Da), and SDA-hyd (+100.05). SDA MS-cleavability was considered during the database search. Identified crosslinks were filtered to 1% FDR on link level with xiFDR version 2.2.RC2.^42^

#### 3D Structure Predictions

The AlphaFold 2 models of HypCD complexes from *Thermococcus kodakarensis* (*Tk*) and *Escherichia coli* (*Ec*), the latter with and without the Strep-tag II sequence at the C terminus of HypC, as well as the complex between *Ec*HypC and HoxC from *C. necator* (*Cn*) were calculated using ColabFold v1.5.5.^58,59^ For prediction of conformational variability, the following settings were applied: “max_msa” was set to “32:64”, “num_seeds” was set to “16”, and “use_dropouts” was not activated (“use_dropouts” did not lead to obvious structural changes when activated).^60^ The accession numbers of the protein sequences used for the AlphaFold structure predictions (Supplementary Data I) are listed in Table 2. During preparation of the manuscript, AlphaFold 3 was released^44^ and we used it to compute both the HypCD and HypC–HoxC complexes in the presence of ATP and Fe ions. The interatomic distances listed in Table 1 were determined using PyMOL 2.5.5.^61^

**Table 2:** Accession Numbers of Protein Sequences Used for the AlphaFold Structure Predictions

Protein^a^	Accession number
*Tk*HypCD	3vyr (RCSB)
*Ec*HypC	P0AAM3 (UniProt)
*Ec*HypD	P24192 (UniProt)
*Cn*HoxC	Q79IP6 (UniProt)

a *Tk* - *Thermococcus kodakarensis*, *Ec* - *Escherichia coli*, *Cn* - *Cupriavidus necator*.

#### DFT Model Setup

##### i

Initial coordinates used for the density functional theory (DFT) modeling were constructed based on the () cofactor-free AlphaFold prediction of the *Ec*HypCD protein structure and (*ii*) Fe(CN)_2_(CO)(Cys)_2_ fragment extracted from the PDB 3RGW(62) (X-ray structure of the O_2_-tolerant membrane-bound [NiFe]-hydrogenase from *Cupriavidus necator*). The Fe(CN)_2_(CO)(Cys)_2_ fragment was docked manually into the *Ec*HypCD framework so that one of its cysteine side chains aligns with Cys41_HypD_, and the second cysteine is positioned in the region of Cys2_HypC_. Twelve more *Ec*HypCD residues in the environment of Fe(CN)_2_CO were retained in the DFT model as shown in Figure 2a and Figure S6, overall defining the system as [**C**_**2**_]_HypC_ – Fe(CN)_2_(CO) – [**C**_**41**_G_42_G_43_H_44_/G_68_C_69_*/F_147_/T_149_/P_199_G_200_H_201_/F_227_/E_357_]_HypD_ (metal ligands are in bold). Here, the backbone spacers were included between the consecutive HypD residues. The side chain of Cys69_HypD_ (*) was omitted, equivalent to a C-to-G modification, as it points away from the cofactor. All the C_α_ carbon atoms were fixed to their reference AlphaFold positions during DFT structure optimization (see method details below) as often employed in protein modeling,^63^ except that of Cys2_HypC_, which varies its position (see the Results section). The titratable imidazole groups of His44/201_HypD_ were singly protonated corresponding to the neutral pH. In contrast, Glu357_HypD_ carboxylate was protonated due to its proximity to the Cys2_HypC_ thiolate sulfur (Figure 2a and Figure S6).

#### DFT Methods

Molecular geometry optimization and subsequent Hessian were accomplished using Gaussian 16, revision C.01,^64^ assisted with densities exported from single-point calculations using JAGUAR 11.0.^65^ All calculations employed the PBE0^66^ hybrid functional and the LACV3P** basis set as implemented in JAGUAR. For the first- and second-row elements, LACV3P** implies 6-311G** triple-ζ basis sets including polarization functions. For the Fe center, LACV3P** consists of a triple-ζ basis set for the outermost core and valence orbitals and the quasi-relativistic Los Alamos effective core potential (ECP) for the innermost electrons.^67,68^ Molecular environment was considered using a self-consistent reaction field (SCRF) polarizable continuum model and integral equation formalism^69^ (IEF-PCM) as implemented in Gaussian. The static dielectric constant was set to ε = 4.0, as often used for proteins, and the remaining IEF-PCM parameters were kept at their default values for water. The ^57^Fe-PVDOS and interatomic relative displacement kinetic energy distribution (KED) intensities were extracted from Gaussian normal mode outputs using an in-house program Q-SPECTOR.^70^ To empirically account for the observed NRVS line shape, the computed ^57^Fe-PVDOS intensities were broadened by Lorentzian convolution with a full width at half-maximum (fwhm) of 14 cm^–1^. For ^57^Fe-PVDOS, empirical scaling by ×1.07/1.02/0.97 was applied to the calculated frequencies in the 400–560/560–610/610–660 cm^–1^ regions, respectively.

#### *In Vitro* Transfer Assay

All steps were carried out under anaerobic conditions in an anaerobic workstation with a temperature of about 10 °C. Buffer solutions and protein concentrators (Amicon Ultracel, Millipore, 30 kDa cutoff) were degassed prior to usage and equilibrated with anaerobic buffer. The assay was carried out in a 1.5 mL Eppendorf vial with the following components: 50 mM MOPS/KOH, pH 7.4 at 4 °C, 150 mM NaCl, 15 mM NaDT, 10 mM MgCl_2_, 20 mM of ATP/GTP/non-hydrolyzable ATP analogue (see Table S6 for further details). 0.06 mM apo-HoxC_S_ (0.06 mM) was reacted with 0.3 mM holo-HypC_H_-D protein solution (5 mol equiv). After incubation, the whole assay reaction was loaded on a Strep-Tactin Superflow column (high-capacity, iba Lifescience) with a column volume (CV) of 1 mL, to isolate HoxC_strep_ from the reaction mixture. The column was washed with 10 CVs of washing buffer (50 mM MOPS/KOH, pH 7.4 at 4 °C, 150 mM NaCl), and the matrix-bound protein was eluted with 5 CVs of washing buffer supplemented with 3.0 mM d-desthiobiotin. Elution fractions were concentrated using Ultracel Amicon Ultra 0.5 mL centrifugal filter units (cutoff 30 kDa) followed by a 5-fold dilution step with washing buffer to remove residual desthiobiotin. The washing step was repeated 4 times, followed by a final step of protein concentration (resulting volume of ca. 20 μL) and IR measurements.

##### CB-Dock2 Cavity Detection-Guided Blind Docking

For a protein–ligand blind docking simulation the online tool CB-Dock2 was used.^34,35^ The X-rays coordinates of *T. kodakarensis* HypCD^32^ and the AlphaFold-predicted structures of *E. coli* HypCD (Supplementary Data I) were employed as proteins. As ligand, the coordinates of ATP (DrugBank ID: DB00171) were used. The docking simulation is based on a perl script that processes the submitted files as described at https://cadd.labshare.cn/cb-dock2/php/manual.php. AutoDock-Vina (version 1.2.0) is used for template-independent blind docking, and template-based blind docking uses the BioLip database (version 2021.09.15) as the template database. Output files are given as receptor–ligand PDB files for all detected cavities (Supplementary Data II).

## Data Availability

The authors declare that the data supporting the findings of this study are available within the article and the Supporting Information. Raw NRVS data were generated at the synchrotron facility Petra III, and are available in their processed form upon request. Crosslinking MS data are deposited to the ProteomeXchange Consortium via the PRIDE database with the data set identifier PXD051661 and 10.6019/PXD051661.

## Supporting Information Available

Supplementary Figures S1–S25, supplementary Tables S1–S11, supplementary note on ATPase activity measurements, supplementary methods, and supplementary references (PDF)
AlphaFold structure predictions (Supplementary Data I), output files of CB-Dock2 (Supplementary Data II), and selected DFT normal mode animations (GIF format) relevant to NRVS bands and the optimized structures (PDB format) for the DFT-computed models (Supplementary Data III) (ZIP)

## Abbreviations

DFT: density functional theory
IR: infrared
NRVS: nuclear resonance vibrational spectroscopy
PVDOS: partial vibrational densities of states
UV–vis: ultraviolet/visible
