# Supplementary material for: ATP-Triggered Fe(CN)2CO Synthon Transfer from the Maturase HypCD to the Active Site of Apo-[NiFe]-Hydrogenase
Source: J Am Chem Soc. 2024 Nov 4;146(45):30976–89. doi: 10.1021/jacs.4c09791 (PMC11565642; doi:10.1021/jacs.4c09791)
Supplement: Supplementary file 1 — ja4c09791_si_001.pdf [file ja4c09791_si_001.pdf]

Supporting Information

ATP-Triggered $Fe(CN)_2CO$ Synthon Transfer from the Maturase HypCD to the Active Site of Apo-[NiFe]-Hydrogenase

Anna Kwiatkowski[1], Giorgio Caserta[1*], Anne-Christine Schulz[1], Stefan Frielingsdorf[1], Vladimir Pelmenschikov[1], Kilian Weisser[2], Adam Belsom[3], Juri Rappsilber[3,4,5], Ilya Sergueev[6], Christian Limberg[2], Maria-Andrea Mroginski[1], Ingo Zebger[1] and Oliver Lenz[1*]

[1] Institut für Chemie, Technische Universität Berlin, Straße des 17. Juni 135, 10623 Berlin, Germany

[2] Institute of Chemistry, Humboldt-Universität zu Berlin, Brook-Taylor-Straße 2, 12489 Berlin, Germany

[3] Institute of Biotechnology, Chair of Bioanalytics, Technische Universität Berlin, Gustav-Meyer-Allee 25, 13355 Berlin, Germany

[4] Si-M/'Der Simulierte Mensch', a Science Framework of Technische Universität Berlin and Charité – Universitätsmedizin Berlin, 10623 Berlin, Germany

[5] Wellcome Centre of Cell Biology, University of Edinburgh, Edinburgh EH9 3BF, UK

[6] Deutsches Elektronen-Synchrotron, Notkestraße 85, 22607 Hamburg, Germany

* Corresponding authors

Table of Contents

Figure S1. Schematic overview of the sequential steps of [NiFe]-hydrogenase active site metalation S4

Figure S2. Purified apo-HypC$_S$-D, holo-HypC$_S$-D, holo-HypC$_H$-D and apo-$_S$HoxC separated on an SDS-PAGE gel S5

Figure S3. IR-spectroscopic characterization of purified HoxC and HypCD S6

Figure S4. Purification of HypC and HypD and their characterization by IR spectroscopy S7

Figure S5. Comparison of the NRVS spectra of holo-HypC$_S$-D and the maturation intermediate preHoxG$^{\Delta Ni}$ S8

Figure S6. DFT model of the $Fe(CN)_2CO$ cofactor binding region in *Ec*HypCD S9

Figure S7. ^{57}Fe-PVDOS of *Ec*HypCD from NRVS experiment and DFT modeling S10

Figure S8. ATP docking to HypE from *T. kodakarensis* S11
Figure S9. Electrostatic potential surface analysis of HypCD S12
Figure S10. Multiple Sequence Alignment of HypD from various species S13
Figure S11. Sequence logo derived from the multiple sequence alignment of HypD S15
Figure S12. ATPase activity of the HypCD complex determined by a coupled enzyme assay S16
Figure S13. Nucleotide hydrolysis catalyzed by HypCD observed via IR spectroscopy ... S17
Figure S14. IR spectra of Strep- and His-tagged holo-HypCD S18
Figure S15. UV-vis spectra of holo-HypC$_H$D, holo-HypC$_S$D and apo-HypC$_S$D S18
Figure S16. $_S$HoxC protein samples purified after incubation with HypC$_H$D in the presence or absence of different nucleotides S19
Figure S17. IR spectra of $_S$HoxC preparations isolated after the *in vitro* transfer experiments compared to that of preHoxG$^{\Delta Ni}$ S20
Figure S18. Predicted Aligned Error (PAE) analysis and predicted local distance difference test (pLDDT) from the AlphaFold structural predictions S21
Figure S19. Superimposed AlphaFold 2 structures of *Ec*HypCD with and without N-terminally truncated HypC S24
Figure S20. Superimposed AlphaFold 2 structures of *Tk*HypCD with and without N-terminally truncated HypC S25
Figure S21. Crosslinking MS data on apo-HypC$_S$-D incubated with and without ATP .. S26
Figure S22. AlphaFold 2-predicted complexes between *Ec*HypC and *Cn*HoxC S27
Figure S23. Predicted Aligned Error (PAE) analysis and predicted local distance difference test (pLDDT) for the AlphaFold 3-predicted complexes of *Ec*HypC and *Cn*HoxC S28
Figure S24. AlphaFold 2 structure prediction of *Cn*HoxC and *Ec*HypCD S29
Figure S25. AlphaFold 2 structure prediction of *Cn*HoxC and *Ec*HypCDE S29

Table S1. **Protein purification buffers** ... S31
Table S2. **Plasmids used in this study** ... S32
Table S3. **Mössbauer parameters of apo- and holo-HypC$_S$-D** ... S32
Table S4. **CB-Dock2 analysis of ATP-binding sites in HypE from *T. kodakarensis*** ... S32
Table S5. **CB-Dock2 analysis of ATP-binding sites in *Tk*HypCD** ... S33
Table S6. **Nucleotide solutions used for the *in vitro* transfer assay** ... S33
Table S7. **CB-Dock2 analysis of ATP-binding sites in the AlphaFold 2-predicted *Ec*HypCD structure carrying native HypC** ... S33
Table S8. **CB-Dock analysis of ATP-binding sites in the AlphaFold 2-predicted *Ec*HypCD structure carrying an N-terminally truncated version of HypC** ... S34
Table S9. **CB-Dock analysis of ATP-binding sites in the AlphaFold 2-predicted *Tk*HypCD structure carrying native HypC** ... S34
Table S10. **Values from CB-Dock analysis using the AlphaFold 2-predicted *Tk*HypCD carrying an N-terminally truncated version of HypC and ATP** ... S35
Table S11. **Primers used in this study** ... S35

Supplementary Note ... S16
Supplementary Methods ... S30
Supplementary References ... S36

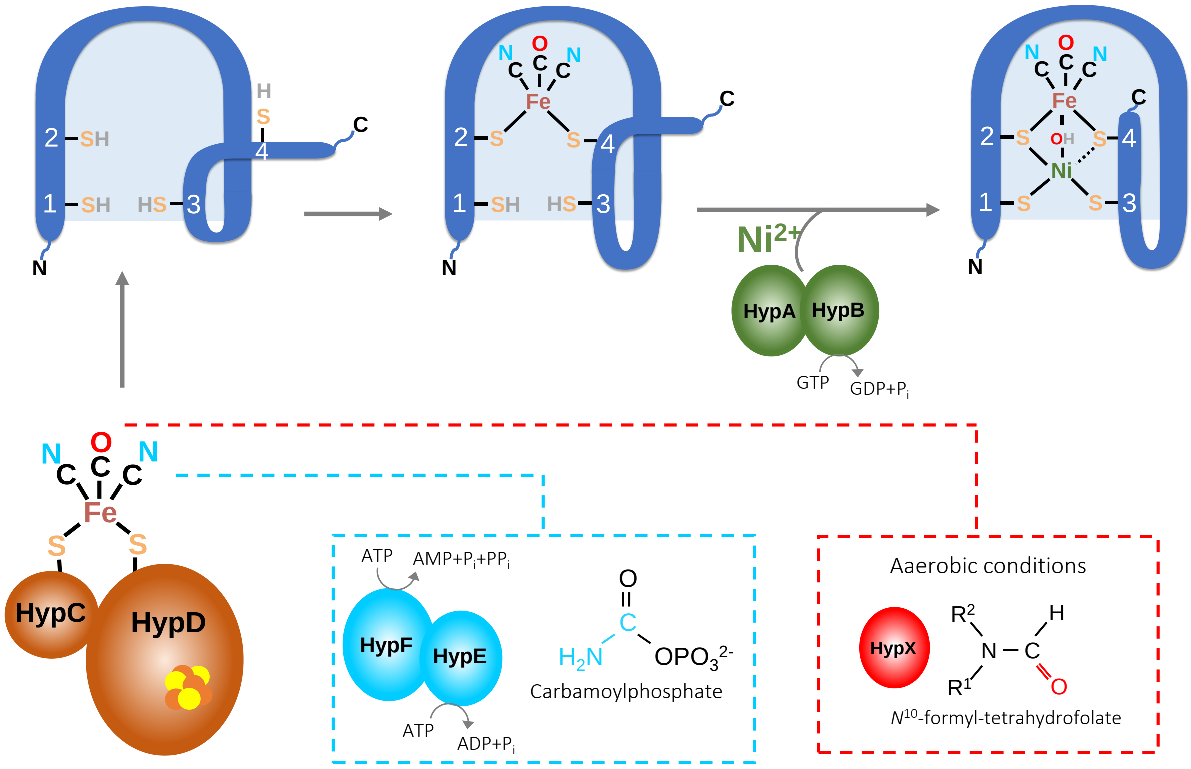

Figure S1. Schematic overview of the sequential steps of [NiFe]-hydrogenase active site metalation. The cyanide ligands of the $Fe(CN)_2CO$ cofactor are synthesized by HypE/HypF (light blue) using carbamoylphosphate as substrate. The CO ligand is produced by HypX (red) from N^{10}-formyl-tetrahydrofolate under aerobic conditions (the anaerobic CO synthesis pathway is unknown). The CO/CN^- ligands are anchored to an iron ion, which is presumably bound to the HypCD complex via Cys2 of HypC and Cys38 of HypD (*T. kodakarensis* numbering). After insertion of the $Fe(CN)_2CO$ synthon into the apo-form of the [NiFe]-hydrogenase large subunit (dark blue), the maturases HypA and HypB (green) mediate the insertion of the Ni ion.

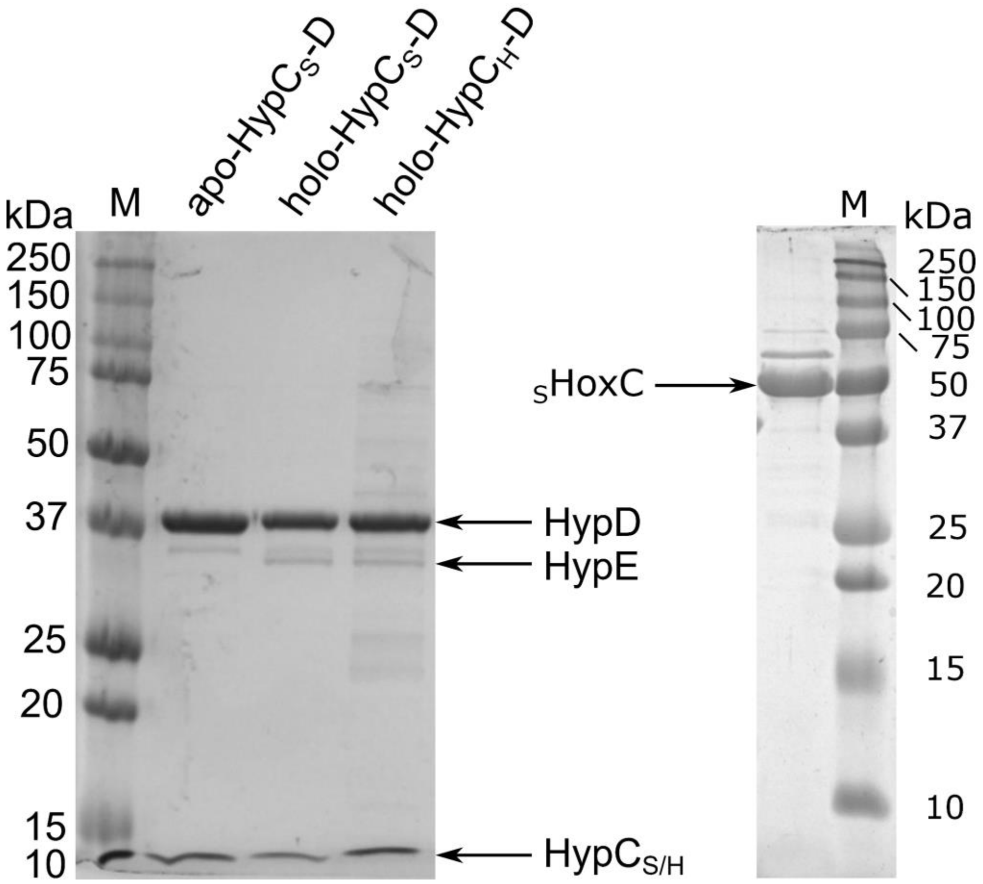

Figure S2. Purified apo-HypC$_S$-D, holo-HypC$_S$-D, holo-HypC$_H$-D and apo-$_S$HoxC separated on an SDS-PAGE gel. The isolated proteins (3.5 µg of protein per lane) were separated on a 15 % SDS-gel. Relevant protein bands are marked with arrows. The molecular masses of $_S$HoxC, HypD and HypC$_{S/H}$ are ~53 kDa, ~41 kDa, and ~10 kDa, respectively. The sizes of the protein marker bands are shown on the left.

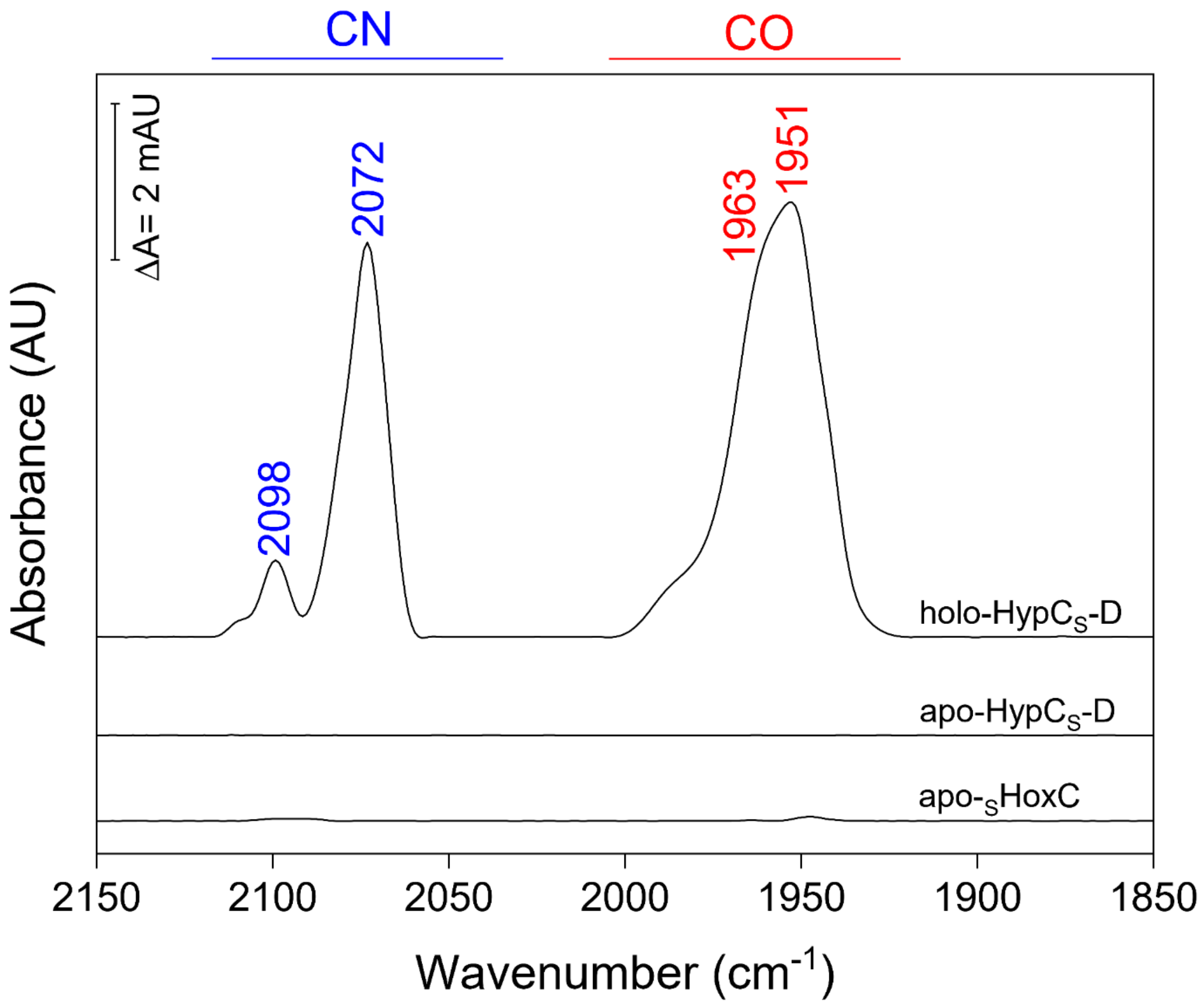

Figure S3. IR-spectroscopic characterization of purified HoxC and HypCD. The spectral region is shown where the stretching vibrations of the CO and CN^{-} ligands occur, which are typically observed in maturation intermediates and fully assembled [NiFe]-hydrogenase. The CN^{-} (blue) and CO (red) IR absorption bands of holo-HypC$_S$-D are labeled with corresponding wavenumbers. Apo-HypC$_S$-D and apo-$_S$HoxC show no peaks in this spectral region. Holo- and apo-HypC$_S$-D spectra are normalized to the intensity of the amide II band. Apo-$_S$HoxC was used in a similar concentration.

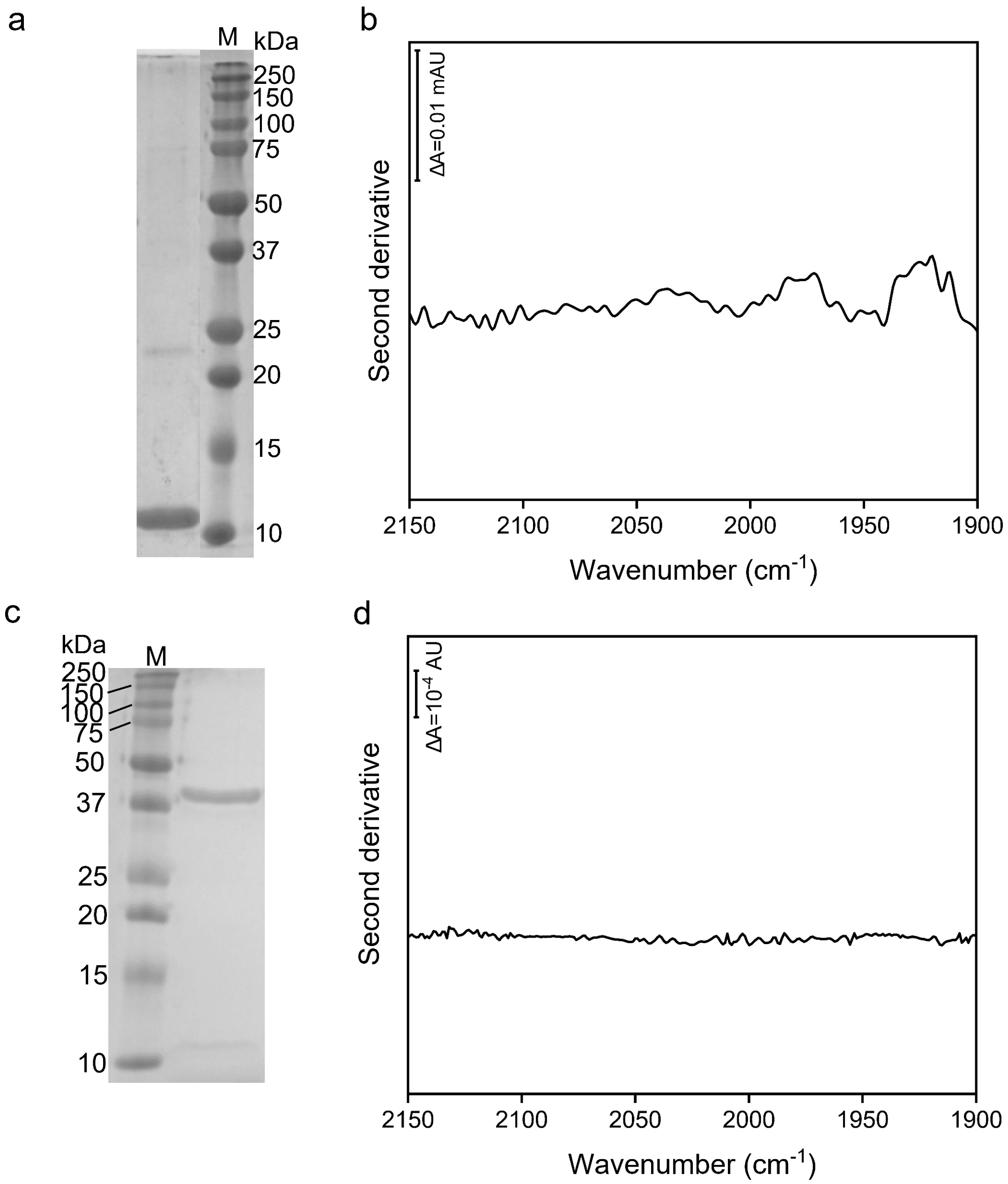

Figure S4. Purification of HypC and HypD and their characterization by IR spectroscopy. (a), (c) SDS-PAGE gels of independently isolated $HypC_S$ and $HypD_H$ proteins (3.5 µg of protein per lane). (b), (d) 2nd derivatives of the IR spectra of the $HypC_S$ and $HypD_H$ proteins. The energy range in which the stretching vibrations of the CO and CN^- ligands occur is shown, indicating the absence of the $Fe(CN)_2CO$ fragment in both proteins. The data in panel (d) contradict earlier observations that suggested the isolated HypD protein has characteristic CO and CN absorptions.

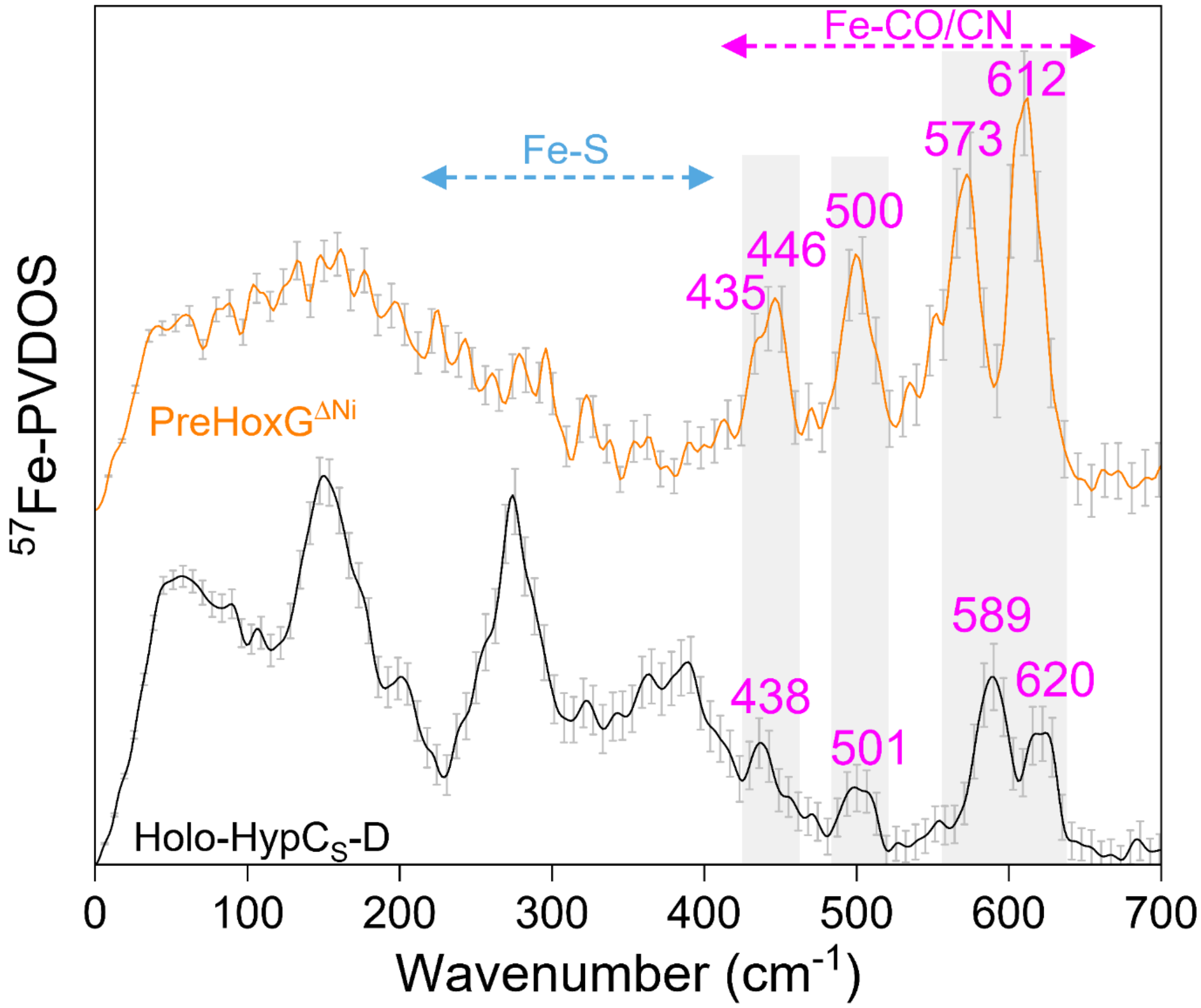

Figure S5. Comparison of the NRVS data of holo-HypC$_S$-D and the maturation intermediate preHoxG$^{\Delta Ni}$. Prominent Fe–CO/CN bands of holo-HypC$_S$-D (black trace) and preHoxG$^{\Delta Ni}$ (orange trace) are labeled with the corresponding wavenumbers. It has previously been shown that the preHoxG$^{\Delta Ni}$ intermediate of the large subunit of the membrane-bound [NiFe]-hydrogenase from *C. necator* carries the Fe(CN)$_2$CO moiety of the active site.[2]

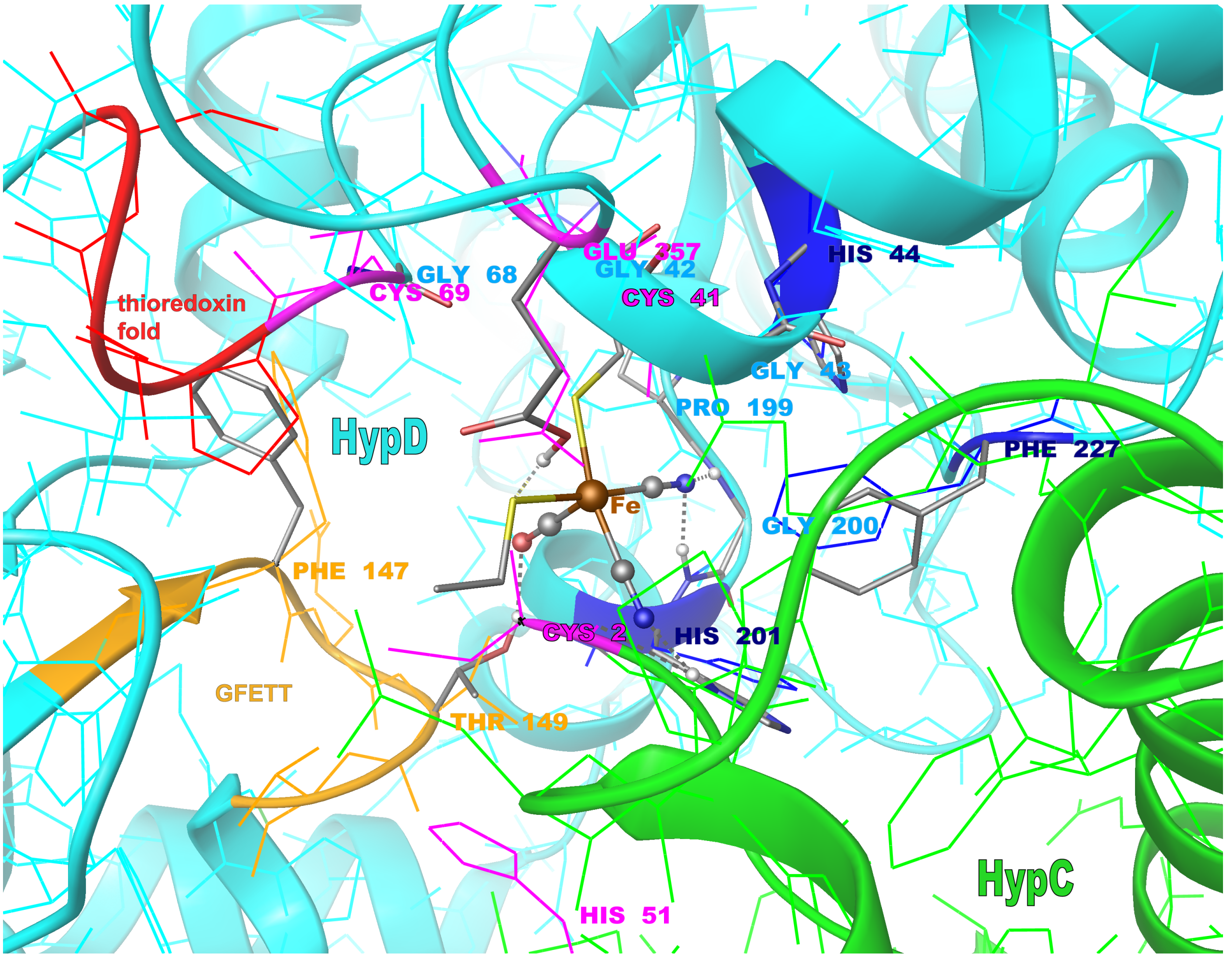

Figure S6. DFT model of the $Fe(CN)_2CO$ cofactor binding region in *Ec*HypCD. The DFT-optimized structure (tube and ball representation, element colors) is overlaid with the cofactor-free AlphaFold 2 model of the protein (ribbon & wire representation). HypC and HypD of the AlphaFold 2 structure are colored light green and light blue. Specific colors were assigned to residues that (*i*) are involved in binding either the $Fe(CN)_2CO$ cofactor or ATP based on this work and previous studies[3,4] (magenta), (*ii*) form the conserved GFETT motif (orange), (*iii*) form the conserved thioredoxin fold (red), (*iv*) are located within 3 Å distance of the cofactor (deep blue), according to the *Tk*HypCD model by Albareda et al.[5] Protons are not displayed, except those forming hydrogen bonds (···) with the $Fe(CN)_2CO$ cofactor.

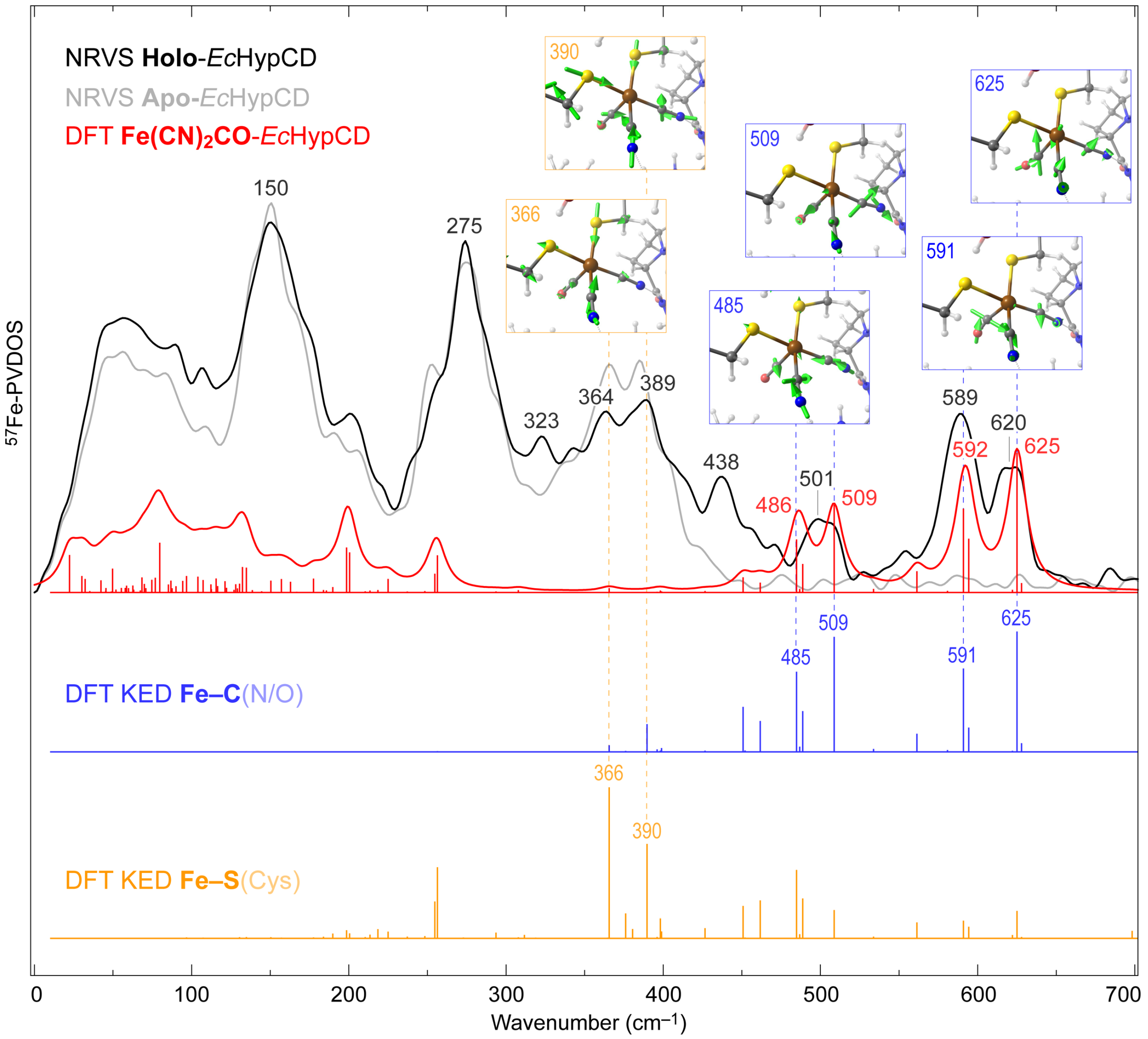

Figure S7. ^{57}Fe-PVDOS of *Ec*HypCD from NRVS experiments and DFT modeling. Top: ^{57}Fe-PVDOS from the NRVS experiments on holo-HypCD (black), apo-HypCD (gray), as well as DFT calculations on the $Fe(CN)_2CO$ model (red) depicted in **Figure 2a**. The experimental intensities were re-normalized to the number of Fe sites, implying a 5/4 ratio for the integral intensities of the holo-/apo-protein spectra. Bottom: DFT-based kinetic energy distributions (KED) revealing Fe–C(N/O) (blue) and Fe–S(Cys) (orange) metal-ligand characters in the calculated vibrational modes. Selected modes of these characters are shown above in arrow-style representation. Animations of these and other normal modes are available as part of the **Supplementary Data III**.

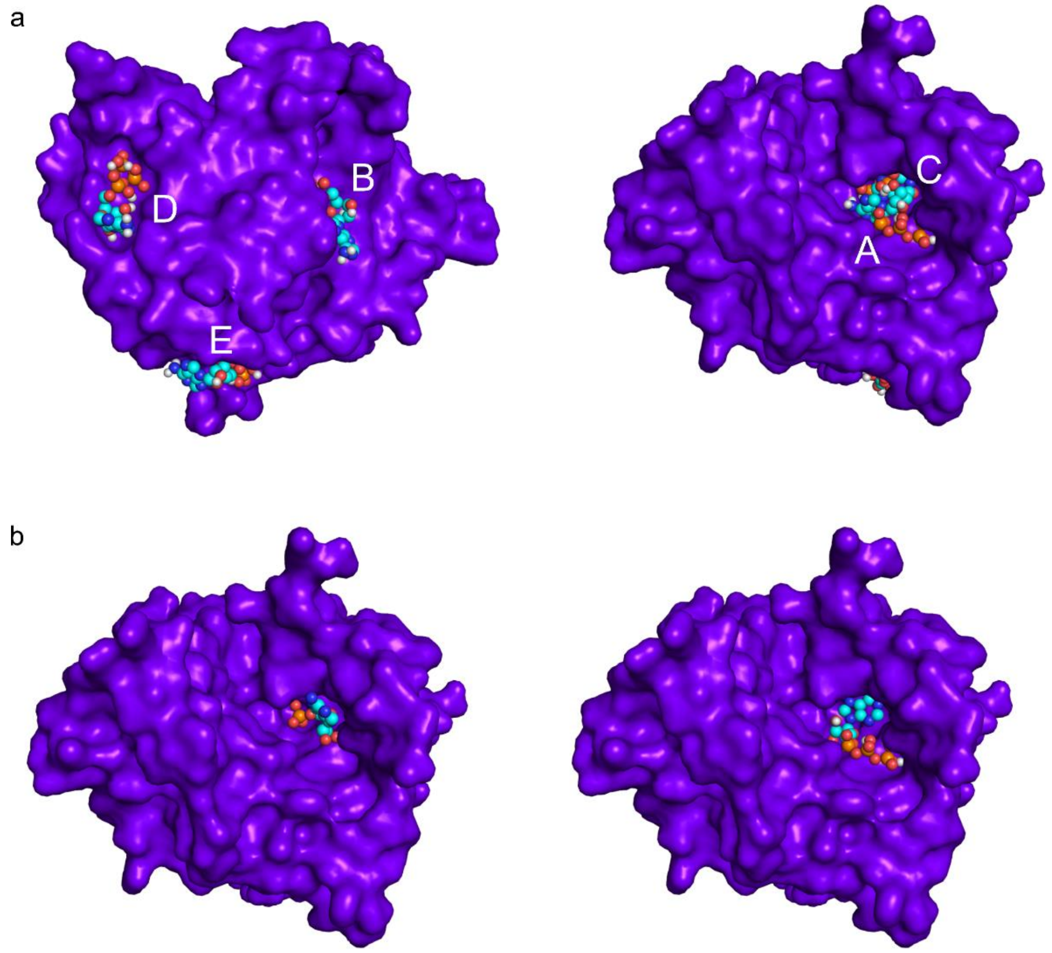

Figure S8. ATP docking to HypE from *T. kodakarensis*. X-ray coordinates of HypE were taken from PDB: 3WJR.[6] Nucleotides are depicted as spheres. (a) Five predicted docking sites (A-E) for ATP were ranked by CB-Dock2 according to their Vina score. Note that the cavities A and C partially overlap. The residues located near the predicted ATP binding sites are shown in **Table S6**. (b) Left: Experimentally determined binding site of the nucleotide (AMP) based crystallographic data of HypE.[6] Right: ATP bound in Cavity C, as predicted by CB-Dock2.

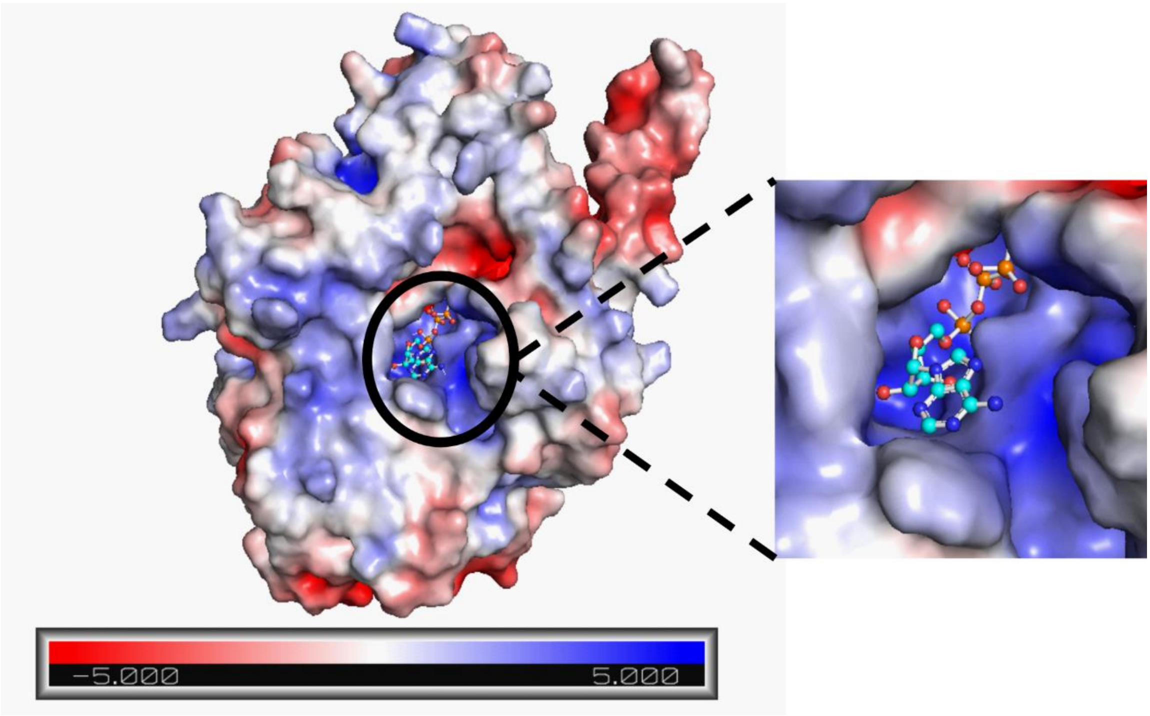

Figure S9. Electrostatic potential surface analysis of HypCD. The electrostatic surface analysis of *Tk*HypCD at ± 5 $k_B T/e$ (pH 7.0, 150 mM ionic strength) was done with the APBS tool of PyMol (The PyMol Molecular Graphics System, Version 2.5.5 Schrödinger, LLC). Blue and red colors indicate positively and negatively charged areas of the protein surface, respectively. ATP is depicted as a ball and stick model, located in cavity A.

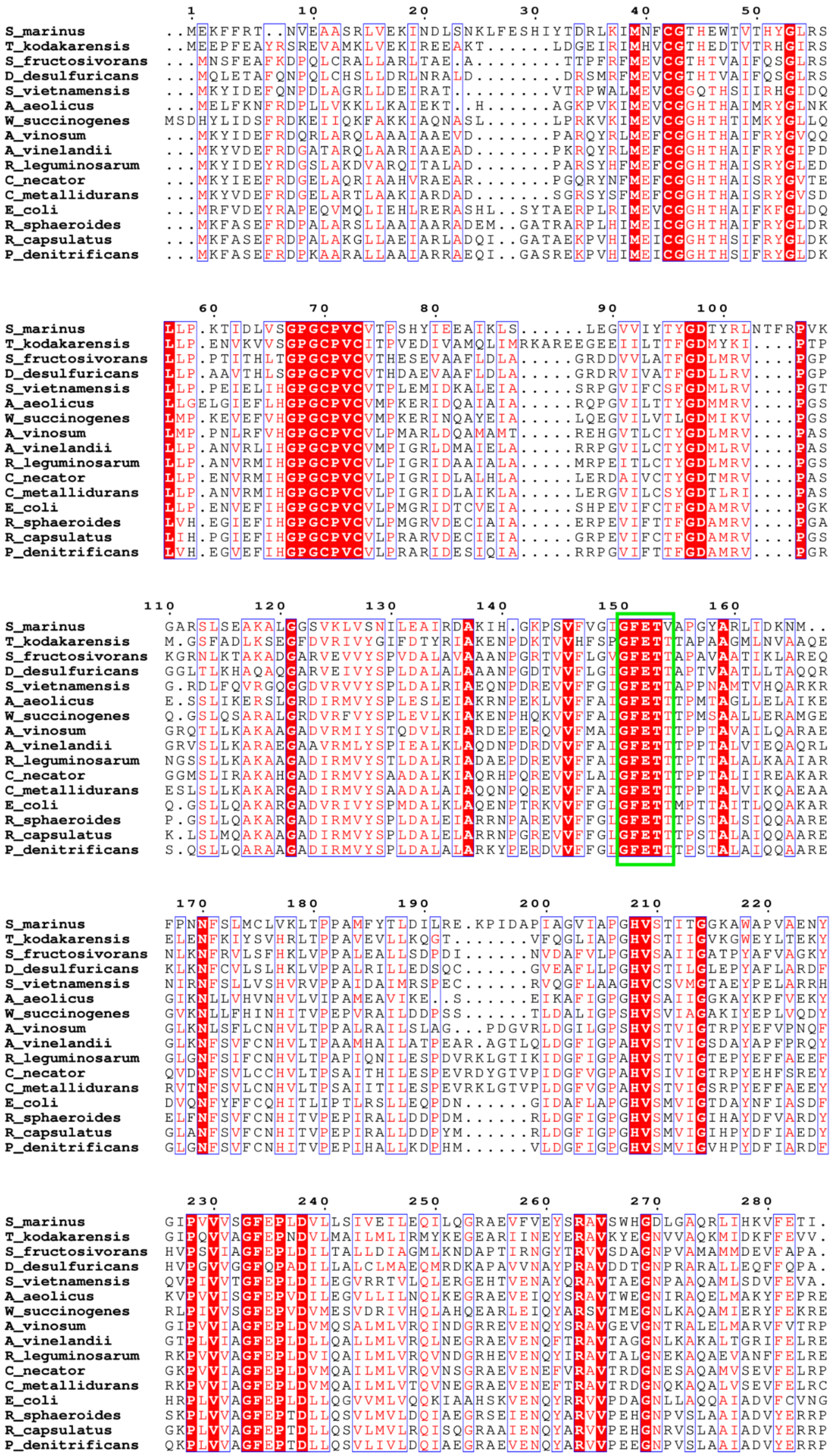

Figure S10. Multiple Sequence Alignment of HypD from various species. Continued on the next page.

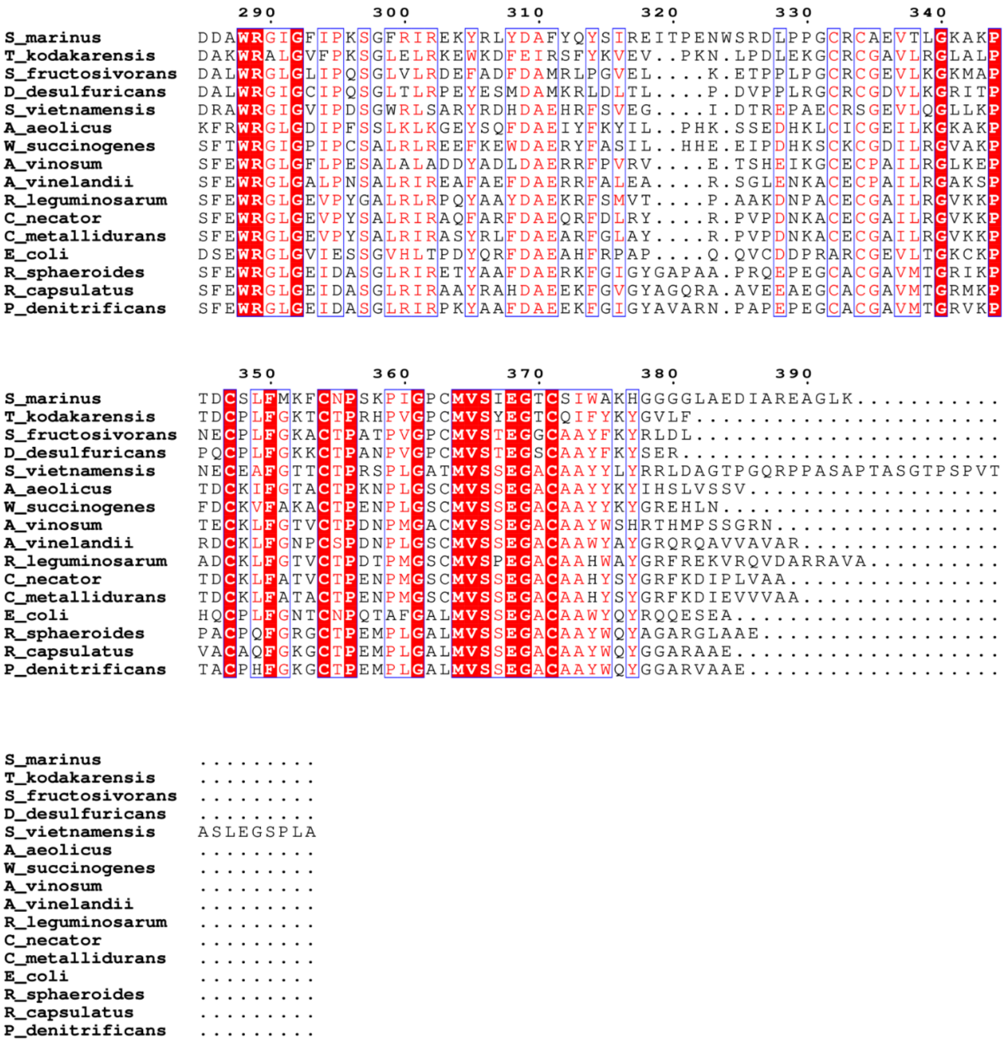

Figure S10. Multiple Sequence Alignment of HypD from various species. Alignment was performed with ClustalOmega. Highly conserved residues, including those of the GPGCPVC[3] and GFETT (green box) motifs are highlighted in red. UniProt accession numbers: *Staphylothermus marinus* (A3DKX4), *Thermococcus kodakarensis* (Q5JII1) *Solidesulfovibrio fructosivorans* (E1K2H9), *Desulfovibrio desulfuricans* (A0A4P7UFU0), *Streptomyces vietnamensis* (A0A0B5I0T4), *Aquifex aeolicus (*A0A9D0YNW7), *Wolinella succinogenes* (Q7M9N5), *Allochromatium vinosum* (D3RQM2), *Azotobacter vinelandii* (P31882), *Rhizobium leguminosarum* (P40598), *Cupriavidus necator* (P31903), *Cupriavidus metallidurans* (Q1LN54), *Escherichia coli* (P24192), *Sporomusa sphaeroides* (A0A1U7MBZ1), *Rhodobacter capsulatus* (P26411), *Paracoccus denitrificans* (A1B6P8).

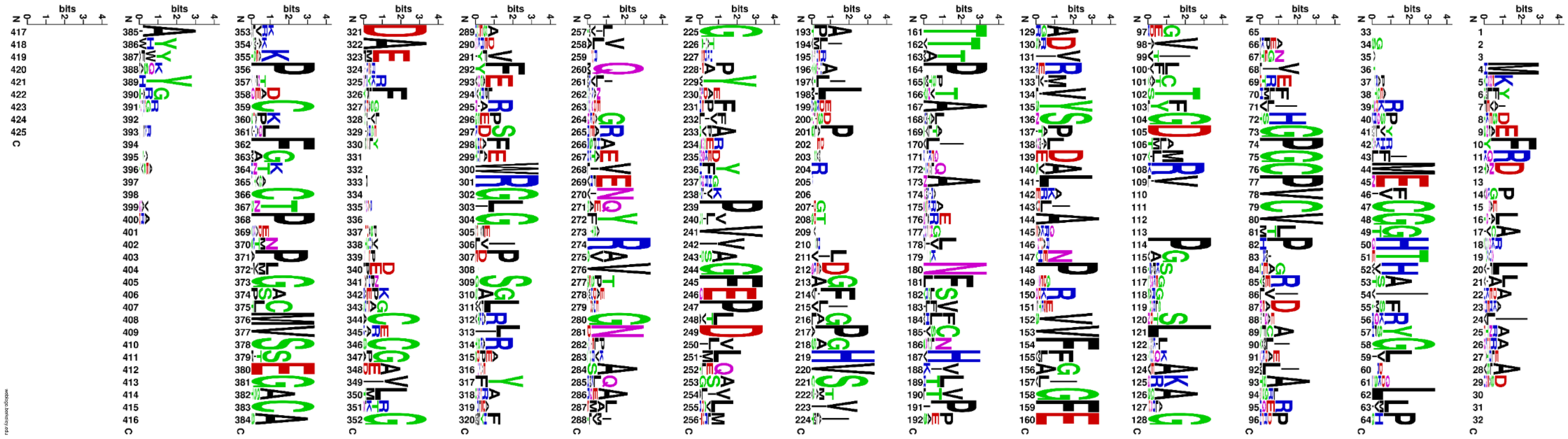

Figure S11. Sequence logo derived from the multiple sequence alignment of HypD. The multiple sequence alignment shown in Error! Reference source not found. served as the basis for this plot, which was created with the WebLogo online tool (Version 2.8.2.).[7] The size of letters is directly related to the abundance of the respective amino acid residue in all HypD sequences. The GFETT motif is one of the most conserved, almost invariant, motifs.

Supplementary Note

ATPase activity measurements were performed with a coupled reaction assay involving pyruvate kinase (PK) and lactate dehydrogenase (LDH) (**Figure S12a**).[8] It appears that HypCD protein purified from cells grown under anaerobic or aerobic conditions exhibits approximately the same ATPase activity when purified under non-reducing conditions (**Figure S12b,** purification: –). This suggests that the growth conditions have no effect on the ATPase activity.[9] HypCD purified from anaerobically grown cells under reducing conditions showed the highest ATPase activity of about 12 mU per mg protein (**Figure S12b,** purification: +DTT), independent of $Fe(CN)_2CO$ loading. To provide additional evidence for the hydrolysis of ATP catalyzed by HypCD, we also recorded IR spectra of ATP in the absence or presence of HypCD to evaluate the absorbances of the reactant and the hydrolysis products (**Figure S13**). ATP and (holo-)HypCD were mixed and transferred to an IR cell under anaerobic conditions. Over time, a gradual increase of the hydrolysis products ADP and phosphate (P_i) was observed (**Figure S13a**), as evidenced by the decrease of the absorbance of PO_2^{-} species and the increase of absorbance related to PO_3^{2-} species.

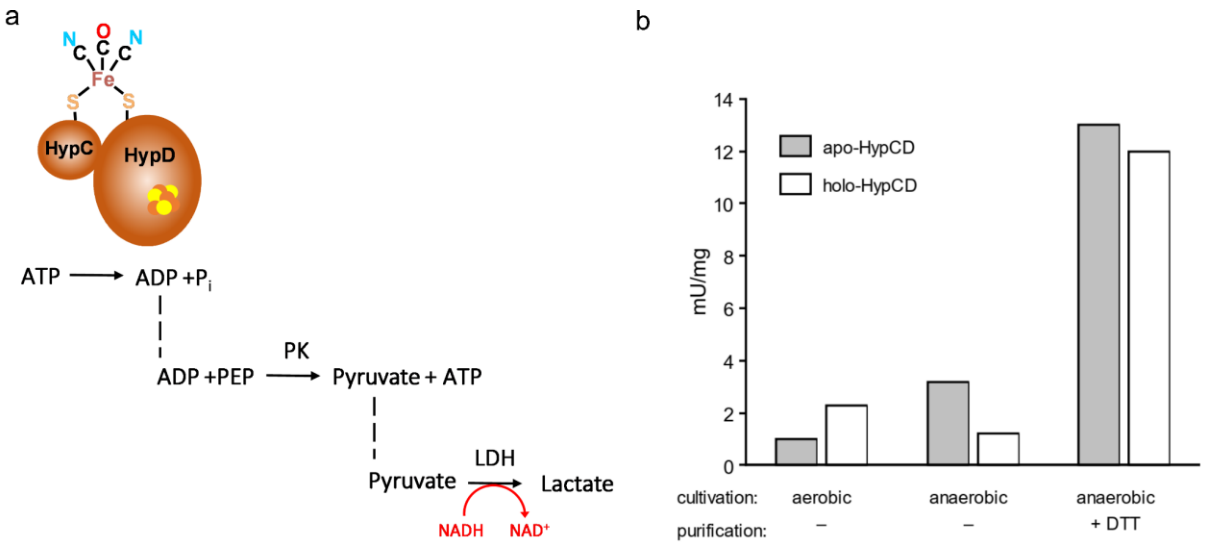

Figure S12. ATPase activity of the HypCD complex determined by a coupled enzyme assay. (a) Overview of the coupled ATPase activity assay, involving pyruvate kinase (PK) and lactate dehydrogenase (LDH). The ATP-mediated decrease of absorption of NADH was measured spectrophotometrically.[8] (b) ATPase activity of apo- and holo-HypCD purified from aerobically and anaerobically grown cells, either in the absence or presence of DTT.

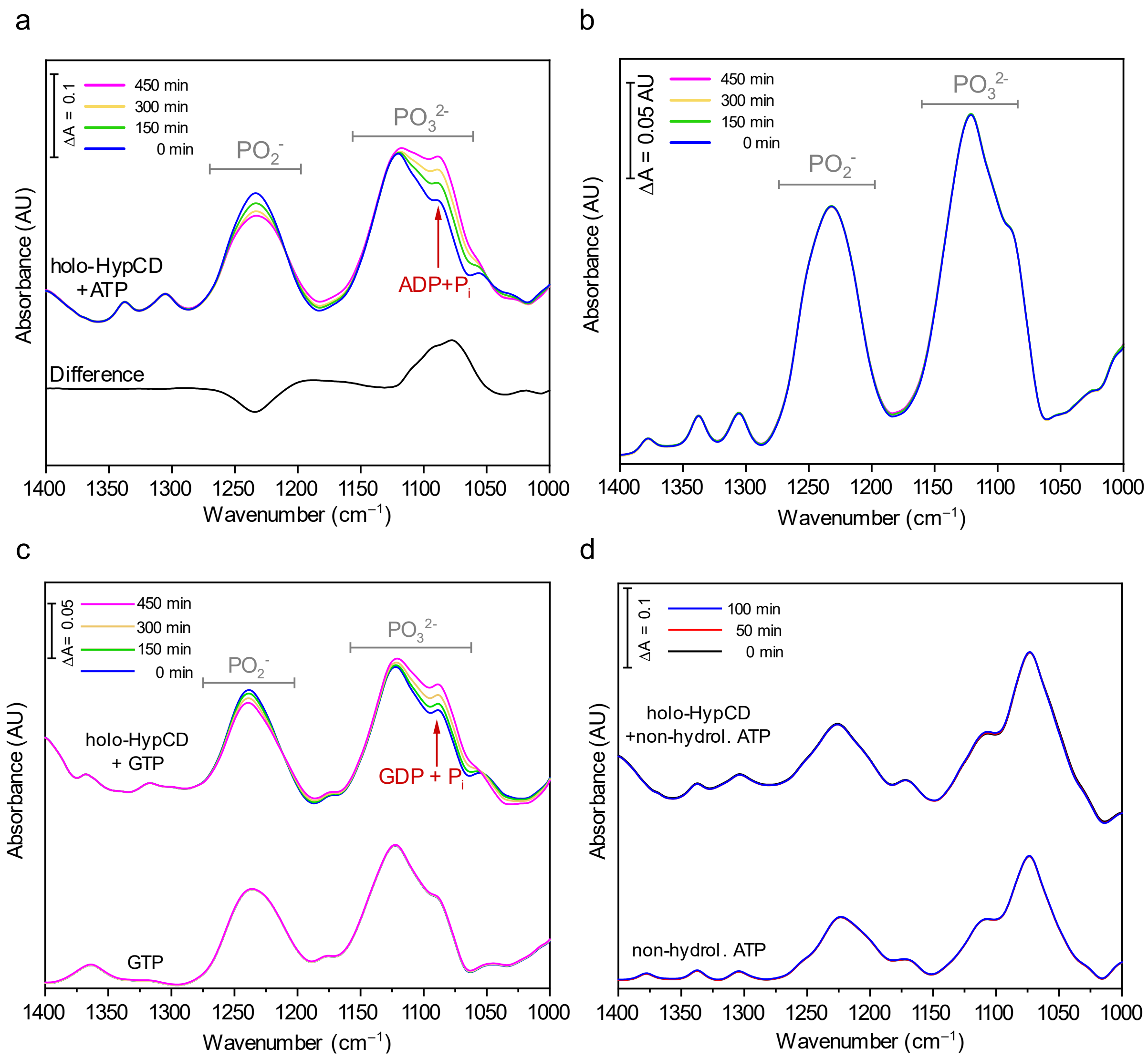

Figure S13. Nucleotide hydrolysis catalyzed by HypCD monitored by IR spectroscopy. (a) ATP (20 mM final concentration), holo-HypC$_H$-D (0.5 mM), NaDT (15 mM) and $MgCl_2$ (10 mM) were mixed anaerobically with a 100 mM Tris/HCl (pH 8 at 4°C), 100 mM NaCl buffer in a total volume of 30 µl, which was immediately transferred to a gas-tight IR transmission cell. Single absorbance spectra obtained 0 min (blue), 150 min (green), 300 min (yellow) and 450 min (magenta) after mixing are displayed. The increase of the hydrolysis products adenosine diphosphate (ADP) and phosphate (P_i) is indicated by a red arrow. The difference spectrum between "time 450 min" and "0 min" (black trace) makes this clearer. (b) IR spectra of ATP without HypCD. ATP (20 mM) was prepared in 100 mM Tris (pH 8 at 4°C) and 100 mM NaCl in a final volume of 30 µl, which was immediately transferred to IR cell. The spectra collected at various time points showed no change over time. This demonstrates stability of ATP under the experimental conditions. (c) IR spectra of 0.5 mM holo-HypC$_H$-D incubated with 20 mM GTP for 0 (blue), 150 (green), 300 min (yellow) and 450 min (magenta) are shown in the upper part, and 20 mM GTP alone is shown in the lower part. The increase of the hydrolysis products guanosine diphosphate (GDP) and phosphate (P_i) is indicated by a red arrow. HypCD hydrolyzes GTP, but to a lesser extent than ATP (d) IR spectra of 0.5 mM holo-HypC$_H$-D incubated with 20 mM β,γ-methyleneadenosine 5′-triphosphate for 0 (black), 50 (red) and 100 min (blue) are shown in the upper part, and 20 mM β,γ-methyleneadenosine 5′-triphosphate alone is shown in the lower part. The spectra collected at different time points showed no changes over time and overlapped completely, indicating that the ATP analogue is not hydrolyzed by Holo-HypCD.

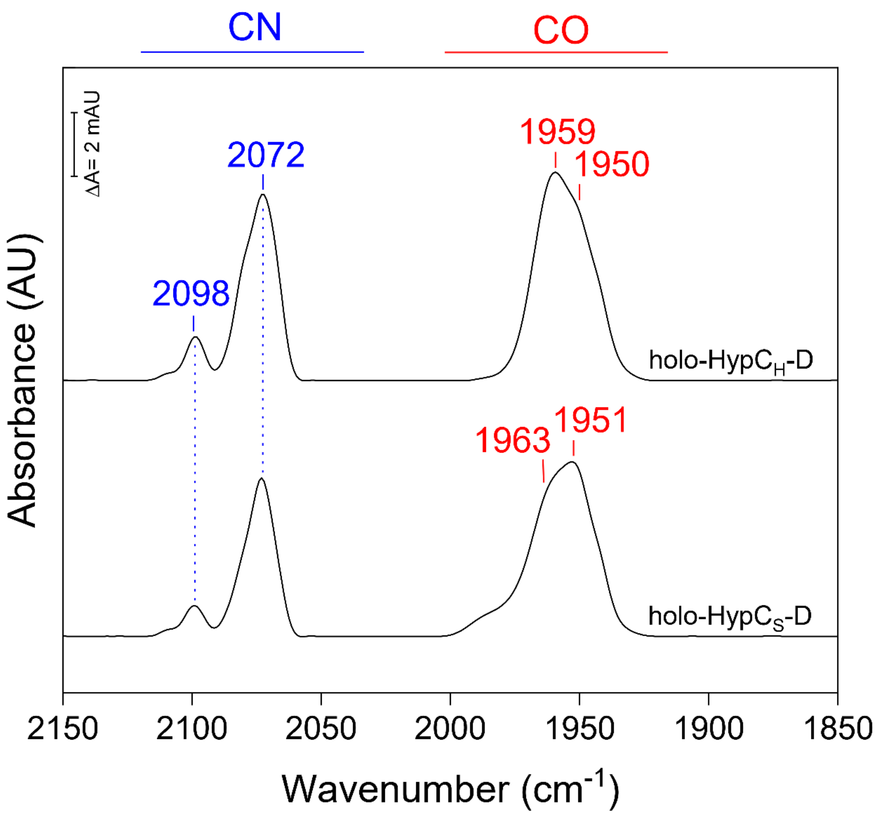

Figure S14. IR spectra of Strep- and His-tagged holo-HypCD. CO and CN^--related bands are labeled with the corresponding wavenumbers. IR spectra are normalized relative to the amide II band intensity.

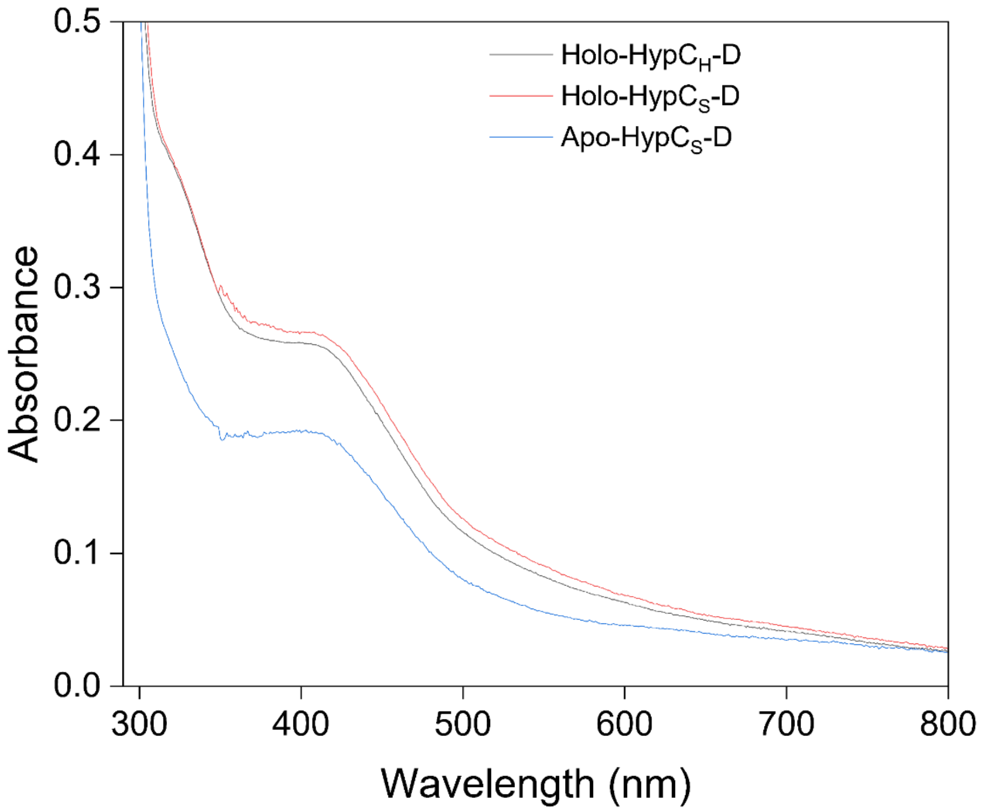

Figure S15. UV-vis spectra of holo-HypC$_H$D, holo-HypC$_S$D and apo-HypC$_S$D. All HypCD versions were used at a final concentration of approximately 40 µM and exhibited a strong absorbance around 410-430 nm, which can be assigned to the [4Fe–4S] cluster of HypD.

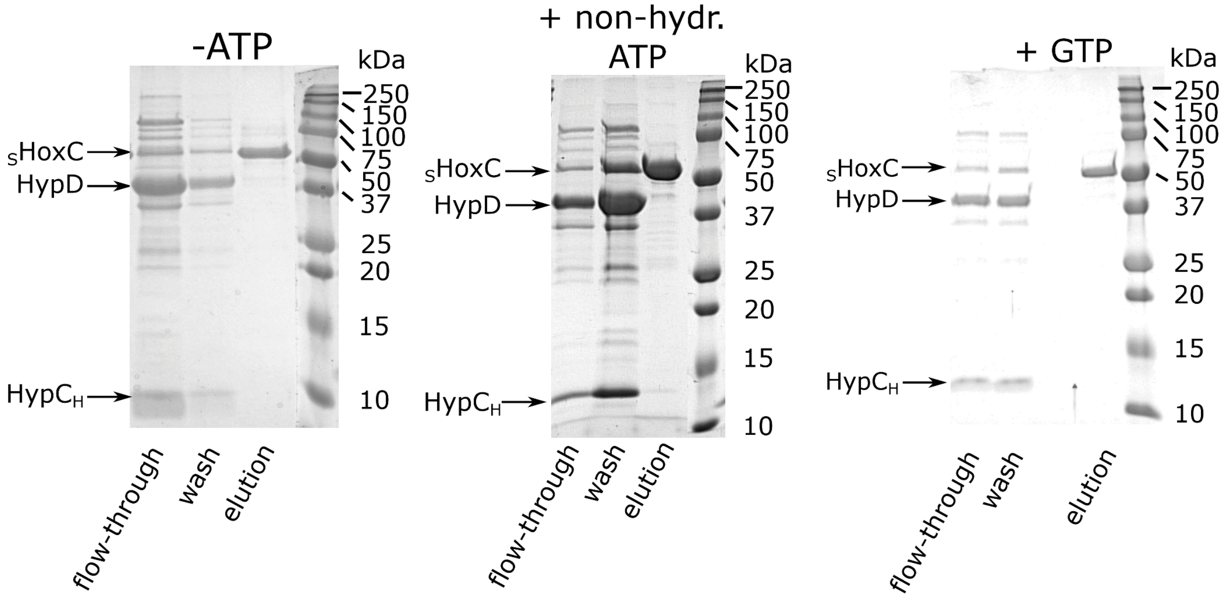

Figure S16. sHoxC protein samples purified after incubation with HypC$_H$D in the presence or absence of different nucleotides. Headlines indicate the nature of the added nucleotide. 3.5 µg (–ATP and + GTP) or 5 µg (+ non-hydr. ATP) of protein were separated on a 12.5 % SDS-gel. The different purification fractions are labeled with flow-through, wash and elution. The latter was concentrated and subjected to IR spectroscopy. Since each transfer reaction was reproduced in triplicate, only one representative SDS-PAGE gel is shown.

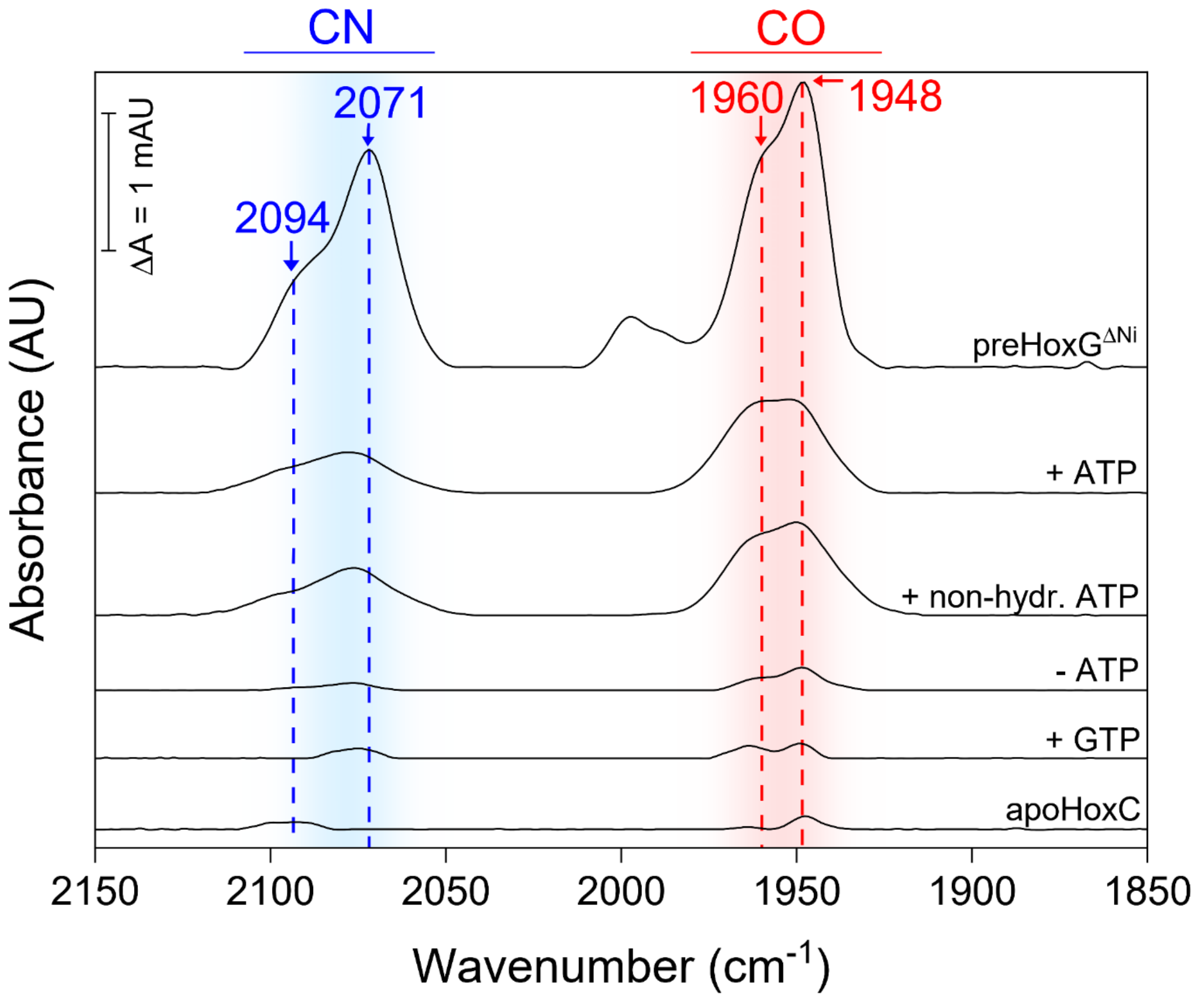

Figure S17. IR spectra of $_{S}$HoxC preparations isolated after the *in vitro* transfer experiments compared to that of preHoxG$^{\Delta Ni}$. IR spectra of preHoxG$^{\Delta Ni}$, a large subunit maturation intermediate of the membrane-bound hydrogenase (MBH) from *C. necator* purified from living cells,[2] and those of $_{S}$HoxC from different *in vitro* transfer assays are shown as black traces. CO and CN^{-}-related band positions (dashed lines) of preHoxG$^{\Delta Ni}$ are labeled with the corresponding wavenumbers.

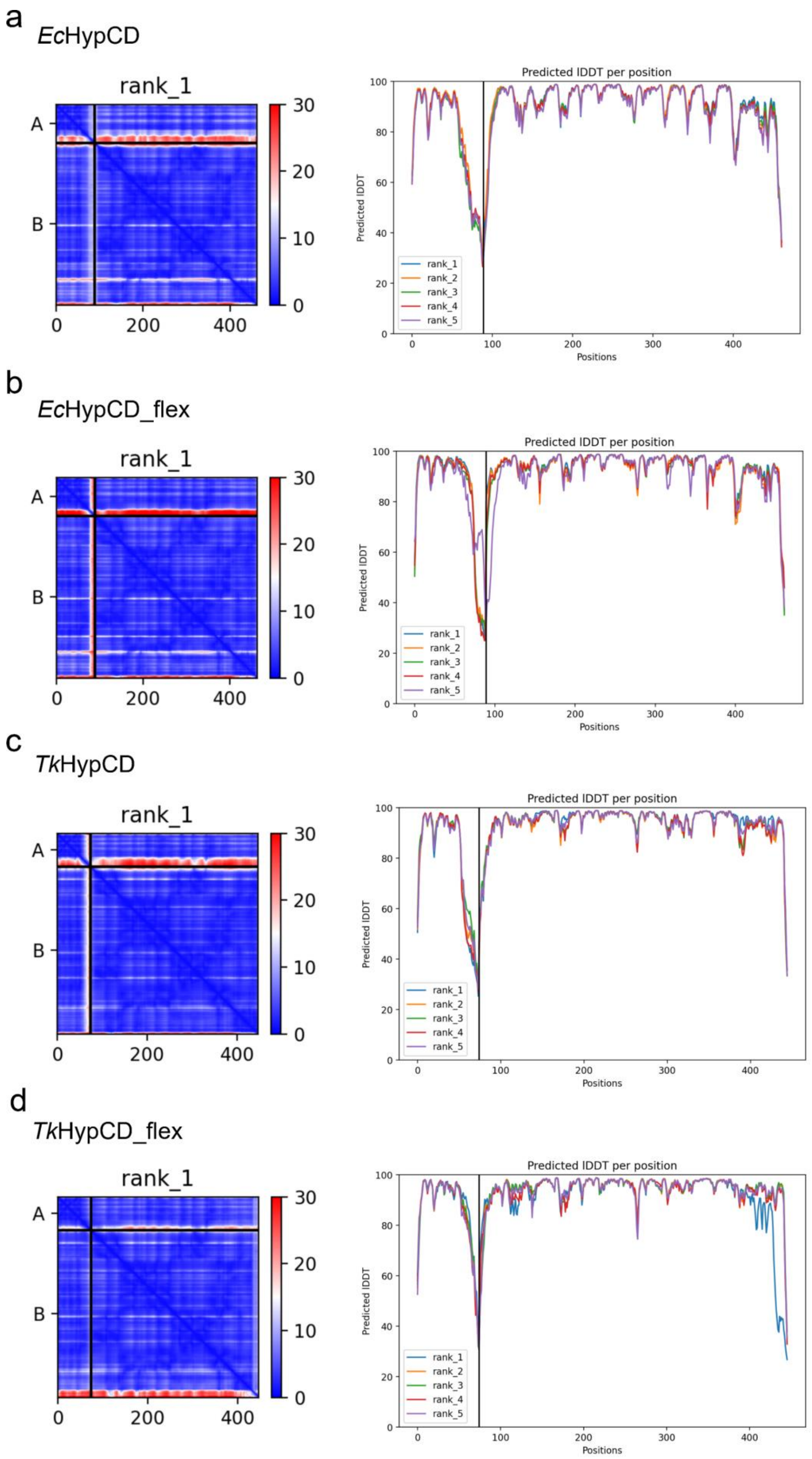

Figure S18. Predicted Aligned Error (PAE) analysis and predicted local distance difference test (pLDDT) from the AlphaFold structural predictions. Continued on the next page.

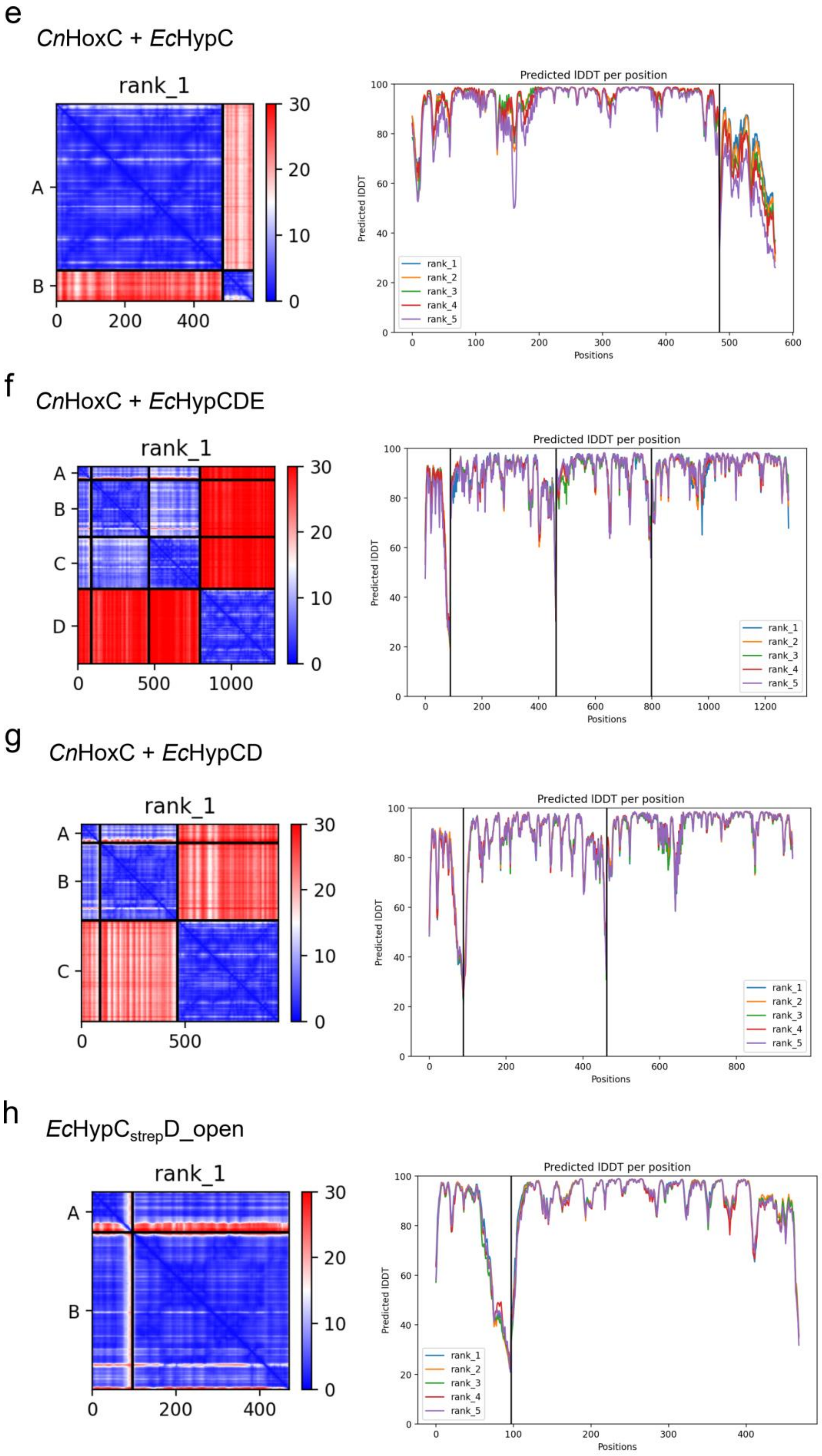

Figure S18. Predicted Aligned Error (PAE) analysis and predicted local distance difference test (pLDDT) from the AlphaFold structural predictions. Continued on the next page.

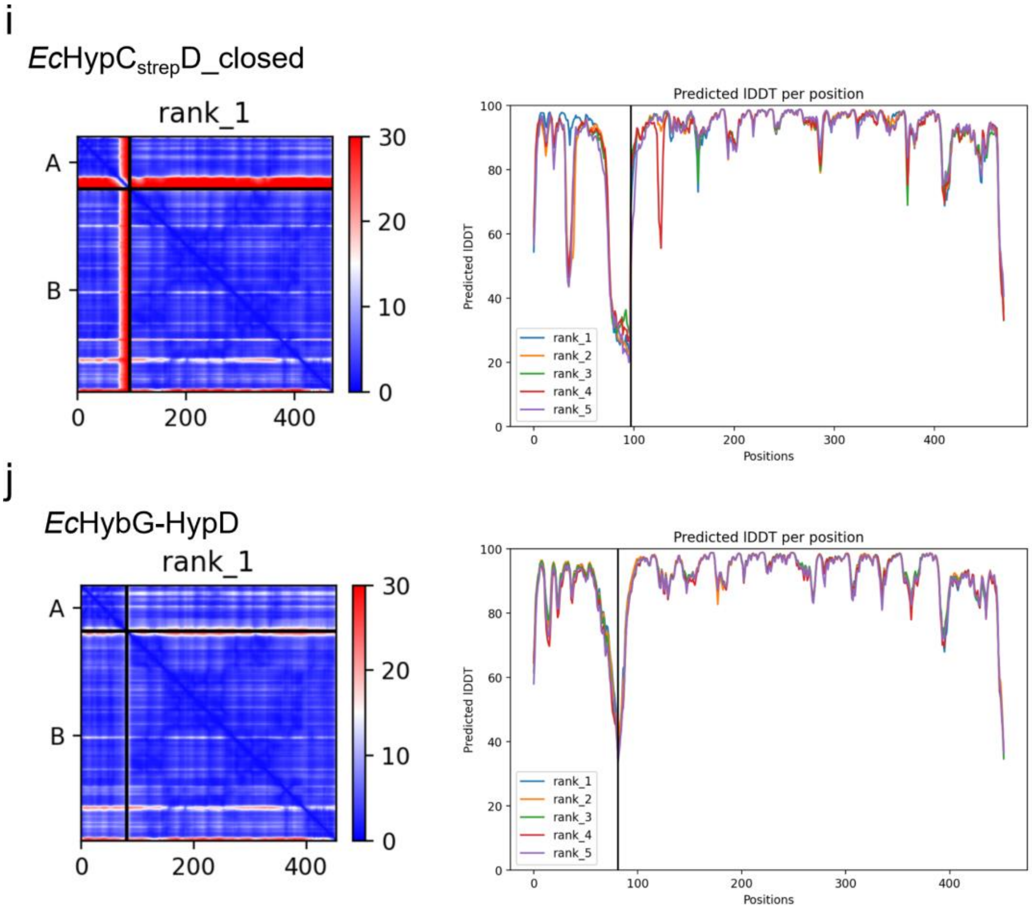

Figure S18. Predicted Aligned Error (PAE) analysis and predicted local distance difference test (pLDDT) from the AlphaFold 2 structural predictions. The PAE is a measure of how reliably AlphaFold can estimate the position and thus the distance of two residues of a protein or protein complexes, respectively. It is given in Ångstrom between 0 (blue, high confidence) and 30 (red, low confidence) and displayed as a heat-map. The pLDDT can be considered as a confidence score to estimate the correct placement of a residue in its local environment. The scores rank between 0-100 (y-axis), with values below 50 indicating low confidence (https://alphafold.ebi.ac.uk/faq). Only structure predictions with the highest pLDDT score were used for all downstream *in silico* analyses (e.g., CB-Dock2). (a) *E. coli* HypCD. (b) *E. coli* HypCD with alternative flexibility parameters as described in the Methods section of the manuscript (c) *T. kodakarensis* HypCD. (d) *T. kodakarensis* HypCD with alternative flexibility parameters as described in the Methods section of the manuscript. (a-d) Sequence A corresponds to HypC, sequence B to HypD (e) *C. necator* HoxC and *E. coli* HypC. Sequence A corresponds to HoxC, sequence B to HypC. (f) *C. necator* HoxC and *E. coli* HypCDE. Sequence A corresponds to HypC, sequence B to HypD, sequence C to HypE and sequence D to HoxC. (g) *C. necator* HoxC and *E. coli* HypCD. Sequence A corresponds to HypC, sequence B to HypD, and sequence C to HoxC. (h) Strep-tagged *E. coli* HypCD in its closed conformation. Sequence A corresponds to HypC, sequence B to HypD. (i) Strep-tagged *E. coli* HypCD in its open conformation. Sequence A corresponds to HypC, sequence B to HypD. (j) *E. coli* HybG-HypD. Sequence A corresponds to HybG and sequence B corresponds to HypD.

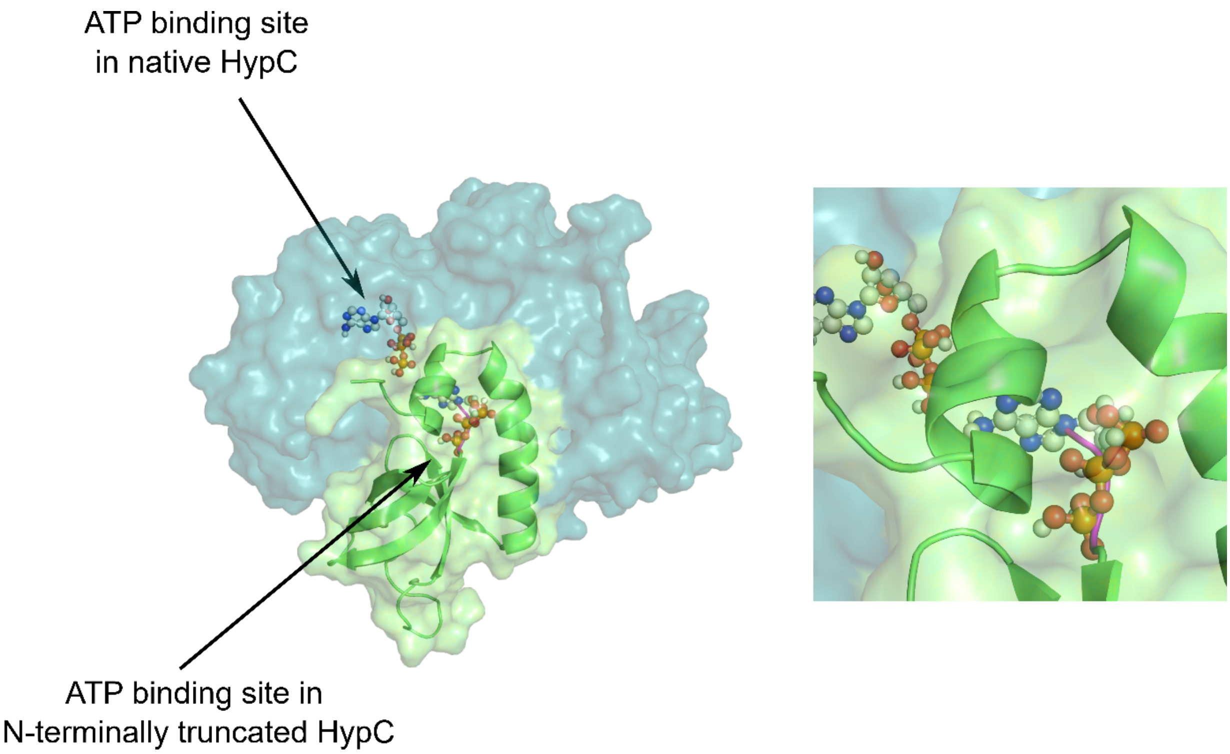

Figure S19. Superimposed AlphaFold 2 structures of *Ec*HypCD with and without N-terminally truncated HypC. Truncated HypC lacks Cys2 and Ile3. HypC is depicted in cartoon representation (green). HypD is depicted in petrol and ATP is depicted in sphere representation. Note the different predicted binding sites (each assigned with lowest Vina-Score in **Tables S7** and **S8**) depending on the presence/absence of Cys2. The enlargement on the right shows the N-terminal residues Cys2 and Ile3 (magenta), which sterically hinders ATP binding in the presence of the native version of HypC.

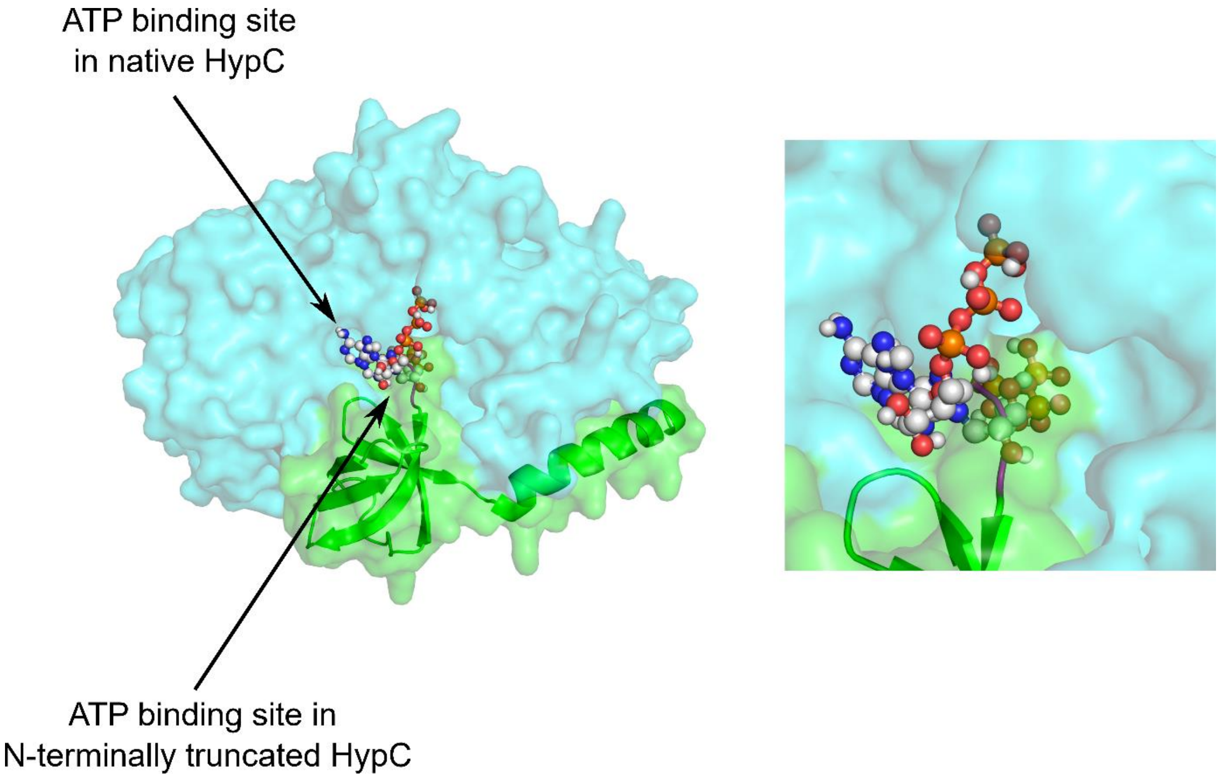

Figure S20. Superimposed AlphaFold 2 structures of *Tk*HypCD with and without N-terminally truncated HypC. Truncated HypC lacks Cys2 and Ile3. HypC is depicted in cartoon representation (green). HypD is depicted in cyan and ATP is depicted in sphere representation. Note the different predicted ATP binding sites (each assigned with the lowest Vina-Score in **Tables S9 and S10**) depending on the presence/absence of Cys2. The enlargement on the right shows the N-terminal residues Cys2 and Ile3 (magenta), which sterically hinder ATP binding in the presence of the native version of HypC.

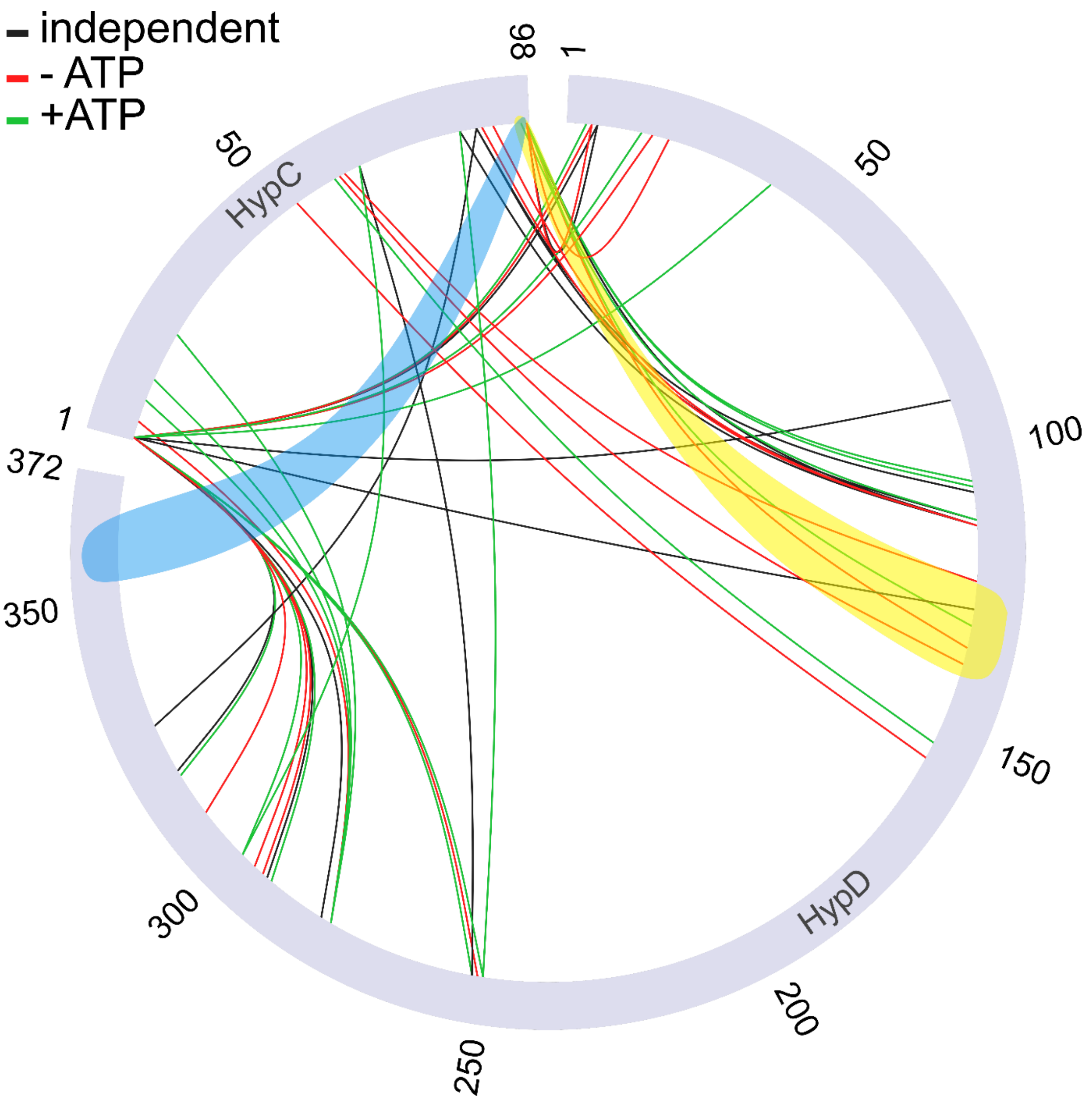

Figure S21. Crosslinking MS data on apo-HypC_S-D incubated with and without ATP. Cross-linking mass-spectrometry analysis of apo-HypCD incubated with and without ATP. HypC and HypD sequences are depicted in gray true-to-scale bars and are labeled accordingly. Amino acid numbering is given on the outer side of the circle. Cross-links between the two proteins are depicted as red (–ATP) and green (+ ATP) lines. If crosslinks are observed in both samples, they are depicted as black lines (independent). Blue and yellow shaded areas mark the regions where crosslinks supporting the closed conformation were observed for holo-HypCD (**Figure 6a**).

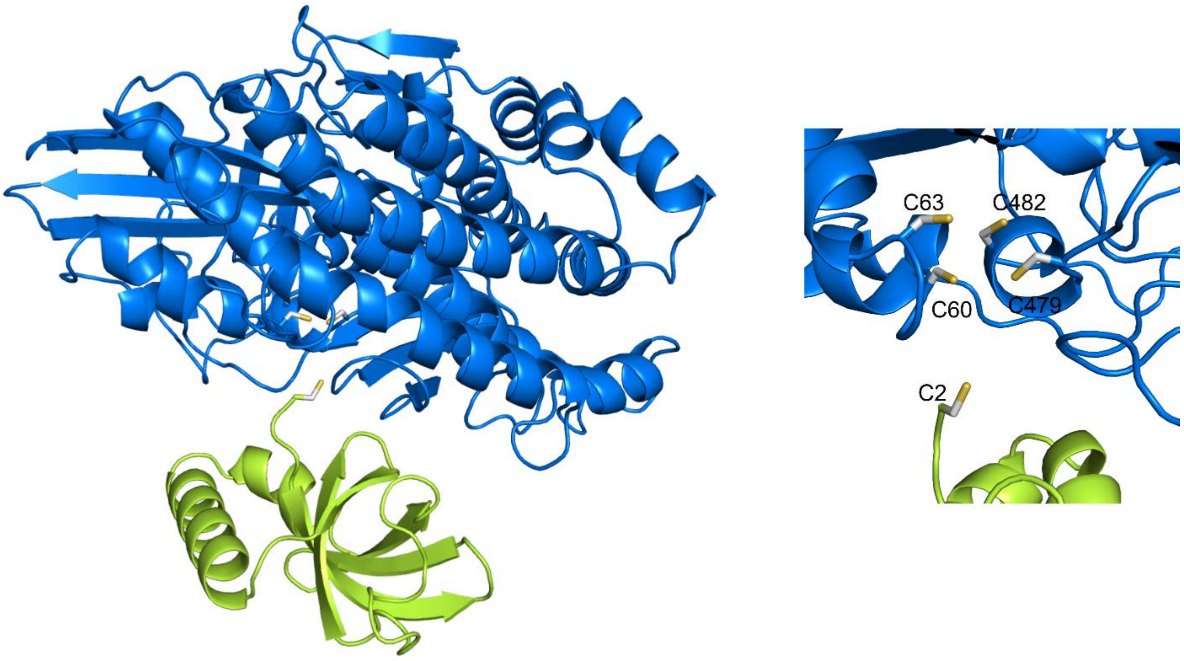

Figure S22. AlphaFold 2-predicted complexes between *Ec*HypC and *Cn*HoxC. Cys2 of HypC protrudes into the [NiFe]-binding motif of HoxC, comprising the residues Cys60, 63, 479 and 482. HoxC (blue) and HypC (green) are shown in cartoon representations, and the [NiFe]-binding site of HoxC is shown in ball and stick representation with S in yellow; C in gray.

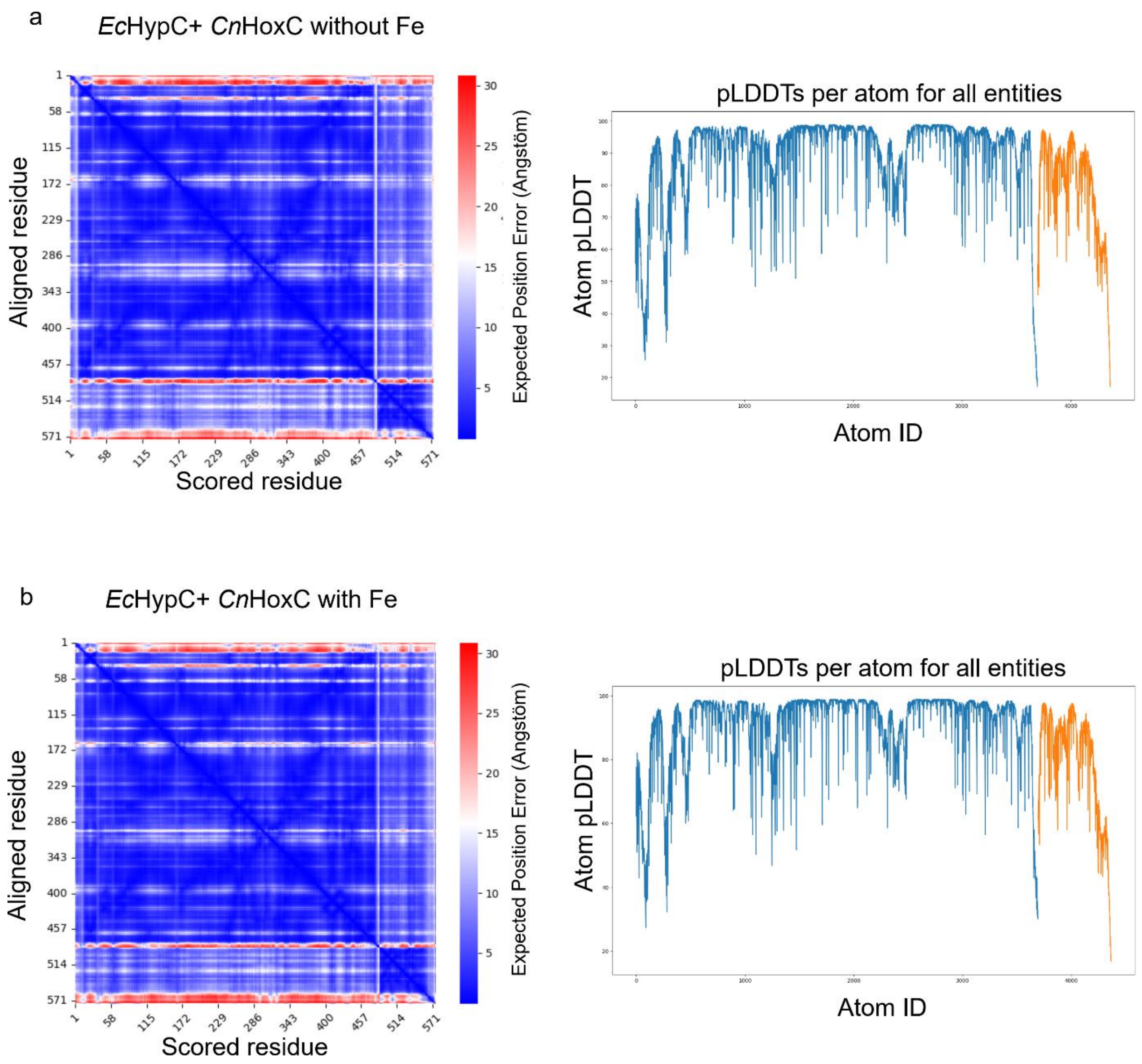

Figure S23. Predicted Aligned Error (PAE) analysis and predicted local distance difference test (pLDDT) for the AlphaFold 3-predicted complexes of *Ec*HypC and *Cn*HoxC with and without iron. (a) *Ec*HypC + *Cn*HoxC without Fe, (b) *Ec*HypC + *Cn*HoxC with Fe. The PAE (left panels) is a measure of how reliably AlphaFold can estimate the position and thus the distance of two residues in a protein or protein complexes, respectively. It is given in Ångstrom, it ranges between 0 (blue, high confidence) and 30 (red, low confidence) and displayed as a heat-map. Both plots in (a) and (b) indicate that the models are more reliable than that computed by AlphaFold 2 (see **Figure S18e**). The pLDDT (right panels) can be considered as a confidence score to estimate the correct placement of a residue (or its atoms) in its local environment. The scores rank between 0-100 (y-axis), with values below 50 indicating low confidence (https://alphafold.ebi.ac.uk/faq). The blue color in the pLDDT represents the HoxC sequence and orange represents the HypC sequence. Note that PAE panels are plotted visualizing the "score per residue" while the pLDDT is plotted per atom for all entities. AlphaFold 3 ranks the predicted structures according to the "predicted template modeling, **pTM**" ranking score, which measures the accuracy of the entire structure. Values higher than 0.8 represent confident high-quality predictions. **pTM** for HypC_HoxC = 0.84.

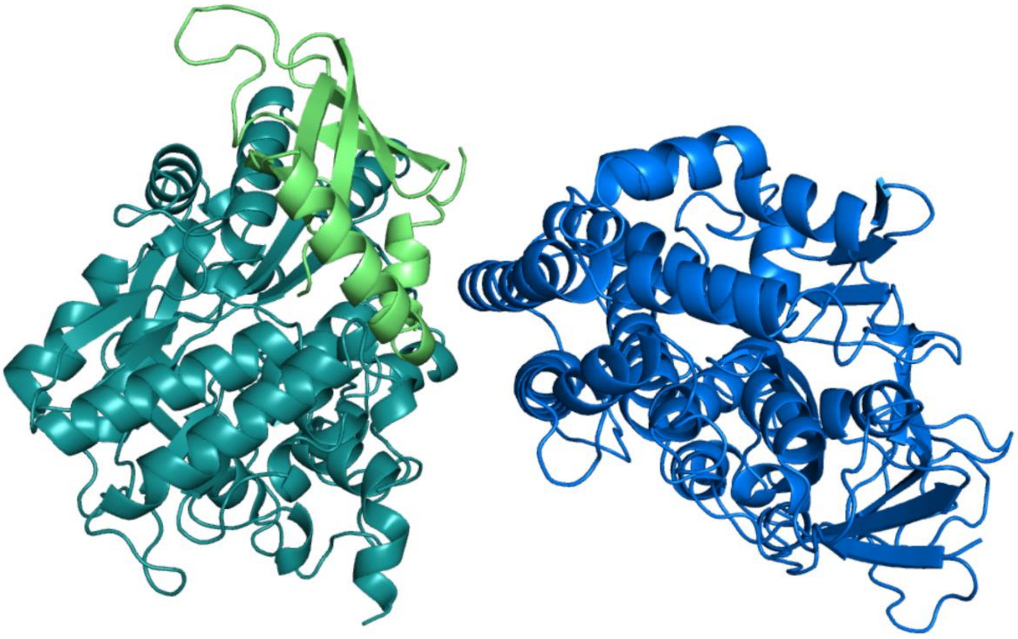

Figure S24. AlphaFold 2 structure prediction of *Cn*HoxC and *Ec*HypCD. HoxC, HypC and HypD are depicted blue, green and petrol, respectively. According to the predicted aligned error (**Figure S18**), no interaction between HoxC and the HypCD complex is predicted.

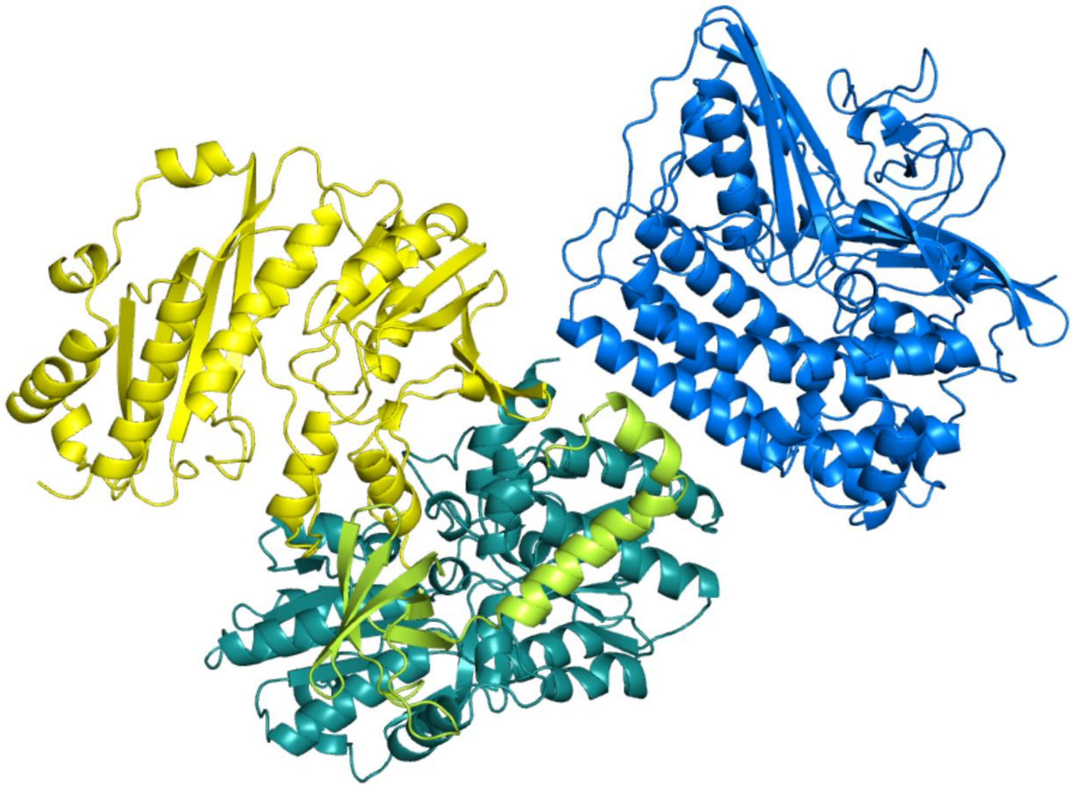

Figure S25. AlphaFold 2 structure prediction of *Cn*HoxC and *Ec*HypCDE. HoxC, HypC, HypD and HypE are depicted blue, green, petrol and yellow, respectively. According to the predicted aligned error (**Figure S18**), no interaction between HoxC and the ternary HypCDE complex is predicted.

Supplementary Methods

For construction of pTS15 (holo-HypC$_H$-D), three DNA fragments were amplified by PCR using the primer pairs 2_Amp_rev/HypDrev, seqHypD/HisHypCDrev_2 and 3_Amp_for/HisHypCDfor_2 (**Table S11**), respectively, and pTHypDEFC$_{strep}$ (**Table S2**) as the template. The three amplicons were subsequently assembled via Gibson assembly (Gibson Assembly Master Mix, New England Biolabs).

For construction of pTS17 (apo-$_S$HoxC), two DNA fragments were amplified by PCR using the primer pairs Strep-HoxC_for/Strep-HoxC_rev and pT7_for/pT7_rev (**Table S11**) together with the templates pJH3647 and pHart13 (**Table S2**), respectively. The two amplicons were subsequently assembled via Gibson assembly.

For construction of pHart13 ($_H$HypD), the primers NdeI_his6_Ec_HypD_fwd_57 and Ec_HypD_HinIII_rev_58 (**Table S11**) were used to amplify a *hypD*-containing fragment from pTHypDC$_{strep}$ (**Table S2**). The PCR product as well as plasmid pT7-7 were digested with NdeI and HindIII, and both fragments were subsequently ligated.

For construction of pAKW19 (HypC$_S$), two DNA fragments were amplified by PCR using the primer pairs AKWp38, AKWp39 and AKWp40, AKWp41, respectively, and pTHypDEFC$_{strep}$ (**Table S2**) as template. The two fragments were subsequently assembled via Gibson assembly (Gibson Assembly Master Mix, New England Biolabs).

The resulting plasmids were transformed into *E. coli* Rosetta (DE3) strains, yielding the strains AK2507 (holo-HypC$_H$-D), AK2628 (apo-$_S$HoxC), AK2608 (HypC$_S$) and AK2627 (Hyp$_H$D), respectively.

Table S1. Protein purification buffers[a]

Protein	Location of affinity tag	Resuspension buffer[b,c]	Wash Buffer (volume given in column volumes (CV))	Elution buffer (volume given in column volumes (CV))	Filter cut-off (kDa)[d]
apo-HypC$_S$-D and HypC$_S$	Strep-tag II C-terminal of HypC	100 mM Tris/HCl, pH 8 at 4°C, 100 mM NaCl, 0.1 mM DTT	10 CV 100 mM Tris/HCl, pH 8 at 4°C, 100 mM NaCl, 0.1 mM DTT	5 CV 100 mM Tris/HCl, pH 8 at 4°C, 100 mM NaCl, 0.1 mM DTT, + 3 mM desthiobiotin	10
holo-HypC$_S$-D	Strep-tag II C-terminal of HypC	100 mM Tris/HCl, pH 8 at 4°C, 100 mM NaCl, 0.1 mM DTT	10 CV 100 mM Tris/HCl, pH 8 at 4°C, 100 mM NaCl, 0.1 mM DTT	5 CV 100 mM Tris/HCl, pH 8 at 4°C, 100 mM NaCl, 0.1 mM DTT, + 3 mM desthiobiotin	10
holo-HypC$_H$-D and $_H$HypD	His$_6$-tag C-terminal of HypC or N-terminally of HypD, respectively	100 mM Tris/HCl, pH 8 at 4°C, 100 mM NaCl, 0.1 mM DTT	1) 3 CV 100 mM Tris/HCl, pH 8 at 4°C, 100 mM NaCl, 0.1 mM DTT<br>2) 3 CV 100 mM Tris/HCl, pH 8 at 4°C, 100 mM NaCl, 0.1 mM DTT, + 10 mM imidazole<br>3) 3 CV 100 mM Tris/HCl, pH 8 at 4°C, 100 mM NaCl, 0.1 mM DTT, + 20 mM imidazole	5 CV 100 mM Tris/HCl, pH 8 at 4°C, 100 mM NaCl, 0.1 mM DTT, + 250 mM imidazole	10
apo-$_S$HoxC	Strep-tag II N-terminal of HoxC	50 mM Tris/HCl, pH 8 at 4°C, 200 mM NaCl, 0.1 mM DTT	10 CV 50 mM Tris/HCl, pH 8 at 4°C, 200 mM NaCl, 0.1 mM DTT	5 CV 50 mM Tris/HCL, pH 8 at 4°C, 200 mM NaCl, 0.1 mM DTT, + 3 mM desthiobiotin	30

[a] Strep-Tactin Superflow® resin (high-capacity, iba-Lifesciences) and HisPur™ Ni-NTA resin (Thermo Scientific) were used for purification of Strep-tagged and His-tagged proteins, respectively.

[b] Resuspension buffer additionally contained one unit of protease inhibitor cocktail (Complete, EDTA-free, Roche) and a tip of a spatula of DNaseI (Roche) per 50 mL of buffer. DTT – dithiothreitol

[c] Purification of apo- and holo-HypC$_S$-D samples for crosslinking MS was conducted with 50 mM MOPS/NaOH pH 7.4 at 4°C, 150 mM NaCl and the same additives in resuspension, wash and elution buffer as listed above.

[d] Ultracel® Amicon® Ultra 15 mL centrifugal filter units were used for concentration.

Table S2. Plasmids used in this study.

Plasmid	Characteristics	Protein	Reference
pTHypDC$_{strep}$	pT7-7, *hypD, hypC$_{Strep}$,* Amp^R	apo-HypC$_S$-D	[10]
pTHypDEFC$_{strep}$	pT7-7, *hypD, hypE, hypC$_{Strep}$, hypF,* Amp^R	holo-HypC$_S$-D	[10]
pTS15	pT7-7, *hypD, hypE, hypC$_{His}$, hypF,* Amp^R	holo-HypC$_H$-D	This study
pTS17	pT7-7, *$_{Strep}$hoxC,* Amp^R	apo-$_S$HoxC	This study
pJH3647	P$_{SH}$-*hoxB$_{stop\text{-}His\text{-}Strep}$HoxC*	$_S$HoxC	[11]
pHart13	pT7-7, h*yp$_{His}$D,* Amp^R	$_H$HypD	This study
pAKW19	pT7-7, *hypC$_{Strep}$,* Amp^R	HypC$_S$	This study

Table S3. Mössbauer parameters of apo- and holo-HypC$_S$-D.

Sample	Species	Isomer shift, δ, (mm s^{-1})	Quadrupole splitting, ΔEq (mm s^{-1})
holo-HypC$_S$-D	Fe^{II}(CN)$_2$(CO)	0.25	1.60
	[4Fe–4S]$^{2+}$ Fe$^{2.5+}$-Fe$^{2.5+}$(I)	0.31	0.60
	[4Fe–4S]$^{2+}$ Fe$^{2.5+}$-Fe$^{2.5+}$(II)	0.35	1.03
apo-HypC$_S$-D	[3Fe–4S]	0.07	0.73
	[4Fe–4S]$^{2+}$ Fe$^{2.5+}$-Fe$^{2.5+}$(I)	0.45	0.89
	[4Fe–4S]$^{2+}$ Fe$^{2.5+}$-Fe$^{2.5+}$(II)	0.46	1.20

Table S4. CB-Dock2 analysis of ATP-binding sites in HypE from *T. kodakarensis.*[12]

Cavity	Vina Score (kcal/mol)	Nearby residues
A	-7.6	Gly12 Met16 Glu17 Leu20 Leu38 Leu41 Asp42 Asp43 Ile58 Asp59 Asp84 Asp158 Lys220 Asp221 Pro222 Thr223 Arg224 Asn229 Glu233 Lys237
B	-7.3	Gly70 Arg75 Glu123 Ser170 Gly171 Thr172 Asp175 Lys194 Ser195 Asp196 Val197 Ala198 Ile200 Arg252 Gly273 Lys274 Glu295 Lys296 Gly297 Arg298 Asn299
C	-8.8	Gly12 Gly13 Glu14 Met16 Glu17 Ile36 Gly37 Leu38 Ala40 Leu41 Asp42 Asp43 Gly44 Ala45 Thr57 Ile58 Asp59 Asp84 Asp158 Lys220 Asp221 Thr223 Arg224 Asn229 Glu233 Lys237
D	-5.7	Gly164 Asp165 Ala166 Val167 Leu243 Val244 Arg245 Asp248 Val279 Arg281 Ala284 Glu285 Ile302 Ile303 Gly304 Glu305 Glu317
E	-6.2	Lys52 Gly89 Ala90 Glu91 Ile151 Ala152 Glu153 His154 Trp201 Lys205 Ala208 Glu209 Gly212 Trp213 Glu214

Table S5. CB-Dock2 analysis of ATP-binding sites in *Tk*HypCD[a].

Cavity	Vina Score (kcal/mol)	Nearby residues
A	-8.3	HypC: His45 Thr46<br>HypD: Val37 Cys38 Thr40 Gly65 Cys66 Pro67 Val68 Phe98 Asp100 Val124 Tyr125 Gly126 Gly148 Phe149 Thr151 Thr152 Pro200 His202 Val203 Phe228 Ser357 Tyr358 Glu359
B	-5.7	HypD: Glu42 Val45 Thr46 Arg47 His48 Gly49 Ile50 Arg51 Ser52 Leu53 Phe365 Tyr368 Gly369 Val370 Leu371 Phe372
C	-6.3	HypC: Ala58 Met59 Ile61 Leu62 Trp65<br>HypD: Phe5 Tyr8 Val209 Met235 Arg250 Ile251 Ile252 Asn253 Glu254 Tyr255 Glu256 Arg257
D	-5.5	HypD: Glu150 Leu179 Pro181 Pro182 Ile207 Gly208 Lys210 Gly211 Tyr214 Glu262 Gln268 Ile271 Asp272 Phe275 Glu276 Val277 Leu293 Arg296
E	-5.9	HypD: Glu150 Leu179 Thr180 Pro181 Pro182 Glu185 Ile207 Gly211 Tyr214 Ile271 Asp272 Phe275 Glu276 Val277 Pro289 Lys290 Leu293 Arg296

[a] X-ray coordinates of *Tk*HypCD: PDB 3VYR;[4] DrugBank ID of ATP: DB00171.

Table S6. Nucleotide solutions used for the *in vitro* transfer assay.

Component	Company	Solution
Adenosinetriphosphate (ATP)	Roche	dilithium salt, 100 mM solution, pH 7
Guanosintriphosphate (GTP)	Roche	dilithium salt, 100 mM solution, pH 7
β,γ,Methyleneadenosine 5’-triphosphate	Sigma Aldrich	disodium salt, 100 mM solution, in 1 M MOPS/KOH, pH 7.4

Table S7. CB-Dock2 analysis of ATP-binding sites in the AlphaFold 2-predicted *Ec*HypCD structure carrying native HypC[a].

Cavity	Vina Score (kcal/mol)	Nearby residues [b]
A	-5.7	HypC: Val77 Pro79 Asp80<br>HypD: Arg101 Pro345 Gln346 Met353 Val354 Ser356 Ala361 Ala362 Tyr364 Gln365 Gln368
B	-8.0	HypD: Pro75 Gly77 Arg78 Thr81 Cys173 Gln174 His175 Ile176 Asp279 Ser280 Glu281 Gly292 Val293 His294 Glu305 Pro310 Ala311 Pro312 Gln313 Gln314
C	-6.0	HypD: Pro34 Asp54 Gln55 Leu56 Leu57 Pro58 Glu59 Asn60 Val61 Glu62 Phe63 Lys332 Trp363 Arg367 Glu370
D	-4.7	HypD: Glu148 Thr177 Leu178 Ile179 Pro180 Val205 Ile206 Gly207 Ala210 Ala261 Gln267 Gln268 Ile270 Ala271 Cys275 Val276
E	-5.2	HypC: Val77<br>HypD: Arg2 Phe3 Glu6 Tyr364 Gln365 Tyr366 Arg367 Gln368 Gln369

[a] DrugBank ID of ATP: DB00171.

[b] The primary methionine (Met1) was omitted from AlphaFold models, as this residue is usually cleaved by the methionine aminopeptidase as part of post-translational modifications and is therefore not present in the protein.

Table S8. CB-Dock analysis of ATP-binding sites in the AlphaFold 2-predicted *Ec*HypCD structure carrying an N-terminally truncated version of HypC[a].

Cavity	Vina Score (kcal/mol)	Nearby residues
A	-8.3	HypC: Val5 Leu49 Val50 His51 Val52 Leu71 Met74 Phe75 Val81 Leu84<br>HypD: Val40 Cys41 Gly42 Gly43 Gly68 Pro70 Phe96 Asp98 Leu145 Gly146 Phe147 Thr149 Thr150 Pro199 His201 Val202 Phe227 Glu357
B	-7.9	HypD: Pro75 Gly77 Arg78 Thr81 Glu84 Phe172 Cys173 Gln174 His175 Ile176 Asp279 Ser280 Glu281 Trp282 Ser291 Gly292 Val293 His294 Glu305 Pro310 Ala311 Pro312 Gln313 Gln314 Val315
C	-6.0	HypD: Pro34 Asp54 Gln55 Leu56 Leu57 Pro58 Glu59 Asn60 Val61 Glu62 Phe63 Lys332 Trp363 Arg367 Glu370
D	-5.6	HypD: Glu148 Met151 Thr177 Ile179 Val205 Ile206 Gly207 Thr208 Asp209 Ala210 Asp260 Ala261 Gly262 Leu264 Gln267 Ile270 Val28
E	-4.8	HypD: Arg2 Phe3 Glu6 Lys50 Gln365 Tyr366 Arg367 Gln368 Gln369

[a] The HypC sequence submitted for analysis lacked Met1, Cys2 and Ile3. DrugBank ID of ATP: DB00171.

Table S9. CB-Dock analysis of ATP-binding sites in the AlphaFold 2-predicted *Tk*HypCD structure carrying native HypC[a].

Cavity	Vina Score (kcal/mol)	Nearby residues[b]
A	-6.4	HypC: Cys2 Leu3 Ala4 Val5 Val44 His45 Thr46<br>HypD: Asp43 Arg47 Phe98 Gly99 Asp100 Ile123 Val124 Tyr125 Tyr356 Glu359 Gln363 Ile364 Lys367 Tyr368
B	-6.2	HypC: Leu3 Val5 Pro6 Leu53<br>HypD: Glu3 Phe5 Arg9 Thr40 Asp43 Thr44 Arg47 His48 His202 Gly227 Phe228 Glu229 Asp232 Tyr255 Arg257
C	-5.5	HypD: Arg11 Ala14 Met15 Val18 Thr46 Arg47 His48 Gly49 Ile50 Ser52 Leu53 Pro230 Asn231 Leu234 Tyr368 Gly369 Leu371 Phe372
D	-5.4	HypD: Tyr102 Lys103 Ile104 Pro105 Thr106 Pro107 Met108 Gly109 Ser110 Ala112 Asp113 Glu320 Arg348 His349 Pro350 Val351 Gly352
E	-5.4	HypD: Arg9 Ser10 Arg11 Ala14 Thr46 Arg47 His48 Gly49 Ile50 Ser52 Leu53 Pro230 Leu234 Tyr368 Gly369 Leu371 Phe372

[a] DrugBank ID of ATP: DB00171.

[b] The primary methionine (Met1) was omitted from AlphaFold models, as this residue is usually cleaved by the methionine aminopeptidase as part of post-translational modifications and is therefore not present in the protein.

Table S10. Values from CB-Dock analysis using the AlphaFold 2-predicted *Tk*HypCD carrying an N-terminally truncated version of HypC and ATP [a].

Cavity	Vina Score (kcal/mol)	Nearby residues
A	-7.6	HypC: Val5 Val44 His45 Thr46<br>HypD: Cys38 Gly39 Thr40 Asp43 Arg47 Gly65 Cys66 Pro67 Gly148 Phe149 Thr151 Thr152 Pro200 His202 Val203 Phe228 Tyr358 Glu359
B	-7.0	HypC: Val5 Pro6 Ile43 Val44 His45 Thr46 Gly47 Leu53<br>HypD: Phe5 Arg9 Thr40 Thr44 Tyr125 Thr151 His202 Phe228 Glu229 Tyr255 Arg257 Ala258 Tyr358 Glu359
C	-5.7	HypD: Arg11 Ala14 Met15 Val18 Thr46 Arg47 His48 Gly49 Ile50 Ser52 Leu53 Pro230 Leu234 Tyr368 Gly369 Leu371 Phe372
D	-5.3	HypD: Lys103 Ile104 Pro105 Thr106 Pro107 Gly109 Ser110 Ala112 Asp113 Glu320 Arg348 His349 Pro350 Val356
E	-5.9	HypD: Tyr8 Arg9 Ser10 Arg11 Ala14 Met15 Thr46 Arg47 His48 Gly49 Ile50 Ser52 Leu53 Pro230 Asn231 Leu234 Tyr368 Gly369 Leu371 Phe372

[a] The HypC sequence submitted for analysis lacked Met1, Cys2 and Ile3. DrugBank ID of ATP: DB00171.

Table S11. Primers used in this study.

Name	5'-3' Sequence
pT7_for	GCTTATCGATGATAAGCTGTC
pT7_rev	TGTATATCTCCTTCTTAAAGTTAAAC
Strep-HoxC_rev	GACAGCTTATCATCGATAAGCGCTCAATGCACGGTGCACAC
Strep-HoxC_for	GTTTAACTTTAAGAAGGAGATATACAATGGCTAGCTGGAGCCACC
2_Amp_rev	GATACCGCGAGACCCAC
3_Amp_for	GATAAATCTGGAGCCGGTG
HisHypCDrev_2	TTAGTGATGATGATGATGATGGCCGCTGCTTTTTTCCTCGCCATACAACAGC
HisHypCDfor_2	AGCAGCGGCCATCAATCATCATCATCACTAAGGGGATCCTCTAGAGTCGA
HypDrev	GGCATTGATGCGGCTTAC
seqHypD	GGTGAGGTATTAACGGGC
NdeI_his6_ec_HypD_fwd_57	TAGTCATATGCATCATCACCATCACCACCGTTTTGTTGATGAATAT
Ec_HypD_HinIII_rev_58	TAGTAAGCTTTCACGCTTCACTCTCCTGCTGACG
AKWp38	GAATAACTCAAACGTAAGCTTATCGATG
AKWp39	CTATGCACATATGTATATCTCCTTCTTAAAGTTAAAC
AKWp40	AGATATACATATGTGCATAGGCGTTCCC
AKWp41	AGCTTACGTTTGAGTTATTCCCGCCATC

Supplementary References

(1) Stripp, S. T.; Soboh, B.; Lindenstrauss, U.; Braussemann, M.; Herzberg, M.; Nies, D. H.; Sawers, R. G.; Heberle, J. HypD is the scaffold protein for Fe-$(CN)_2CO$ cofactor assembly in [NiFe]-hydrogenase maturation. *Biochemistry* **2013**, *52*, 3289–3296.

(2) Caserta, G.; Hartmann, S.; van Stappen, C.; Karafoulidi-Retsou, C.; Lorent, C.; Yelin, S.; Keck, M.; Schoknecht, J.; Sergueev, I.; Yoda, Y.; Hildebrandt, P.; Limberg, C.; DeBeer, S.; Zebger, I.; Frielingsdorf, S.; Lenz, O. Stepwise assembly of the active site of [NiFe]-hydrogenase. *Nat. Chem. Biol.* **2023**, *19*, 498–506.

(3) Blokesch, M.; Böck, A. Properties of the [NiFe]-hydrogenase maturation protein HypD. *FEBS Lett.* **2006**, *580*, 4065–4068.

(4) Watanabe, S.; Matsumi, R.; Atomi, H.; Imanaka, T.; Miki, K. Crystal structures of the HypCD complex and the HypCDE ternary complex: transient intermediate complexes during [NiFe] hydrogenase maturation. *Structure* **2012**, *20*, 2124–2137.

(5) Albareda, M.; Palacios, J.-M.; Imperial, J.; Pacios, L. F. Computational study of the $Fe(CN)_2CO$ cofactor and its binding to HypC protein. *J. Phys. Chem. B.* **2013**, *117*, 13523–13533.

(6) Tominaga, T.; Watanabe, S.; Matsumi, R.; Atomi, H.; Imanaka, T.; Miki, K. Crystal structures of the carbamoylated and cyanated forms of HypE for [NiFe] hydrogenase maturation. *Proc. Natl. Acad. Sci. U. S. A.* **2013**, *110*, 20485–20490.

(7) Schneider, T. D.; Stephens, R. M. Sequence logos: a new way to display consensus sequences. *Nucleic Acids Res.* **1990**, *18*, 6097–6100.

(8) Kornberg, A.; Pricer, W. E., JR. Ezymatic phosphorylation of adenosine and 2,6-diaminopurine riboside. *J. Biol. Chem.* **1951**, *193*, 481–495.

(9) Nutschan, K.; Golbik, R. P.; Sawers, R. G. The iron-sulfur-containing HypC-HypD scaffold complex of the [NiFe]-hydrogenase maturation machinery is an ATPase. *FEBS Open Bio* **2019**, *9*, 2072–2079.

(10) Blokesch, M.; Albracht, S. P. J.; Matzanke, B. F.; Drapal, N. M.; Jacobi, A.; Böck, A. The complex between hydrogenase-maturation proteins HypC and HypD is an intermediate in the supply of cyanide to the active site iron of [NiFe]-hydrogenases. *J. Mol. Biol.* **2004**, *344*, 155–167.

(11) Caserta, G.; Lorent, C.; Ciaccafava, A.; Keck, M.; Breglia, R.; Greco, C.; Limberg, C.; Hildebrandt, P.; Cramer, S. P.; Zebger, I.; Lenz, O. The large subunit of the regulatory [NiFe]-hydrogenase from *Ralstonia eutropha* - a minimal hydrogenase? *Chem. Sci.* **2020**, *11*, 5453–5465.

(12) Watanabe, S.; Matsumi, R.; Arai, T.; Atomi, H.; Imanaka, T.; Miki, K. Crystal structures of [NiFe] hydrogenase maturation proteins HypC, HypD, and HypE: insights into cyanation reaction by thiol redox signaling. *Mol. Cell* **2007**, *27*, 29–40.
