# Supplementary figures and images for: ATP-Triggered Fe(CN)2CO Synthon Transfer from the Maturase HypCD to the Active Site of Apo-[NiFe]-Hydrogenase
Source: J Am Chem Soc. 2024 Nov 4;146(45):30976–89. doi: 10.1021/jacs.4c09791 (PMC11565642; doi:10.1021/jacs.4c09791)

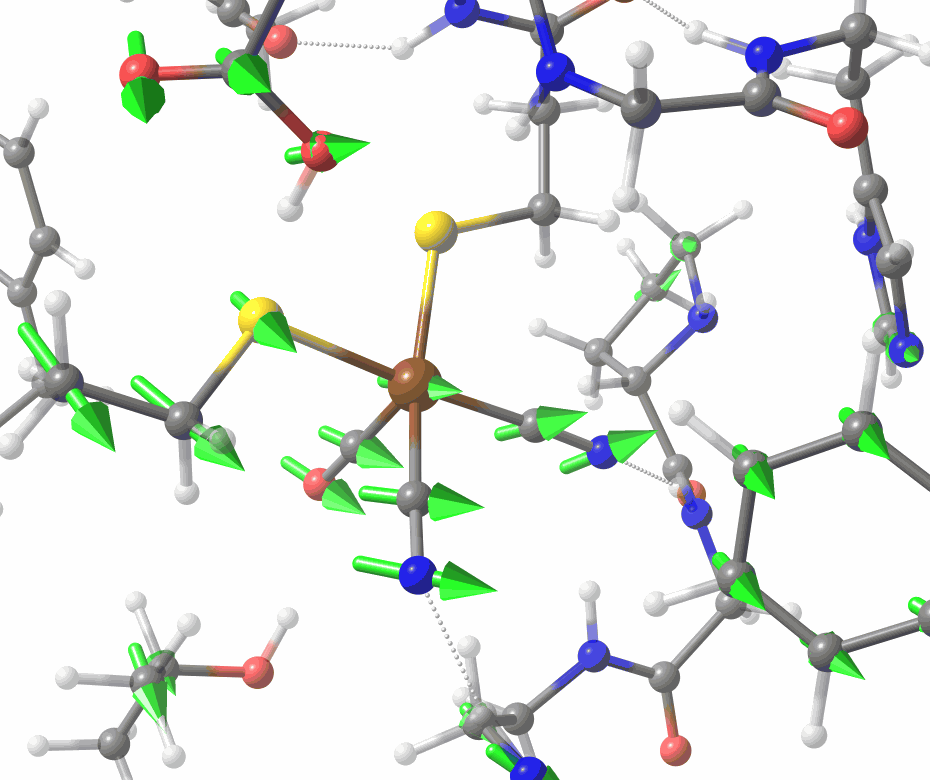

Supplement: Supplementary file 2 — ja4c09791_si_002.zip [file ja4c09791_si_002.zip › Supplementary Data III/FeCN2CO-EcHypCD_0022_cm-1.gif]

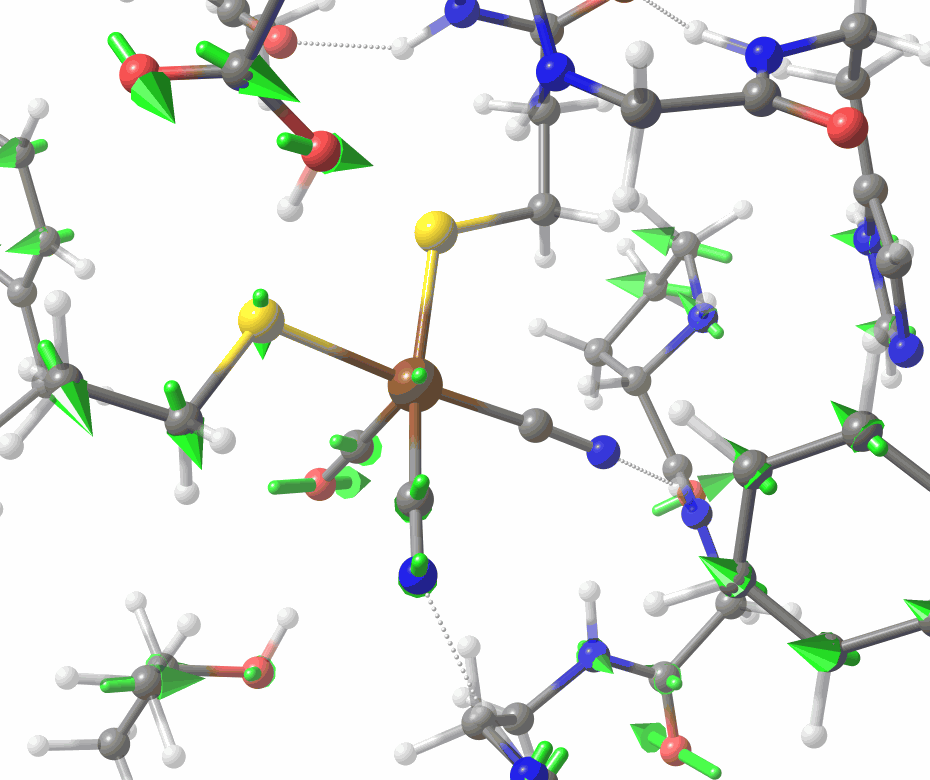

Supplement: Supplementary file 2 — ja4c09791_si_002.zip [file ja4c09791_si_002.zip › Supplementary Data III/FeCN2CO-EcHypCD_0050_cm-1.gif]

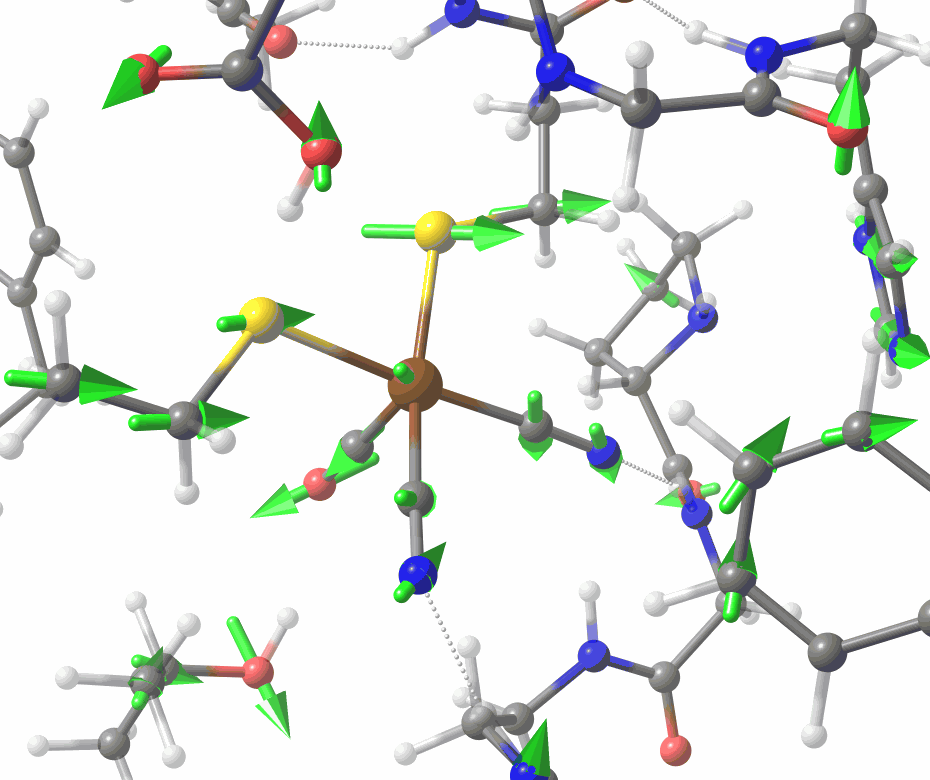

Supplement: Supplementary file 2 — ja4c09791_si_002.zip [file ja4c09791_si_002.zip › Supplementary Data III/FeCN2CO-EcHypCD_0080_cm-1.gif]

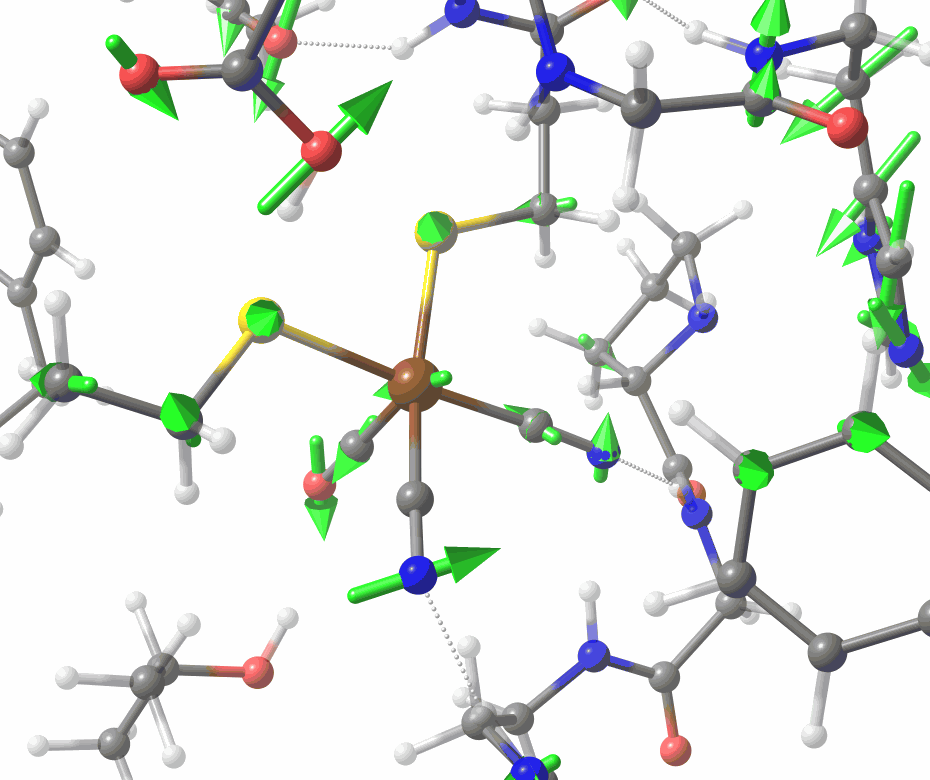

Supplement: Supplementary file 2 — ja4c09791_si_002.zip [file ja4c09791_si_002.zip › Supplementary Data III/FeCN2CO-EcHypCD_0132_cm-1.gif]

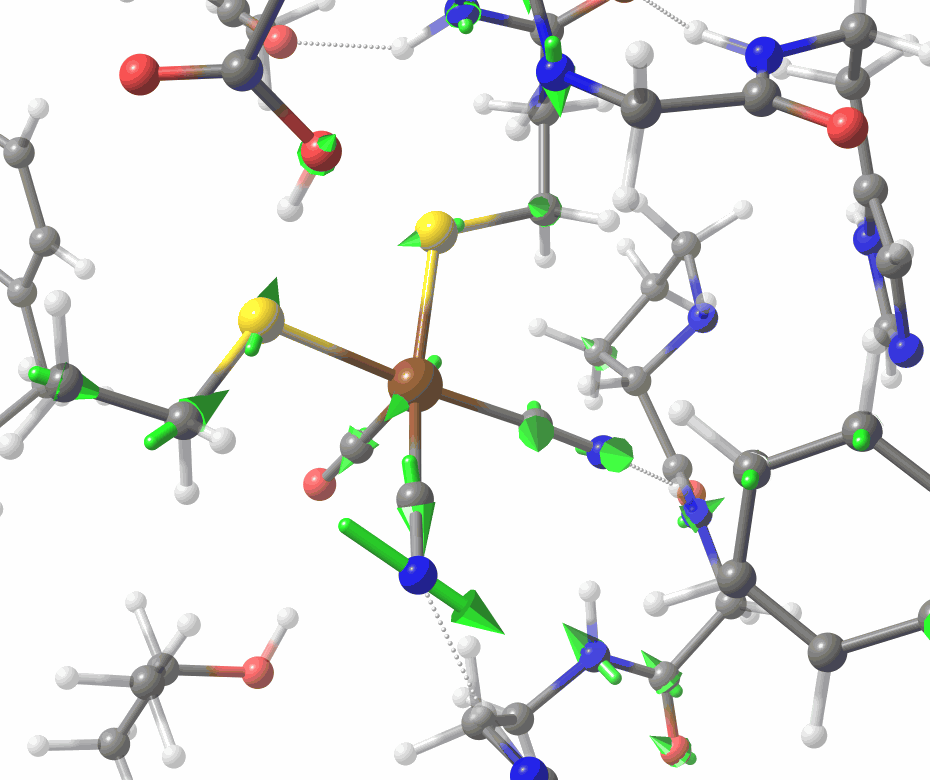

Supplement: Supplementary file 2 — ja4c09791_si_002.zip [file ja4c09791_si_002.zip › Supplementary Data III/FeCN2CO-EcHypCD_0199_cm-1.gif]

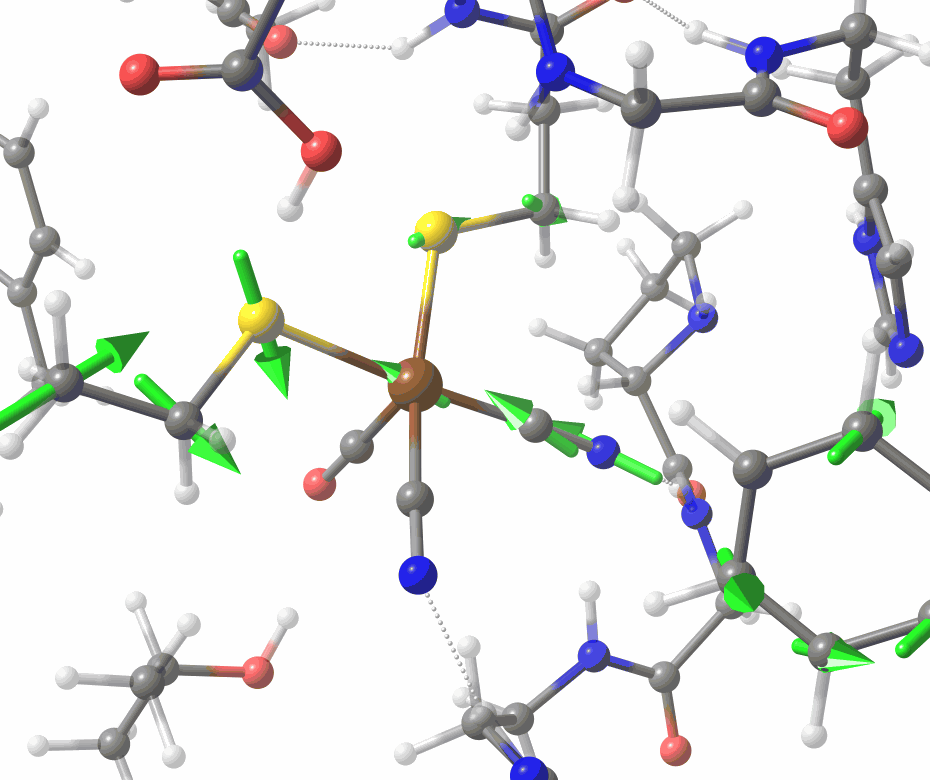

Supplement: Supplementary file 2 — ja4c09791_si_002.zip [file ja4c09791_si_002.zip › Supplementary Data III/FeCN2CO-EcHypCD_0256_cm-1.gif]

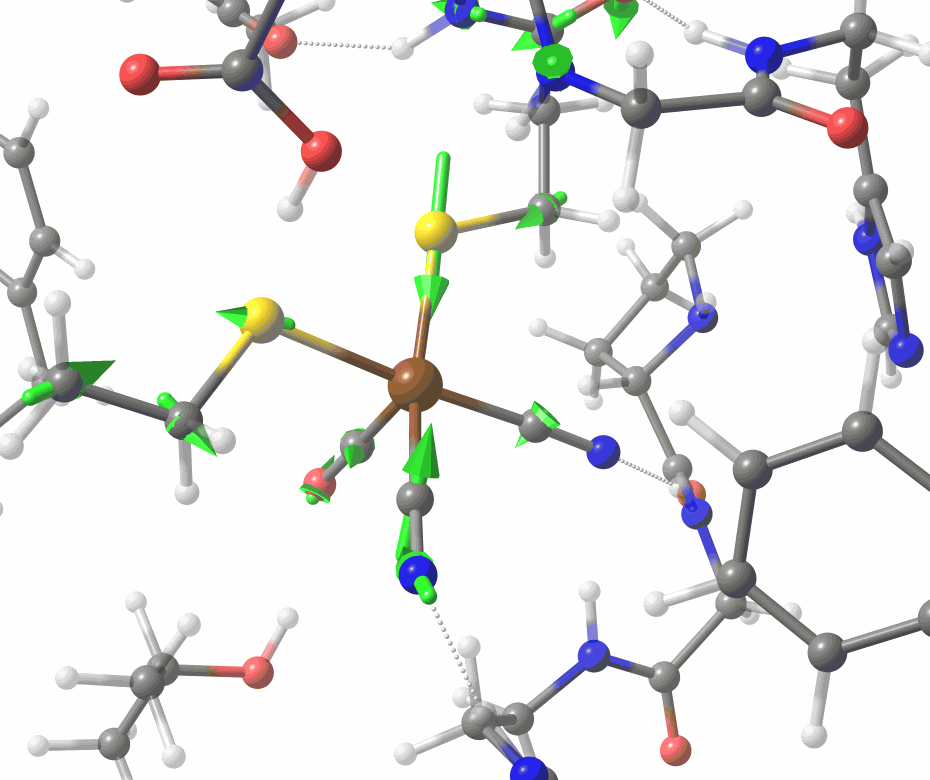

Supplement: Supplementary file 2 — ja4c09791_si_002.zip [file ja4c09791_si_002.zip › Supplementary Data III/FeCN2CO-EcHypCD_0366_cm-1.gif]

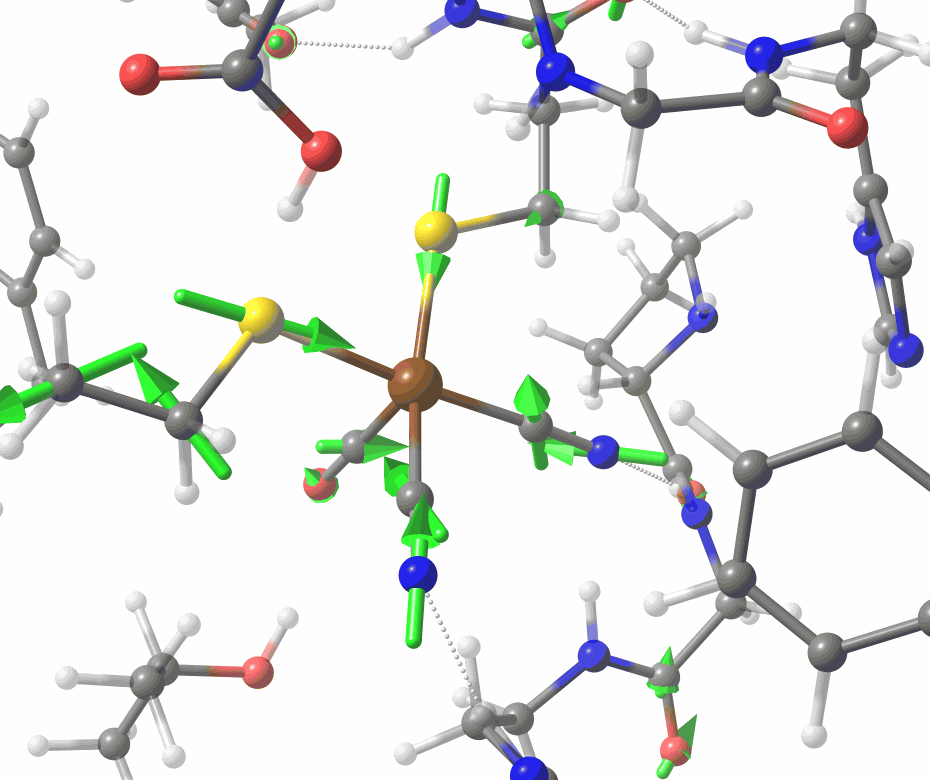

Supplement: Supplementary file 2 — ja4c09791_si_002.zip [file ja4c09791_si_002.zip › Supplementary Data III/FeCN2CO-EcHypCD_0390_cm-1.gif]

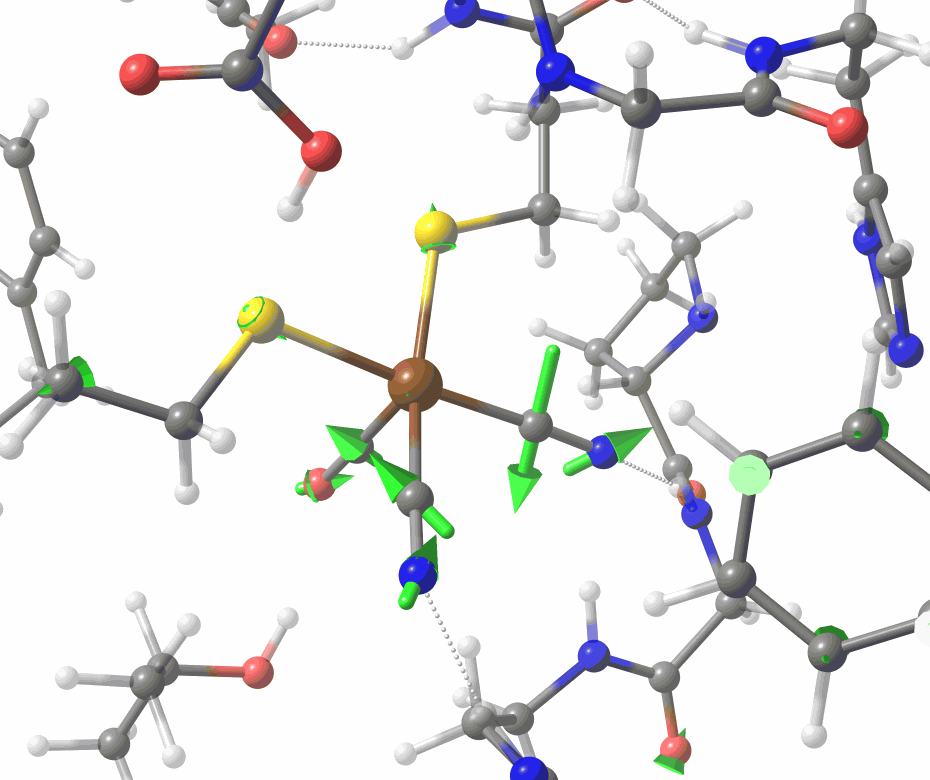

Supplement: Supplementary file 2 — ja4c09791_si_002.zip [file ja4c09791_si_002.zip › Supplementary Data III/FeCN2CO-EcHypCD_0451_cm-1.gif]

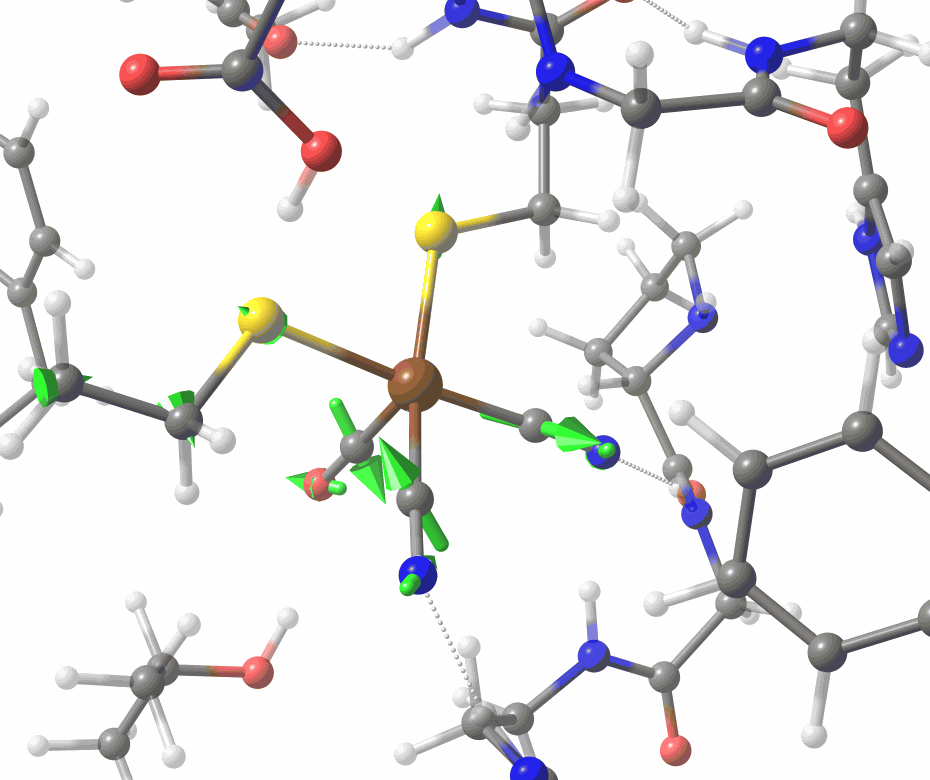

Supplement: Supplementary file 2 — ja4c09791_si_002.zip [file ja4c09791_si_002.zip › Supplementary Data III/FeCN2CO-EcHypCD_0462_cm-1.gif]

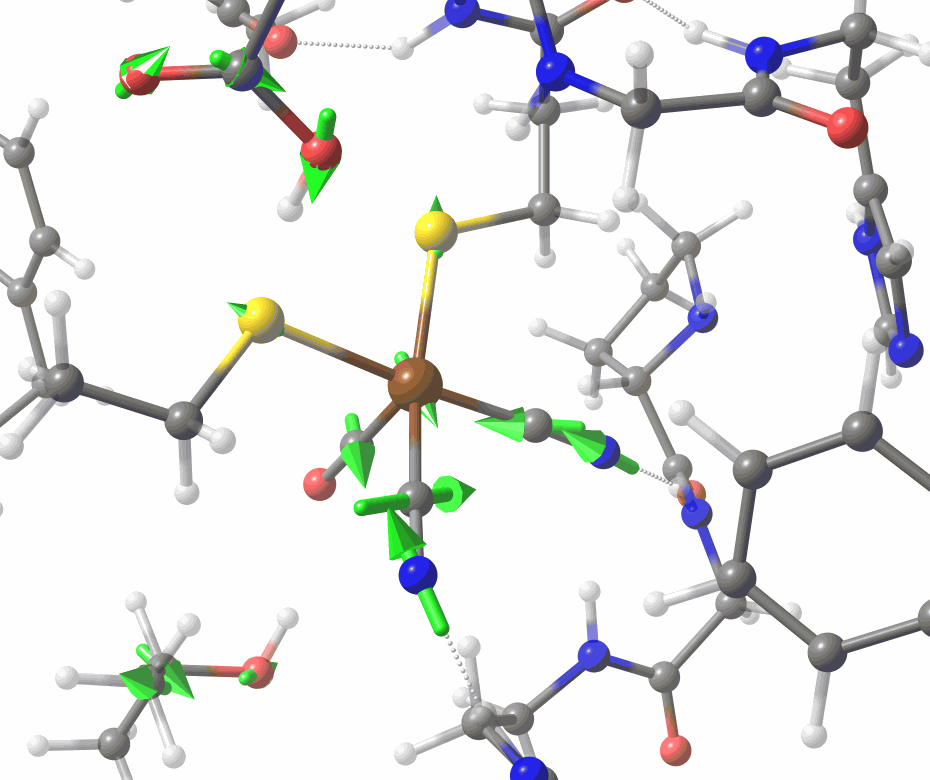

Supplement: Supplementary file 2 — ja4c09791_si_002.zip [file ja4c09791_si_002.zip › Supplementary Data III/FeCN2CO-EcHypCD_0485_cm-1.gif]

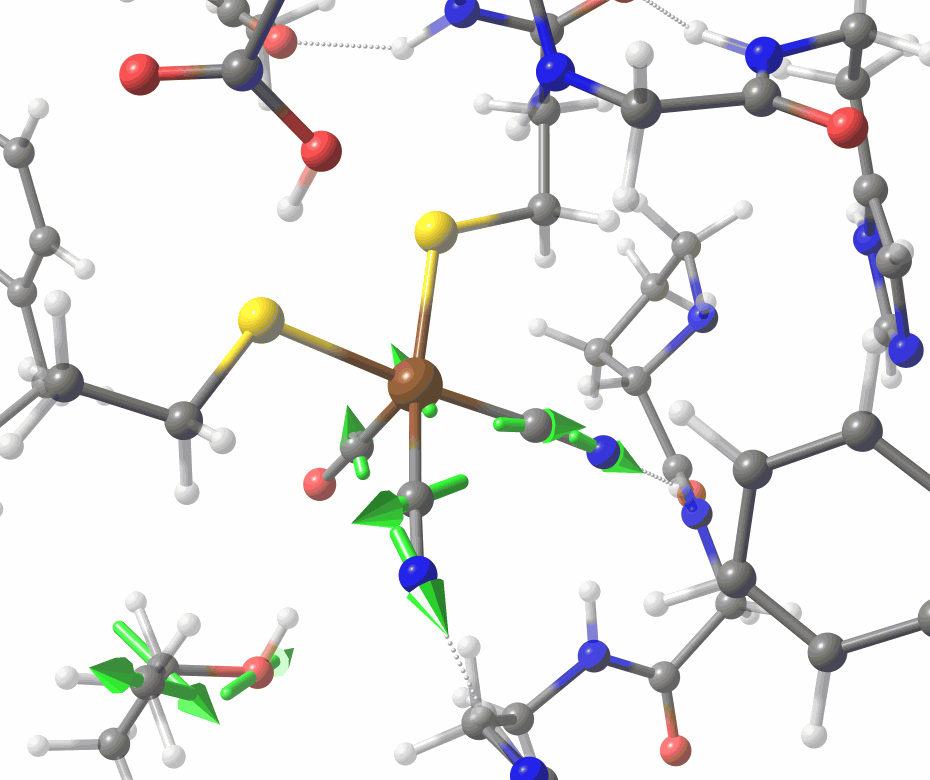

Supplement: Supplementary file 2 — ja4c09791_si_002.zip [file ja4c09791_si_002.zip › Supplementary Data III/FeCN2CO-EcHypCD_0489_cm-1.gif]

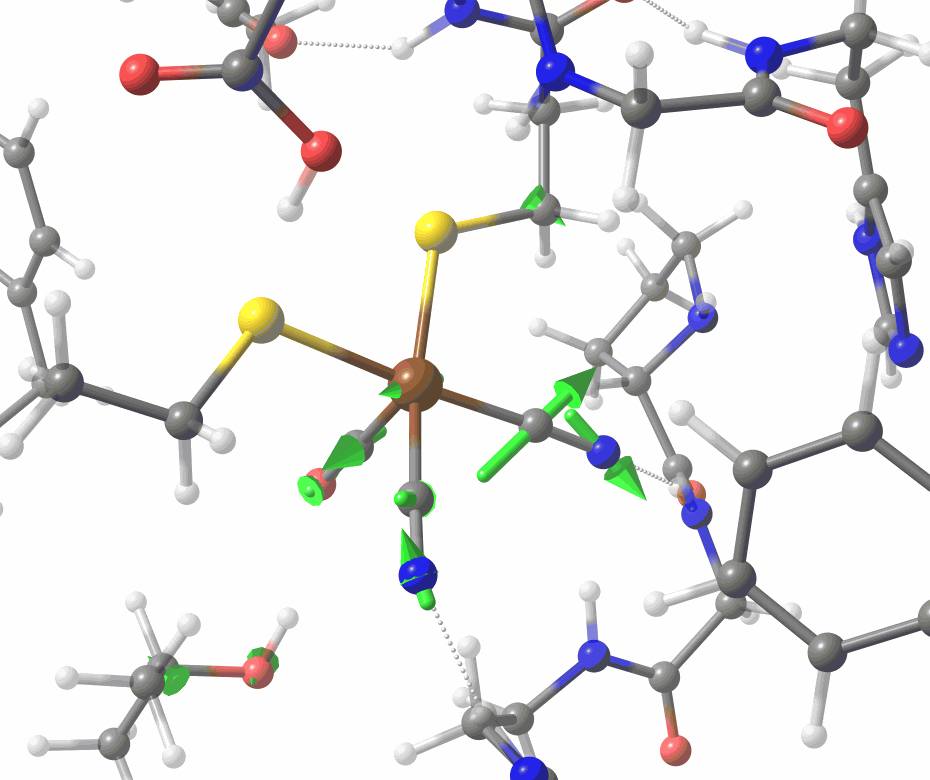

Supplement: Supplementary file 2 — ja4c09791_si_002.zip [file ja4c09791_si_002.zip › Supplementary Data III/FeCN2CO-EcHypCD_0509_cm-1.gif]

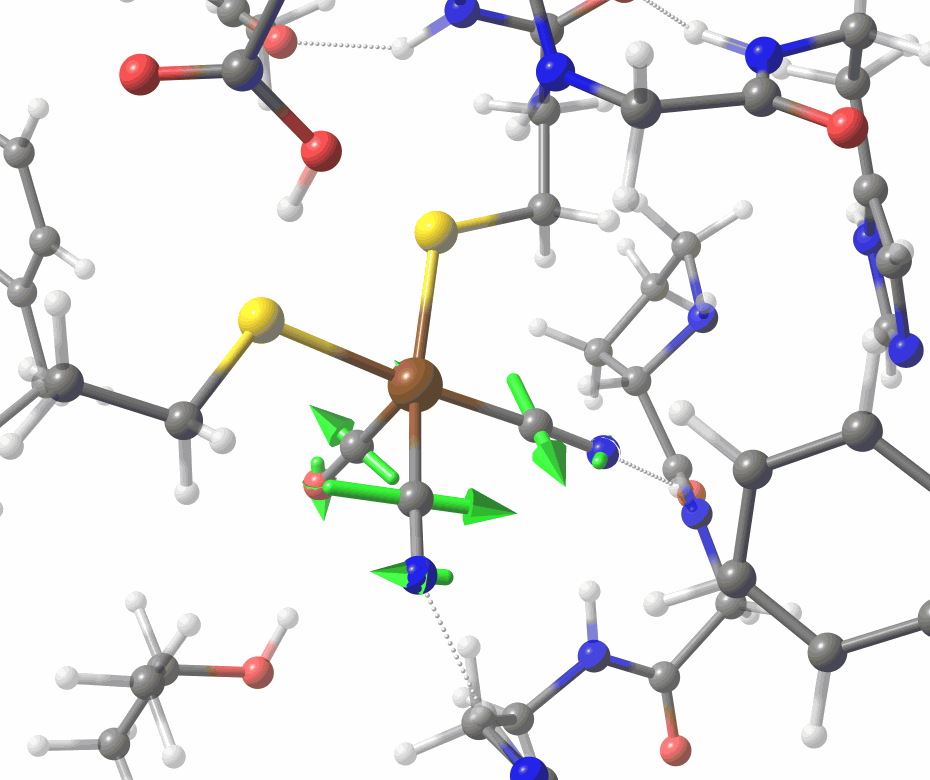

Supplement: Supplementary file 2 — ja4c09791_si_002.zip [file ja4c09791_si_002.zip › Supplementary Data III/FeCN2CO-EcHypCD_0561_cm-1.gif]

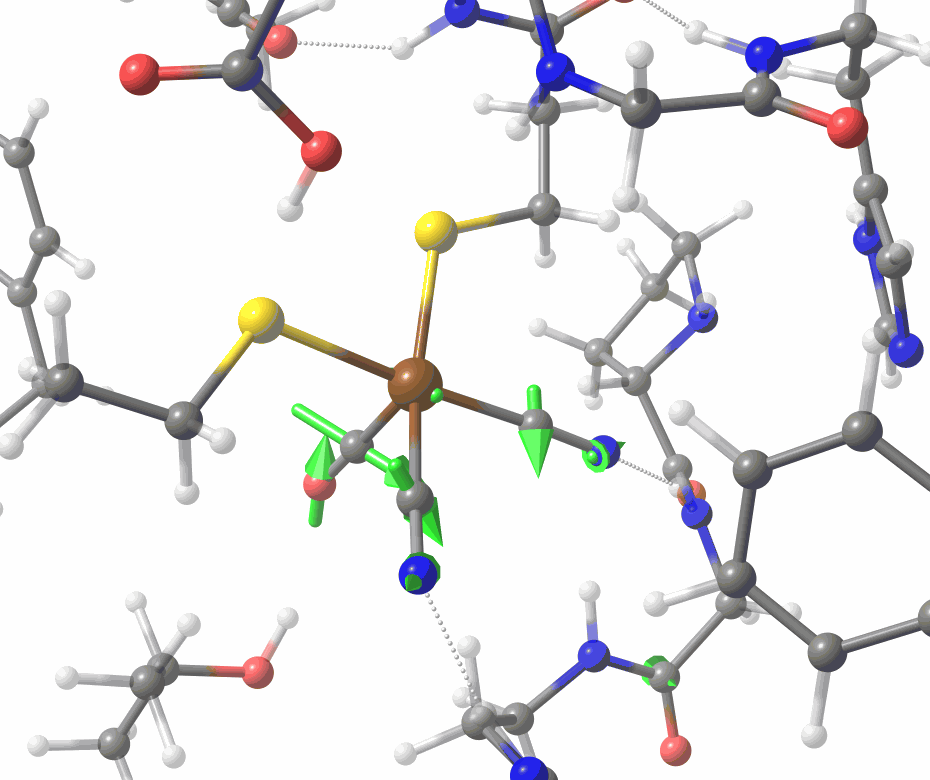

Supplement: Supplementary file 2 — ja4c09791_si_002.zip [file ja4c09791_si_002.zip › Supplementary Data III/FeCN2CO-EcHypCD_0591_cm-1.gif]

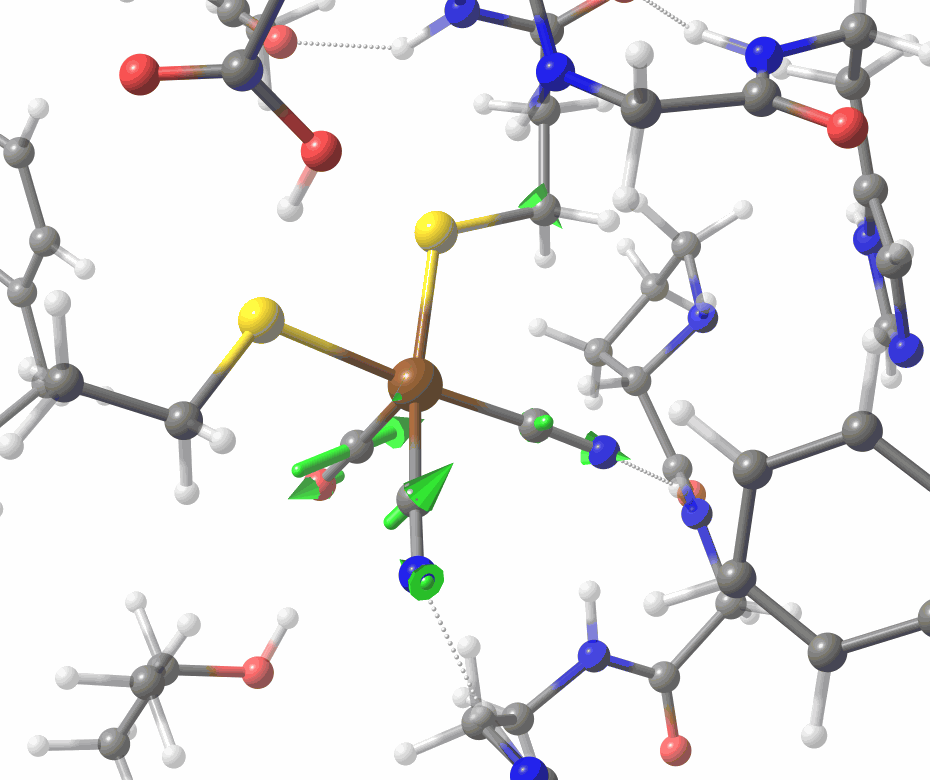

Supplement: Supplementary file 2 — ja4c09791_si_002.zip [file ja4c09791_si_002.zip › Supplementary Data III/FeCN2CO-EcHypCD_0594_cm-1.gif]

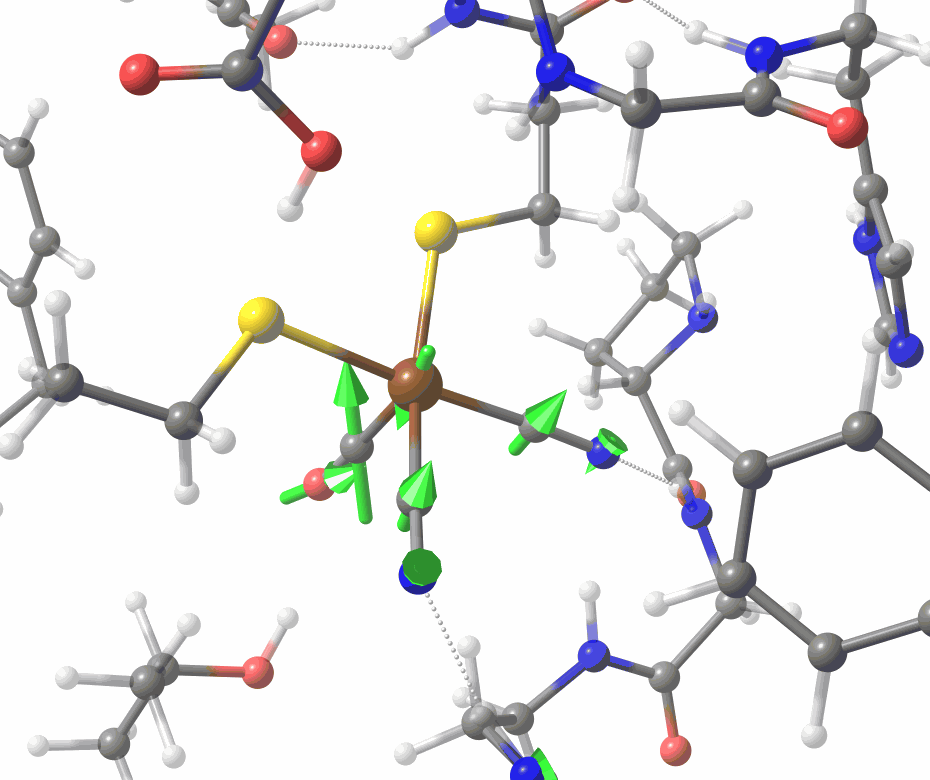

Supplement: Supplementary file 2 — ja4c09791_si_002.zip [file ja4c09791_si_002.zip › Supplementary Data III/FeCN2CO-EcHypCD_0625_cm-1.gif]
